# Modeling reduced contractility and impaired desmosome assembly due to plakophilin-2 deficiency using isogenic iPS cell-derived cardiomyocytes

**DOI:** 10.1016/j.stemcr.2021.12.016

**Published:** 2022-01-20

**Authors:** Hiroyuki Inoue, Satoki Nakamura, Shuichiro Higo, Mikio Shiba, Yasuaki Kohama, Takumi Kondo, Satoshi Kameda, Tomoka Tabata, Shota Okuno, Yoshihiko Ikeda, Junjun Li, Li Liu, Satoru Yamazaki, Maki Takeda, Emiko Ito, Seiji Takashima, Shigeru Miyagawa, Yoshiki Sawa, Shungo Hikoso, Yasushi Sakata

**Affiliations:** 1 Department of Cardiovascular Medicine, Osaka University Graduate School of Medicine, Suita, Osaka 565-0871, Japan; 2 Osaka Police Hospital, Osaka 543-0035, Japan; 3 Department of Medical Therapeutics for Heart Failure, Osaka University Graduate School of Medicine, Suita, Osaka 565-0871, Japan; 4 Cardiovascular Division, National Hospital Organization, Osaka-Minami Medical Center, Kawachinagano, Osaka 586-8512, Japan; 5 Department of Pathology, National Cerebral and Cardiovascular Center, Suita, Osaka 564-8565, Japan; 6 Department of Cardiovascular Surgery, Osaka University Graduate School of Medicine, Suita, Osaka 565-0871, Japan; 7 Department of Design for Tissue Regeneration, Osaka University Graduate School of Medicine, Suita, Osaka 565-0871, Japan; 8 Department of Molecular Pharmacology, National Cerebral and Cardiovascular Center, Suita, Osaka 564-8565, Japan; 9 Department of Medical Biochemistry, Osaka University Graduate School of Medicine, Suita, Osaka 565-0871, Japan

**Keywords:** plakophilin-2, human induced pluripotent stem cell-derived cardiomyocytes, arrhythmogenic cardiomyopathy, genome editing, adeno-associated virus, desmosome

## Abstract

Loss-of-function mutations in *PKP2*, which encodes plakophilin-2, cause arrhythmogenic cardiomyopathy (AC). Restoration of deficient molecules can serve as upstream therapy, thereby requiring a human model that recapitulates disease pathology and provides distinct readouts in phenotypic analysis for proof of concept for gene replacement therapy. Here, we generated isogenic induced pluripotent stem cell-derived cardiomyocytes (iPSC-CMs) with precisely adjusted expression of plakophilin-2 from a patient with AC carrying a heterozygous frameshift *PKP2* mutation. After monolayer differentiation, plakophilin-2 deficiency led to reduced contractility, disrupted intercalated disc structures, and impaired desmosome assembly in iPSC-CMs. Allele-specific fluorescent labeling of endogenous *DSG2* encoding desmoglein-2 in the generated isogenic lines enabled real-time desmosome-imaging under an adjusted dose of plakophilin-2. Adeno-associated virus-mediated gene replacement of *PKP2* recovered contractility and restored desmosome assembly, which was sequentially captured by desmosome-imaging in plakophilin-2-deficient iPSC-CMs. Our isogenic set of iPSC-CMs recapitulates AC pathology and provides a rapid and convenient cellular platform for therapeutic development.

## Highlights

• Generation of isogenic iPSC-CMs with a precise dose of plakophilin-2
• Modeling reduced contractility and impaired desmosome assembly using iPSC-CMs
• Generation of isogenic iPSC-CMs for desmosome-imaging
• Proof of concept of *PKP2* replacement using isogenic plakophilin-2-deficient iPSC-CMs

## Graphical abstract

**Figure:**
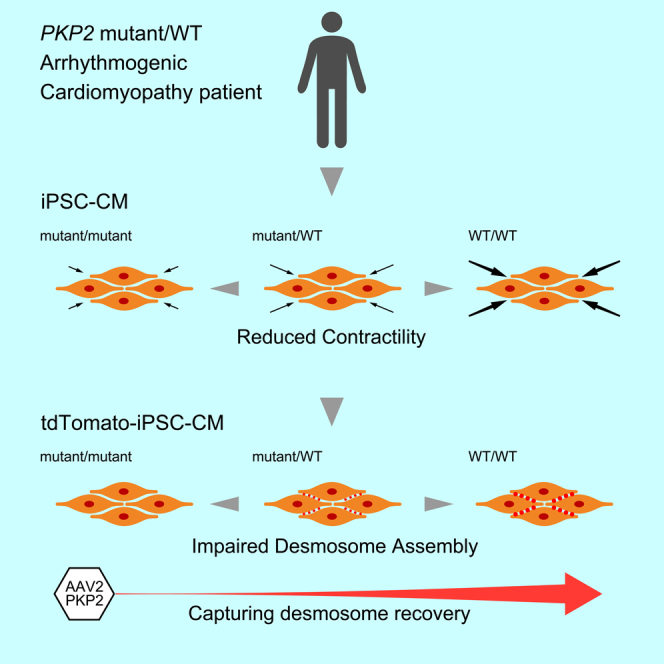

## Teaser

In this article, Higo and colleagues generate isogenic iPS cell-derived cardiomyocytes (iPSC-CMs) with precisely adjusted expression of *PKP2*, modeling reduced contractility and impaired desmosome assembly due to plakophilin-2 deficiency. *PKP2* gene replacement recovered contractility and restored desmosome assembly, which was sequentially captured by desmosome imaging. The isogenic iPSC-CMs recapitulate the pathology of arrhythmogenic cardiomyopathy and provide a rapid and convenient cellular platform for therapeutic development.

## Introduction

Arrhythmogenic cardiomyopathy (AC), defined as an arrhythmogenic heart muscle disorder not explained by ischemic, hypertensive, or valvular disease, is caused by mutations in genes involved in various cellular functions, including desmosomes, ion channels, cytoskeleton, calcium regulation, or sarcomere (Austin et al., 2019; Towbin et al., 2019). AC cases with predominant right ventricular dysfunction have been diagnosed with arrhythmogenic right-ventricular cardiomyopathy (ARVC), which is a rare, life-threatening, intractable disease that leads to adverse ventricular arrhythmia, ventricular dilatation, and reduced cardiac contraction. Focal fatty infiltration, cardiomyocyte loss, and fibrofatty replacement are observed in the heart tissue of patients with AC (Calkins et al., 2017; Haugaa et al., 2016). Analysis of epidemiological data and results from experimental studies using genetically modified mice have revealed that mutations in desmosomal genes (*DSC2*, *DSG2*, *JUP*, *DSP*, and *PKP2*) lead to AC (Awad et al., 2008; Padron-Barthe et al., 2017), with *PKP2* being the most common gene associated with AC. Plakophilin-2 is located in the outer dense plaque of desmosomes, where it interacts with desmosomal cadherins and desmoplakin (Al-Jassar et al., 2013; Padron-Barthe et al., 2017). In mice, the loss of plakophilin-2 during development leads to reduced trabeculation, cytoskeletal disarray, and cardiac wall rupture (Grossmann et al., 2004), suggesting that plakophilin-2 plays a fundamental role in maintaining the structural integrity of cardiomyocytes. In the clinical setting, most *PKP2* mutations identified in patients with AC are heterozygous and lead to late-onset disease (Calkins et al., 2017; Ohno et al., 2013; van Tintelen et al., 2006). This suggests that the haploinsufficiency of *PKP2* gradually affects cardiac function during a substantial latent asymptomatic period. Conversely, patients with compound or digenic heterozygosity of desmosome genes, including *PKP2*, present with a more severe phenotype (Chen et al., 2019; Gandjbakhch et al., 2018; Rigato et al., 2013). In an extremely rare case in humans, homozygous deletion of *PKP2* led to left ventricular non-compaction and patient death at 12 days of age due to severe fetal-onset heart failure (Ramond et al., 2017). For cases with severe clinical phenotypes, restoring gene function may serve as an upstream therapy for the loss of function of *PKP2*. To test this hypothesis, a human cellular model that recapitulates disease pathology and provides distinct readouts for phenotypic analysis is needed. Induced pluripotent stem cell-derived cardiomyocytes (iPSC-CMs) carrying *PKP2* mutations exhibit a significant decrease in the levels of plakophilin-2, plakoglobin, and the gap-junction protein connexin 43 (Caspi et al., 2013; Ma et al., 2013). Distorted desmosomes or clusters of lipid droplets were observed in iPSC-CMs. Findings from a study using iPSC-CMs carrying a homozygous frameshift mutation in *PKP2*, revealed that induction of adult-like metabolism by treatment with adipogenic stimuli exaggerates lipogenesis and apoptosis in iPSC-CMs (Kim et al., 2013). Although these studies demonstrate the pathological role of mutations in *PKP2* in AC iPSC-CMs, the use of control iPSCs derived from healthy individuals cannot completely exclude the influence of different genetic backgrounds. Moreover, how the human *PKP2* mutation affects contractile function in a differentiated monolayer of iPSC-CMs remains unknown.

In this study, we established iPSCs from a patient with AC carrying a heterozygous frameshift *PKP2* mutation and generated an isogenic set of iPSC clones harboring three genotypes (heterozygous mutation, homozygous corrected, and homozygous mutation) using CRISPR/Cas9 genome editing. The arrangement of the haplotype of *PKP2* alleles led to a dose adjustment of protein expression in the isogenic set of iPSC-CMs and demonstrated that plakophilin-2 deficiency led to reduced contractility, disrupted intercalated disc structures, and impaired desmosome assembly in iPSC-CMs. We further generated isogenic lines in which allele-specific fluorescent labeling of endogenous *DSG2* allowed real-time imaging of desmosome assembly under an adjusted dose of plakophilin-2. Adeno-associated virus (AAV)-mediated gene replacement of *PKP2* recovered contractility, and desmosome dynamics during the recovery phase were sequentially captured through desmosome imaging in plakophilin-2-deficient iPSC-CMs. The isogenic set of iPSC-CMs with adjusted levels of *PKP2* expression recapitulates reduced contractility and impaired desmosome assembly and provides a useful cellular model for phenotypic analysis and the development of therapeutics.

## Results

### Generation of iPSCs from a patient with AC harboring a heterozygous frameshift mutation in *PKP2* and their differentiation to cardiomyocytes

**Figure 1:**
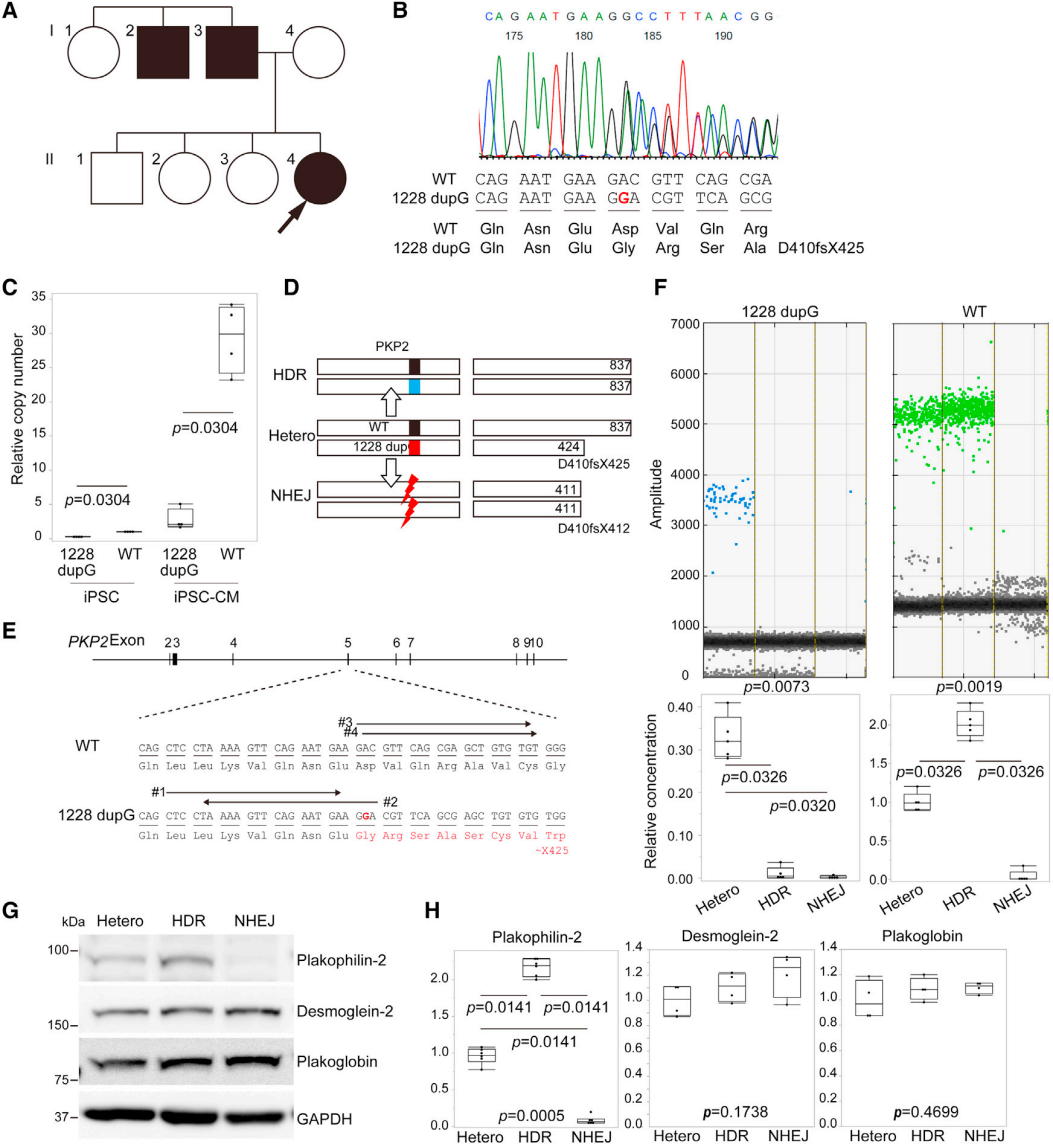
Generation of isogenic iPSCs and differentiation to cardiomyocytes (A) Family pedigree chart of the proband. Cases who presented with ventricular arrhythmia are shown as black circles (females) or black boxes (males). The proband is indicated by an arrow. The proband's father was diagnosed with AC with a *PKP2* mutation. (B) Direct Sanger sequence analysis using genomic DNA extracted from the peripheral blood of the patient. (C) Relative copy number of *PKP2* transcripts in iPSCs and iPSC-CMs was calculated and normalized to that of TATA binding protein (*TBP*) transcripts in each sample. Relative copy number was calculated as the ratio normalized to the levels of WT transcripts in iPSCs (Mann-Whitney test, four independent experiments). (D) Scheme for generating isogenic iPSCs and the predicted length of plakophilin-2 protein in isogenic iPSC clones. (E) The targeted site of genome editing around the 1228 dupG mutation in exon 5 of human *PKP2*. gRNA #1 used a mutant AGG sequence as the PAM sequence, and gRNA #2 contained a 20-bp sequence corresponding to the mutant sequence at its 5′ region. gRNA #3 and #4 target the downstream sequence of 1228 dupG. (F) Representative positive droplet signals from ddPCR analysis are shown in the top. The concentration (copies/l) of each *PKP2* transcript in the cDNA samples was normalized to that of the *TBP* transcript. Relative copy number was calculated as the ratio normalized to the value of the WT transcript in Hetero-iPSCs (Kruskal-Wallis test followed by Steel-Dwass test, five independent experiments). (G) Whole-cell lysates were extracted from each iPSC clone and analyzed using western blotting with the indicated antibodies. (H) Quantification of protein expression normalized to GAPDH expression (Kruskal-Wallis test followed by Steel-Dwass test, four to six independent experiments).

We encountered a 19-year-old female patient diagnosed with AC according to the three major criteria (Marcus et al., 2010), namely repolarization abnormalities in the electrocardiogram, lethal arrhythmias, and family history (Figure 1A), with pathological mutations in *PKP2* (c.1228 dupG, p.D410fsX425,; Figure 1B). Echocardiography revealed that ejection fraction and left-ventricular diameter were within normal limits; however, right-ventricular diameter was slightly dilated (Figure S1A). We screened 404 genes related to inherited cardiovascular disease and confirmed that no deleterious mutations were present in other desmosomal genes, including *DSC2*, *DSG2*, *JUP*, and *DSP*, involved in the etiology of AC. We generated iPSCs from patient-derived peripheral blood mononuclear cells (PBMCs). The generated iPSCs were positive for SSEA4, TRA-1-60, OCT3/4, and NANOG (Figure S1B), negative for Sendai virus-mediated transgenes (Figure S1C), and had a normal karyotype (Figure S1D) and tri-lineage differentiation capacity (Figure S1E). Levels of plakophilin-2 expression were lower in patient-derived iPSCs than in iPSCs generated from a healthy control (Figure S1F). We differentiated the iPSCs into cardiomyocytes according to the chemically defined monolayer protocol, as described (Burridge et al., 2014), which yielded approximately 80%–90% troponin T-positive cardiomyocytes at day 10 after induction of differentiation (Figure S1G). To evaluate the levels of transcript expression from each allele in iPSCs and iPSC-CMs, PCR probes that specifically detect wild-type (WT) and 1228 dupG transcript were used (Figure S1H). For inter-sample comparison of *PKP2* expression between iPSCs and iPSC-CMs, levels of TATA binding protein (*TBP*) were used as internal control (Figure S1I). Results from droplet digital PCR (ddPCR) analysis using cDNA obtained from patient-derived iPSCs and iPSC-CMs revealed that the copy number of the 1228 dupG transcript was 27% lower than that of the WT transcript, and the relative copy number of WT transcripts in iPSC-CMs at day 10 increased approximately 30-fold compared with undifferentiated iPSCs (Figure 1C). The copy number of *PKP2* 1228 dupG transcripts remained low, whereas the copy number of WT *PKP2* transcripts was approximately 10-fold higher than that of the 1228 dupG transcripts in iPSC-CMs. These data suggest that *PKP2* transcripts containing 1228 dupG were unstable and that the difference in the absolute level of transcripts between WT and 1228 dupG increased in differentiated cardiomyocytes.

### Generating a set of isogenic iPSCs using CRISPR/Cas9 genome editing

We generated iPSC clones with the same genetic background and precisely modified genotypes to correct the dosage of *PKP2* transcripts through homology-directed repair (HDR) using CRISPR/Cas9 genome editing. We also aimed to generate the isogenic model expected to exhibit distinct phenotype by introducing a homozygous frameshift mutation through non-homologous end-joining (NHEJ) in *PKP2* of patient-derived iPSCs (Figure 1D). We designed two mutant allele-specific gRNAs (#1 and #2) and common gRNAs (#3 and #4), which targeted both the WT and mutant alleles (Figure 1E). We validated their cleavage ability using the single-strand annealing assay (Mashiko et al., 2013), Cel-I assay, and Sanger sequencing (Figures S1J, S1K, and S1L), and we selected gRNA #1 and #4 for further experiments to induce HDR and NHEJ, respectively. The repair template vector was constructed to replace the mutant sequence with HDR (Figure S1M). After several rounds of sib selection, we obtained an iPSC clone containing homozygous WT alleles (HDR-iPSC) and an iPSC clone containing homozygous frameshift alleles (NHEJ-iPSC). We also obtained a control iPSC clone in which the heterozygous frameshift mutation at *PKP2* remained intact during the same sib selection procedure (Hetero-iPSC) (Figure S1N). The NHEJ clone harbored 31 bp deletions (Δ1230–1260) in both alleles, which were expected to produce truncated plakophilin-2 containing 411 amino acids (Figure 1D). The iPSC clones had uniformly round colonies (Figure S2A), expressed pluripotent markers (Figure S2B), exhibited normal karyotypes (Figure S2C), and had an identical genetic background compared with the patient, as evaluated by short tandem repeat analysis (Figure S2D). Quantitative real-time PCR analysis using a common probe targeting both transcripts (Figure S1H) revealed that relative mRNA expression of *PKP2* was recovered from Hetero- to HDR-iPSCs, and reduced amounts of *PKP2* mRNA, with homozygous frameshift mutations, were transcribed in NHEJ-iPSCs (Figure S2E). ddPCR analysis using a specific probe revealed that mutant transcripts from the 1228 dupG allele were completely abolished in HDR- and NHEJ-iPSCs, and transcripts from the WT allele were recovered in Hetero-to HDR-iPSCs with a 2-fold increase in levels (Figure 1F). Western blot analysis revealed that plakophilin-2 expression was abolished in NHEJ-iPSCs and recovered in HDR-iPSCs (Figures 1G and 1H). Plakoglobin and desmoglein-2, which are encoded by *JUP* and *DSG2*, respectively, are the major components of desmosomes. The expression levels of these proteins are decreased in iPSC-CMs (Caspi et al., 2013; Ma et al., 2013) and myocardium (Rasmussen et al., 2014) in patients with AC with mutations in *PKP2*. In an undifferentiated state, neither protein expression levels nor cellular localization of these junctional proteins was affected in the isogenic iPSCs (Figures 1G, 1H, and S2F). The efficiencies of differentiation, evaluated using fluorescence-activated cell sorting (FACS) using anti-troponin T antibody, were comparable between these cells, and >80% were identified as differentiated without purification (Figure S2G). Truncated plakophilin-2 protein transcribed from the mutant *PKP2* locus containing a frameshift mutation was not detected in either Hetero- or NHEJ-iPSC-CMs (Figure S2H).

### Plakophilin-2 insufficiency decreases the contractility of the differentiated monolayer iPSC-CMs

**Figure 2:**
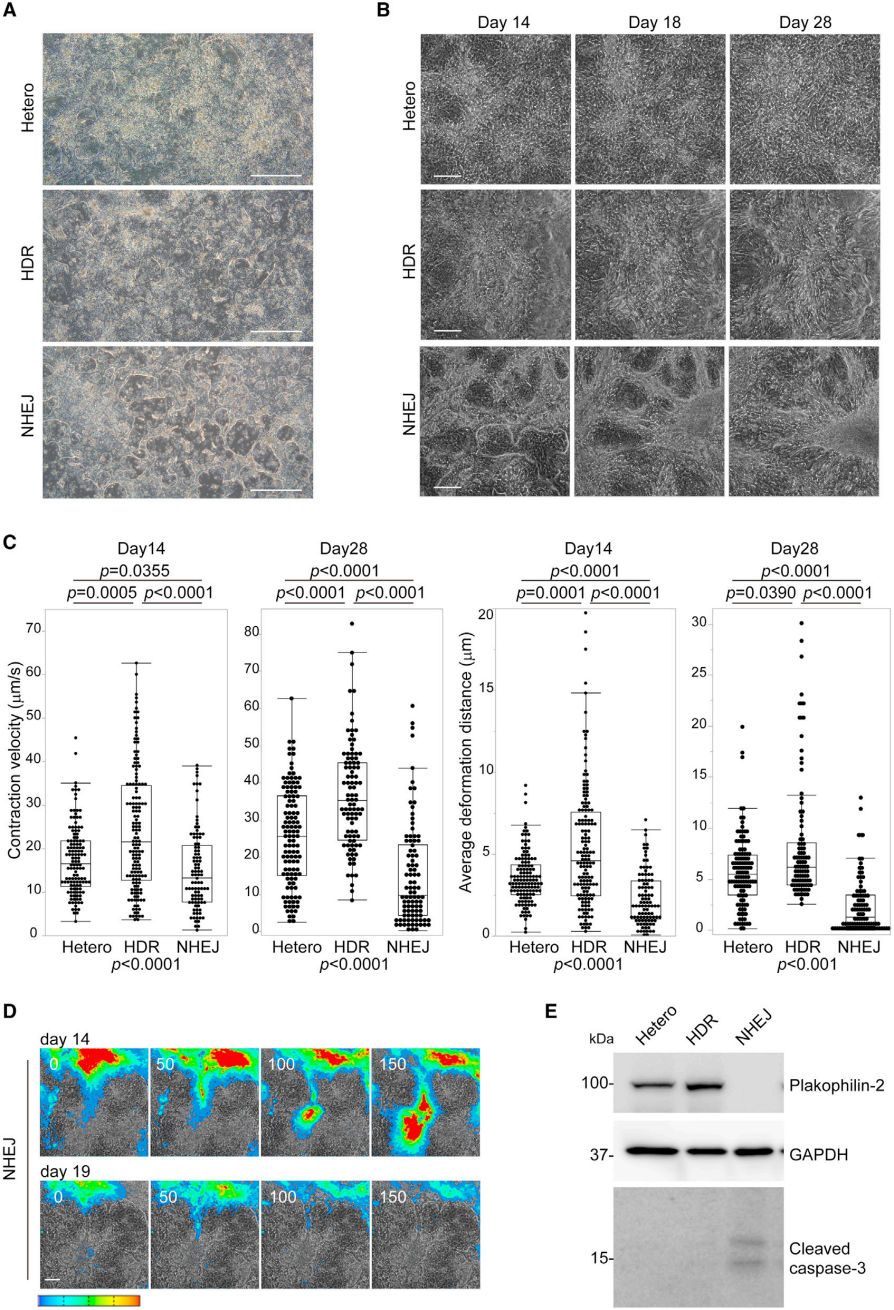
Plakophilin-2 insufficiency decreases the contractility of the differentiated monolayer iPSC-CMs (A) Bright-field image of the monolayer Hetero-, HDR-, or NHEJ-iPSC-CMs at day 10 after induction of differentiation. Scale bar: 1 mm. (B) Sequential observation of the monolayer of iPSC-CMs using motion vector analysis. Bright-field images of fixed positions at specific coordinates on days 14, 18, and 28 are shown. Scale bar: 200 μm. (C) Contraction velocity (CV) and deformation distance (DD) in HDR- and NHEJ-iPSC-CMs on days 14 and 28 were analyzed using motion vector analysis (Kruskal-Wallis test followed by Steel-Dwass test). Number of analyzed regions of interest (ROI) for Hetero: 129, HDR: 148, and NHEJ: 96 on day 14. Number of analyzed ROI for Hetero: 117, HDR: 94, NHEJ: 94 on day 28. Data were collected from three independent experiments. (D) Label-free detection of excitation propagation using motion vector analysis excitation. Excitation propagation through the oriented fiber structure was sequentially observed in NHEJ-iPSC-CMs on days 14 and 19. Serial consecutive fluorescence images obtained every 50 ms are shown. Scale bar: 200 μm. Color range from blue to red represents motion velocity from 0 to 30 μm/s, respectively. (E) Whole-cell lysates were extracted from Hetero-, HDR-, and NHEJ-iPSC-CMs at 28 days after differentiation and analyzed using western blotting with the indicated antibodies.

The monolayer differentiation protocol using chemically defined medium produces contractile sheets of cardiomyocytes from iPSCs within 14 days and provides a rapid and convenient platform for functional analysis (Burridge et al., 2014; Sharma et al., 2018). Although reduced contractility and arrhythmogenicity are the major diagnostic criteria of AC (Marcus et al., 2010), how *PKP2* mutation affects the contractility of a differentiated monolayer of iPSC-CMs is unknown. To investigate cell morphology and contractility, differentiated isogenic iPSC-CMs were sequentially evaluated as monolayer cardiomyocytes. Differentiated cardiomyocytes as a monolayer in a chemically defined medium are not suited for long-term adhesion and occasionally detach from the surface (Burridge et al., 2014). We incubated iPSC-CMs without lactate purification and exchanged the medium with serum-containing medium on day 14. This promoted the proliferation of non-cardiomyocytes. However, it allowed us to continuously observe the same culture plate (without the need to replate) and evaluate the kinetic properties of cultured iPSC-CMs in real time using contraction velocity (CV) and deformation distance (DD), representing contractile function and contractile force, respectively, defined using motion vector analysis (Hayakawa et al., 2014; Ito et al., 2019; Wu et al., 2019). From days 8–10, the connected layer structure and coordinated dynamic contraction were similarly observed in both Hetero- and HDR-iPSC-CMs. In NHEJ-iPSC-CMs, hole-like defects, which gradually increased from days 8 to 10, appeared in the connecting cardiomyocytes (Figure 2A), suggesting fragile cell-cell adhesions under increased contractile tension. NHEJ-iPSC-CMs exhibited net-like structures around day 14 and progressive contractile dysfunction over time from days 14 to 28 (Figures 2B, 2C and Video S1). NHEJ-iPSC-CMs exhibited progressive conduction disturbances, as evaluated by color maps converted from motion amplitude (Figure 2D and Video S2), but these parameters were comparable between Hetero- and HDR-iPSC-CMs (Figure S3A). By contrast, Hetero-iPSC-CMs, with a one-half dose of plakophilin-2, did not exhibit apparent morphological differences compared with HDR-iPSC-CMs. However, sequential observation using motion vector analysis clarified that CV and DD were significantly decreased in Hetero-iPSC-CMs compared with the levels in HDR-iPSC-CMs at both day 14 and day 28 after differentiation (Figure 2, Videos S3 and S4). Motion analysis under continuous pacing confirmed the decreased contractility in Hetero- and NHEJ-iPSC-CMs both on day 14 and on day 28 after differentiation (Figure S3B). Increased levels of apoptosis have been observed in iPSC-CMs carrying *PKP2* mutations (Caspi et al., 2013; Kim et al., 2013). Under our experimental conditions, cleaved caspase-3, an apoptosis marker, was detected in NHEJ-iPSC-CMs, and not Hetero- or HDR-iPSC-CMs, 28 days after differentiation (Figure 2). FACS analysis on days 14 and 28 after differentiation demonstrated that the proportion of troponin T-positive cardiomyocytes was gradually decreased in NHEJ-iPSC-CMs compared with Hetero- or HDR-iPSC-CMs (Figure S3C), suggesting the myocyte loss under continuous tension in plakophilin-2-deficient iPSC-CMs. These data suggest that the monolayer differentiation protocol revealed a distinct phenotype in NHEJ-iPSC-CMs and elicited reduced contractility as a pathological phenotype caused by plakophilin-2 haploinsufficiency in Hetero-iPSC-CMs in a short period of time.

Video S1. Sequential observation of the monolayer iPSC-CMs using motion vector analysis Bright field images of the fixed positions at specific coordinates of NHEJ-iPSC-CMs at day 14, 18 and 28 are shown.

Video S2. Sequential observation of the monolayer of NHEJ-iPSC-CMs using motion vector analysis Color maps representing excitation propagation (1/4 speed) of the fixed 1 positions at specific coordinates of NHEJ-iPSC-CMs at days 14 and 19 are shown. At day 14, continuous downward excitation propagation was observed i NHEJ-iPSC-CMs. At day 19, intermittent excitation propagation (2:1 conduction block) in the same fiber structure was observed.

Video S3. Sequential observation of the monolayer iPSC-CMs using motion vector analysis

Video S4. Sequential observation of the monolayer iPSC-CMs using motion vector analysis Bright field images of the fixed positions at specific coordinates of Hetero- (Video 3) and HDR- (Video 4) iPSC-CMs at day 14, 18 and 28 are shown.

### Plakophilin-2 insufficiency disrupts intercalated disc structure

**Figure 3:**
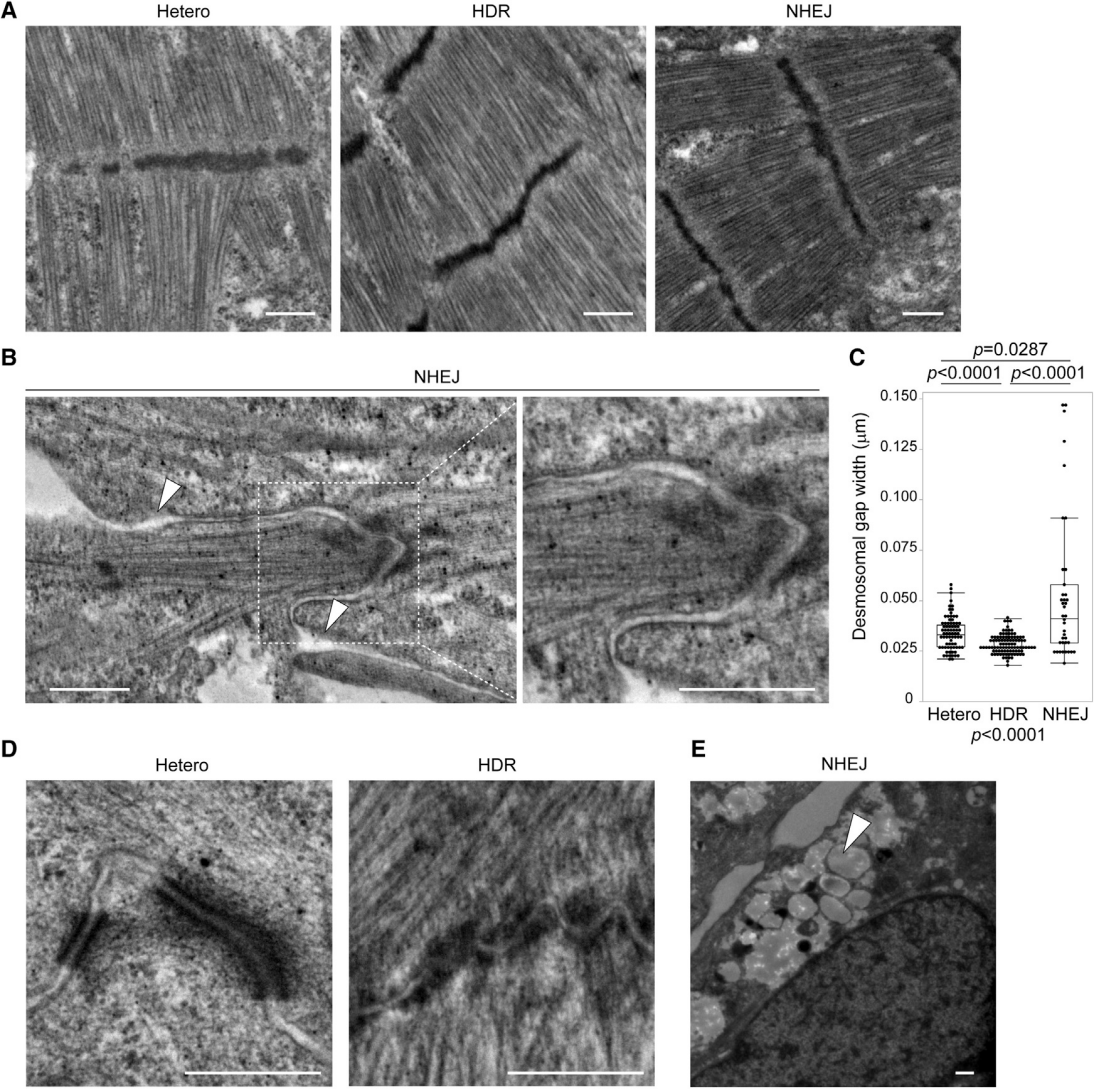
Plakophilin-2 insufficiency disrupts intercalated disc structures (A) Sarcomere structure in isogenic iPSC-CMs 28 days after differentiation was observed using transmission electron microscopy (TEM). Scale bar: 500 nm. (B) Representative desmosome structures and dissociated intercalated discs (arrowheads) in NHEJ-iPSC CMs are shown. The area enclosed within the white dotted square is enlarged on the right. Scale bar: 500 nm. (C) Desmosomal gap width was calculated using TEM images by a blinded operator (Kruskal-Wallis test followed by Steel-Dwass test). Number of analyzed regions in Hetero-iPSC-CMs: 76, HDR-iPSC-CMs: 95, NHEJ-iPSC-CMs: 39. Data were collected from three independent experiments. (D) Representative desmosome structures in Hetero- and HDR-iPSC-CMs. Scale bar: 500 nm. (E) Cytosolic accumulation of lipid droplets in NHEJ-iPSC-CMs (arrowhead). Scale bar: 500 nm.

Reduced contractility in Hetero-iPSC-CMs within 28 days after monolayer differentiation indicates a damaged microstructure caused by insufficient plakophilin-2. To evaluate differences in subcellular morphology, we fixed isogenic iPSC-CMs 28 days after differentiation and observed them using transmission electron microscopy (TEM). Sarcomere structures with Z-lines were observed in isogenic iPSC-CMs (Figure 3A). NHEJ-iPSC-CMs exhibited significantly increased desmosome gap lengths and severely disrupted intercalated disc structures (Figures 3B and 3C). By contrast, significant morphological abnormalities in desmosomes were not observed in Hetero-iPSC-CMs compared with HDR-iPSC-CMs (Figure 3D). Quantitative analysis targeting desmosomes, represented as electron-dense areas, revealed that, in Hetero-iPSC-CMs, desmosomal gap width was significantly increased compared with HDR-iPSC-CMs (Figure 3C). A TEM study using beating embryoid bodies 40 days after differentiation demonstrated widened and distorted desmosomes in iPSC-CMs with heterozygous *PKP2* frameshift mutations compared with iPSC-CMs generated from healthy controls (Caspi et al., 2013). Our data suggest that microstructural abnormalities were produced in iPSC-CMs at a relatively early phase after differentiation, probably because of the stronger contractile tension promoted by the monolayer differentiation protocol. Studies have reported abnormal lipid accumulation in iPSC-CMs with *PKP2* mutations after differentiation or after additional adipogenic stimulation (Caspi et al., 2013; Kim et al., 2013; Ma et al., 2013). Under our experimental conditions, significant cytosolic lipid droplets were observed in NHEJ-iPSC-CMs 28 days after differentiation (Figure 3E), whereas lipid accumulation was not remarkable in either Hetero- or HDR-iPSC-CMs.

### Plakophilin-2 haploinsufficiency impairs desmosome assembly in iPSC-CMs

**Figure 4:**
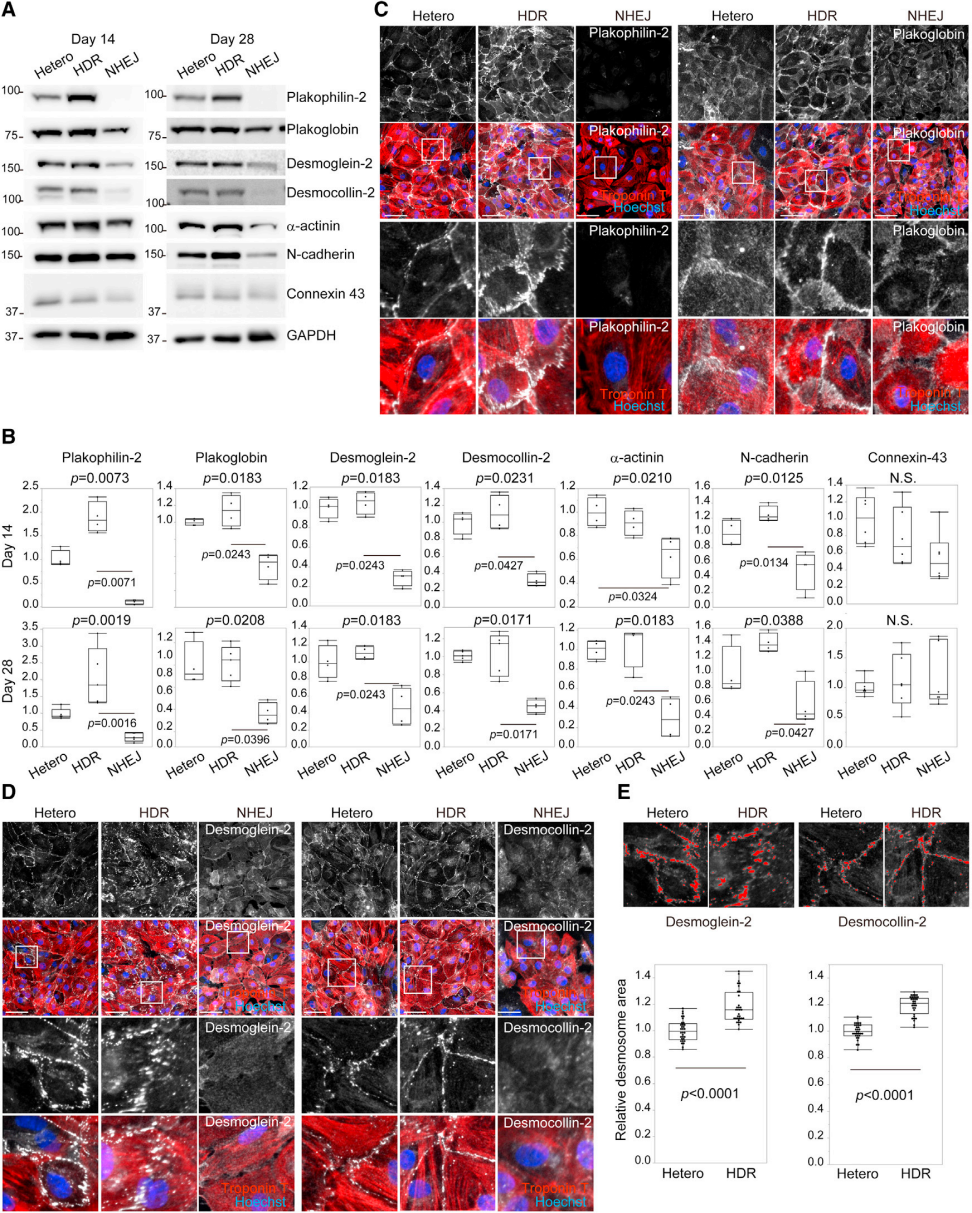
Plakophilin-2 haploinsufficiency leads to impaired desmosome assembly in iPSC-CMs (A) Whole-cell lysates were extracted from Hetero-, HDR-, and NHEJ-iPSC-CMs on days 14 and 28 after differentiation and analyzed by western blotting using the indicated antibodies. (B) Quantified protein expression levels normalized by GAPDH expression are shown (Kruskal-Wallis test followed by Dunn's test, four to six independent experiments). (C and D)Hetero-, HDR-, and NHEJ-iPSC-CMs were replated on 96-well plates at day 10 after differentiation and subsequently fixed and immunostained at day 14 with the indicated antibodies. Scale bar: 50 μm. Areas enclosed within white squares are enlarged at the bottom. (E) The images shown in (D) were quantitatively analyzed using high-content imaging. Top: raw immunostained images and captured intensity images detected using high-content imaging. Relative desmosome area of each fluorescent signal in HDR-iPSC-CMs was normalized to that in Hetero-iPSC-CMs (Mann-Whitney test, n = 32 images in each iPSC-CM from four independent experiments).

The monolayer differentiation protocol elicited decreased contraction and disrupted intercalated disc structures caused by plakophilin-2 haploinsufficiency. To identify the initial molecular processes involved, we evaluated the localization and expression levels of desmosomal proteins in isogenic iPSC-CMs using immunostaining and western blotting. At the cell-cell junction, plakophilin-2 expression was recovered in Hetero-to HDR-iPSC-CMs and abolished in NHEJ-iPSC-CMs (Figures 4A, 4B, and 4C). Plakoglobin, an anchoring protein that connects desmosomal cadherins to desmoplakin, is found in both desmosomes and *fascia adherens* (Sheikh et al., 2009). Desmoglein-2 and desmocollin-2 are desmosomal cadherins that form homo- and heteropolymers in intercellular spaces; their C-terminal tails are located in the cytoplasm and are connected to plakophilin-2 (Al-Jassar et al., 2013). Levels of plakoglobin, desmoglein-2, and desmocollin-2 expression were significantly decreased in NHEJ-iPSC-CMs. Plakoglobin remained localized at the cell-cell junctions (Figure 4C), whereas desmoglein-2 and desmocollin-2 were completely dislodged from the cellular periphery and were diffusely distributed in the cytosol in NHEJ-iPSC-CMs (Figure 4D). By contrast, expression levels or cellular localization of desmosomal proteins was not significantly affected in Hetero-iPSC-CMs compared with HDR-iPSC-CMs (Figures 4A, 4B, and 4D), which is consistent with findings that both abundance and localization of intercalated disc proteins are unaffected by plakophilin-2 haploinsufficiency in murine hearts (Cerrone et al., 2012). Peripheral localization of desmoplakin was decreased in NHEJ-iPSC-CMs but was not significantly affected in Hetero-iPSC-CMs (Figure S4A). *Fascia adherens* junctions, which span the extracellular space and link cytoskeletal actin filaments with junction complexes, include transmembrane proteins that are mainly composed of N-cadherin (Lyon et al., 2015). Immunostaining and western blot analysis revealed that N-cadherin expression was significantly decreased in NHEJ-iPSC-CMs but was not significantly different between Hetero- and HDR-iPSC-CMs (Figures 4A, 4B, and S4A). Localization of α-actinin, cytoskeletal actin, or vinculin, which make up the *fascia adherens* network, or expression levels of connexin 43 were not significantly affected in both Hetero- and NHEJ-iPSC-CMs (Figures 4A, 4B, and S4B). Desmoglein-2, desmocollin-2, and desmoplakin were expressed at the cell-cell junctions with punctate distribution in both Hetero- and HDR-iPSC-CMs. To evaluate the effect of plakophilin-2 haploinsufficiency on desmosomal cadherins and desmoplakin, we performed high-content imaging (Ishizu et al., 2017) for quantifying the area of desmosome distribution and found that it was significantly decreased in Hetero-iPSC-CMs compared with HDR-iPSC-CMs (Figures 4E and S4C).These data suggest that the loss of plakophilin-2 affected the stability of intercalated disc proteins, and plakophilin-2 haploinsufficiency did not affect the expression or localization of desmosomal proteins but decreased the area of desmosomes, with punctate distribution after monolayer differentiation.

### Allele-specific fluorescent labeling of *DSG2* captures desmosome dynamics in isogenic iPSC-CMs

**Figure 5:**
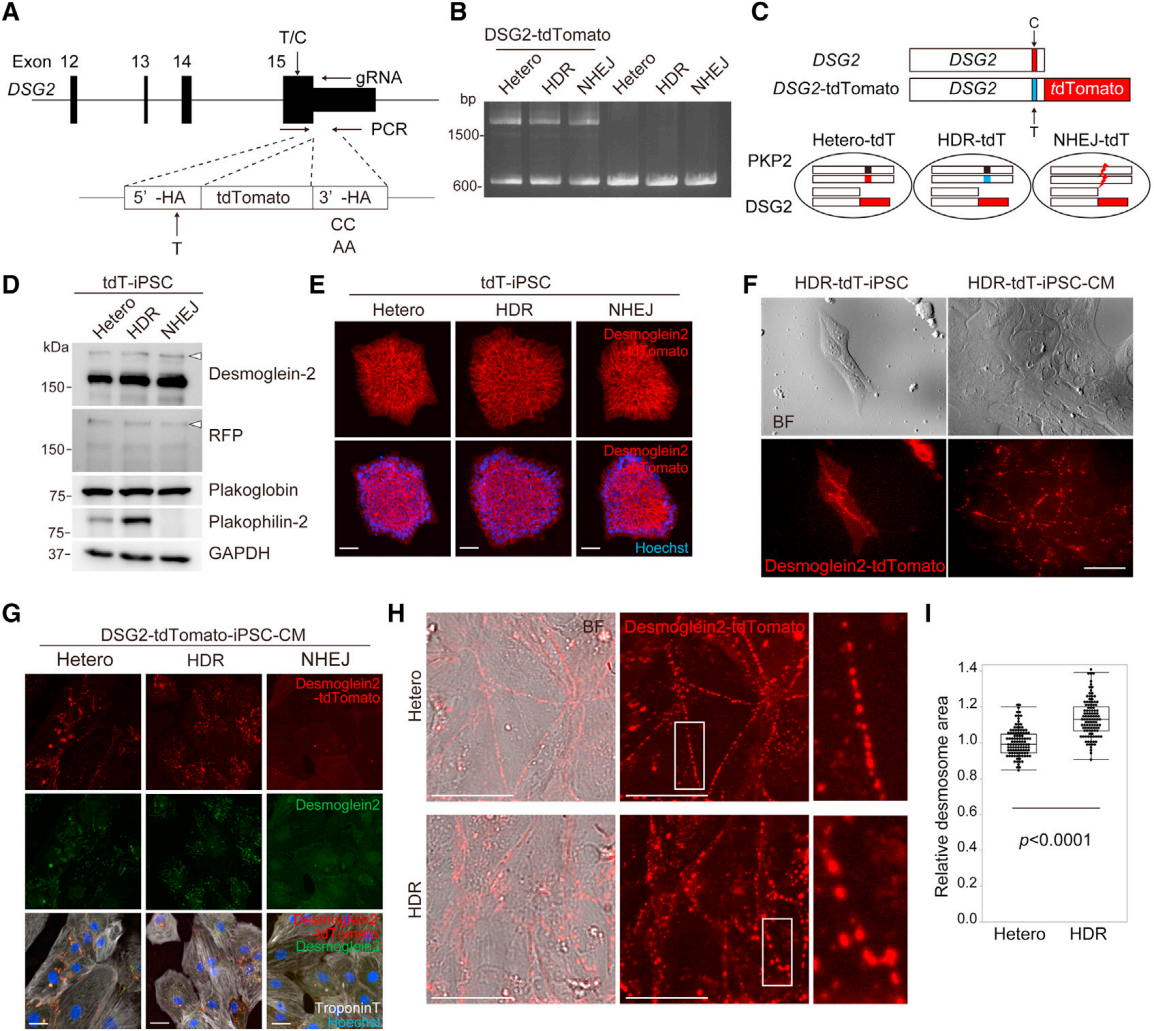
Allele-specific fluorescent labeling of *DSG2* captures desmosome dynamics in isogenic iPSC-CMs (A) The targeted site of genome editing around the 3′ terminus of exon 15 of human *DSG2*. The repair template DNA contained T at the SNP site in the 5′-homology arm to distinguish the knocked-in allele after genome editing. The 3′-homology arm contained PAM sequence modification from CC to AA to avoid recleavage by Cas9. Arrows indicate the positions of the PCR primers used to distinguish the knocked-in allele. (B) Electrophoresis of PCR products using genomic DNA extracted from Hetero-, HDR-, NHEJ-tdT-iPSCs, or original isogenic iPSCs. PCR products at 2,277 bp were derived from the knocked-in allele, and those at 632 bp were derived from the non-edited allele. (C) Scheme showing isogenic iPSCs containing the identical set of *DSG2* alleles in which tdTomato was introduced specifically into the SNP: T allele; the other SNP: C allele remained intact. (D) Whole-cell lysates were extracted from each iPSC line and analyzed using western blotting with the indicated antibodies. Arrowheads indicate the desmoglein-2-tdTomato fusion protein. (E) Isogenic tdT-iPSCs were fixed, and nuclei were stained with Hoechst stain. Scale bar: 50 μm. (F) Live-cell images (bright-field and fluorescence images) of HDR-tdT-iPSCs and HDR-tdT-iPSC-CMs on day 14 after differentiation. Scale bar: 50 μm. (G) Isogenic tdT-iPSCs were fixed and immunostained with anti-troponin T and desmoglein-2 antibodies. Nuclei were stained with Hoechst stain. Scale bar: 50 μm. (H) Live-cell images of Hetero- and HDR-tdT-iPSC-CMs 14 days after differentiation were obtained using high-content imaging. Areas enclosed within white squares in the middle are enlarged in at the right. Scale bars: 50 μm. (I) The images shown in (H) were quantitatively analyzed using high-content imaging. The relative desmosome area of each fluorescent signal in HDR-tdT-iPSC-CMs was normalized to that in Hetero-tdT-iPSC-CMs (Mann-Whitney test, n = 108 images in each iPSC-CM from four independent experiments).

We recently reported that the loss of desmoglein-2 in human iPSCs does not affect the differentiation process or cell morphology in iPSC-CMs (Shiba et al., 2021). On the basis of these findings, we speculated that fluorescent labeling of endogenous desmoglein-2 could be a useful marker for assessing the degree of deterioration or restoration of desmosomes affected by the insufficient expression of plakophilin-2 in cardiomyocytes. To establish a model for desmosome-imaging, the tdTomato fluorescent reporter was knocked-in at the 3′ terminus of *DSG2* in the three established isogenic iPSCs using genome editing. Because a synonymous single-nucleotide mutation (SNP: T > C) was identified just upstream of the stop codon of *DSG2*, we designed the repair template DNA containing T at the SNP site in the 5′-homology arm to distinguish the knocked-in allele after genome editing (Figure 5A). After repeated rounds of sib selection, followed by PCR and Sanger sequencing (Figures 5B and S5A), we established an isogenic set of iPSCs containing an identical set of *DSG2* alleles in which tdTomato was introduced specifically into SNP: T allele, whereas SNP: C allele remained intact (named Hetero-tdT-, HDR-tdT-, and NHEJ-tdT-iPSC; Figure 5C). All the isogenic tdT-iPSCs generated showed a normal karyotype and were positive for pluripotent markers (Figures S5B and S5C). ddPCR analysis using a specific probe targeting SNP: T or C revealed that *DSG2-tdTomato* transcripts were similarly expressed in isogenic iPSCs at a median rate of 62.5%–65.8% compared with WT *DSG2* transcripts (Figure S5D). Western blotting and immunofluorescence staining demonstrated that the desmoglein-2-tdTomato fusion protein was similarly expressed in isogenic iPSCs (Figures 5D and 5E). Desmoglein-2-tdTomato initially localized at the cell periphery, gradually assembled after differentiation, and then exhibited punctate distribution (Figure 5F). These tdTomato signals were merged with the immunofluorescence signals detected by the anti-desmoglein-2 antibody (Figure S5E). Cellular localization of desmoglein-2-tdTomato fusion protein was similar between Hetero-tdT- and HDR-tdT-iPSC-CMs and not detected in NHEJ-tdT-iPSCMs (Figure 5G). Quantitative analysis using high-content imaging revealed that the desmosome area represented by tdTomato fluorescence in live cells was significantly smaller in Hetero-tdT-iPSC-CMs than in HDR-tdT-iPSC-CMs (Figures 5H and 5I), suggesting that our established isogenic knockin model captured impaired desmosome assembly caused by plakophilin-2 haploinsufficiency in live iPSC-CMs.

### AAV-mediated gene delivery of *PKP2* recovered contractility and desmosome assembly in plakophilin-2-deficient iPSC-CMs

**Figure 6:**
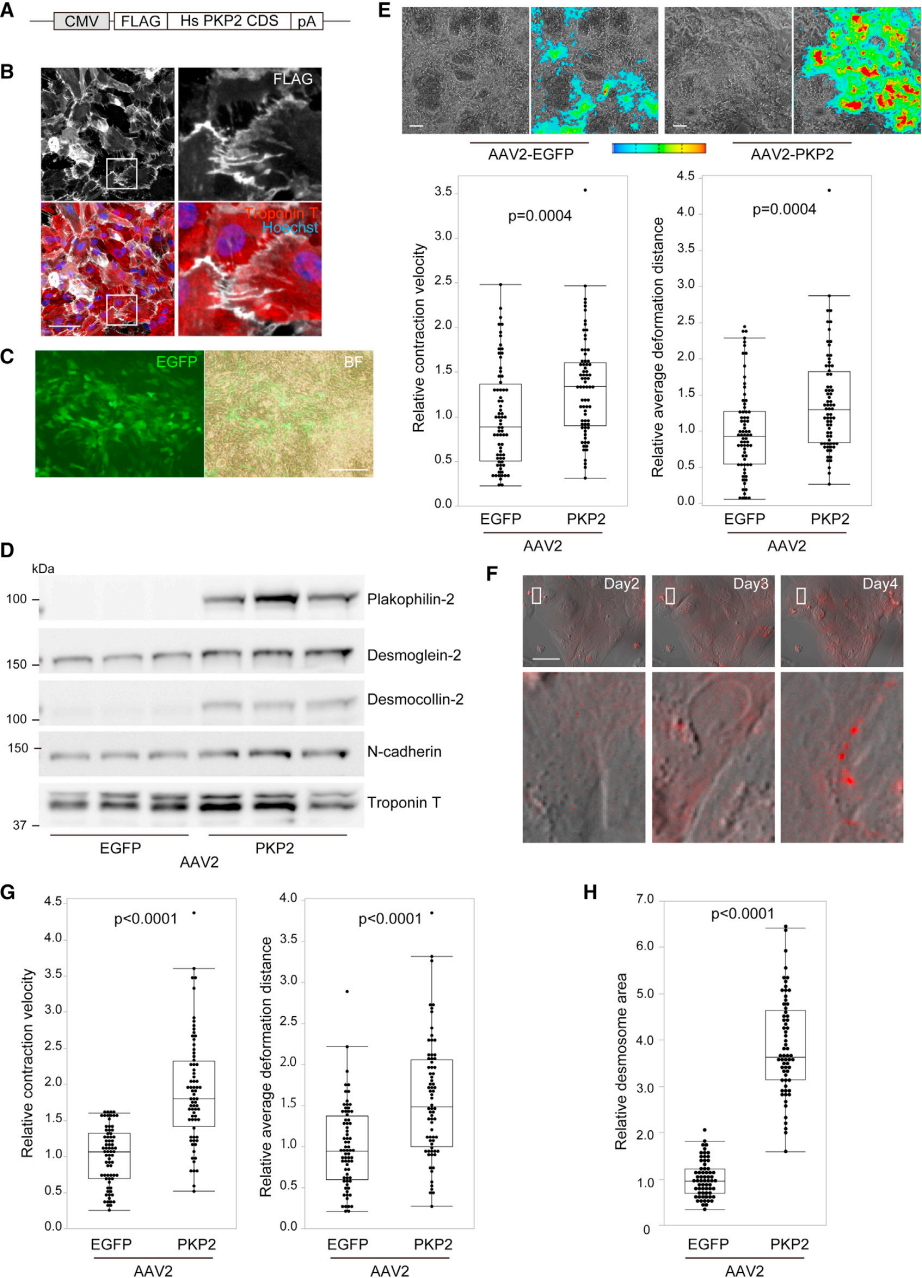
AAV-mediated gene delivery of *PKP2* recovered contractility and desmosome assembly in plakophilin-2-deficient iPSC-CMs (A) N-terminal FLAG-tagged full-length human *PKP2* coding sequence (Hs *PKP2* CDS) was subcloned into the expression vector with a CMV promoter and poly(A) sequence to generate the AAV2 vector. (B) NHEJ-iPSC-CMs transduced with AAV2 encoding FLAG-tagged *PKP2* were fixed and immunostained with the indicated antibodies 5 days after transduction. Areas enclosed within white squares in the left are enlarged in the panels on the right. Scale bar: 50 μm. (C) Monolayer contracting NHEJ-iPSC-CMs cultured in 12-well plates at day 10 after differentiation were transduced with approximately 1.0 × 10^4^ vg/cell of AAV2-*EGFP* or AAV2-*PKP2*. Fourteen days after transduction, EGFP expression in contracting NHEJ-iPSC-CMs was observed through fluorescence microscopy. Scale bar: 200 μm. (D) NHEJ-iPSC-CMs were treated as described in (C). Whole-cell lysates were extracted from iPSC-CMs 14 days after transduction and analyzed by western blotting using the indicated antibodies. (E) Bright-field images and excitation propagation were detected by motion vectors in NHEJ-iPSC-CMs 14 days after transduction, either with AAV2-*EGFP* or with AAV2-*PKP2*. Color range from blue to red represents motion velocity from 0 to 30 μm/s, respectively. Scale bar: 100 μm. CV and DD in iPSC-CMs were calculated using motion vector analysis (Mann-Whitney test, number of analyzed ROIs, AAV2-*EGFP*: 72, AAV2-*PKP2*: 72). Data were collected from three independent experiments. (F) Sequential merged images of bright-field and tdTomato fluorescence after AAV2-*PKP2* transduction in NHEJ-tdT-iPSC-CMs. Scale bar: 50 μm. (G) Hetero-iPSC-CMs were treated as described (C). CV and DD in iPSC-CMs were calculated using motion vector analysis (Mann-Whitney test, number of analyzed ROIs, AAV2-*EGFP*: 72, AAV2-*PKP2*: 72). Data were collected from three independent experiments. (H) Hetero-tdT-iPSC-CMs at day 10 after differentiation were replated and transduced with AAV2-*EGFP* or AAV2-*PKP2*. Fourteen days after transduction; desmosome area was assessed using live-cell high-content imaging. Relative desmosome area of each fluorescence signal in Hetero-iPSC-CMs transduced with AAV2-*PKP2* were normalized to those with AAV2-*EGFP* (Mann-Whitney test, n = 64 images in each iPSC-CM from four independent experiments).

To test proof of concept for gene replacement therapy in human cells, we generated an AAV, containing the N-terminal, FLAG-tagged, full-length human *PKP2* sequence driven by the CMV promoter (AAV2*-PKP2*; Figure 6A). We selected the AAV2 serotype because AAV2 has been shown to efficiently transduce iPSC-CMs (Guan et al., 2015), and the use of AAV2 led to high transduction efficiency in iPSC-CMs under our experimental conditions (Kohama et al., 2020; Shiba et al., 2021) (Figure S6A). FLAG-tagged plakophilin-2 protein localized properly at the cellular periphery in iPSC-CMs (Figure 6B), and AAV2-mediated delivery of plakophilin-2 restored the localization of desmoglein-2, desmocollin-2, and N-cadherin in the cell-cell junctions of NHEJ-iPSC-CMs (Figures S6B and S6C). To evaluate the effect of gene replacement in contracting monolayer cardiomyocytes, AAV2-*PKP2* was transduced into NHEJ-iPSC-CMs at day 10, a time when these cells initially exhibited decreased contractility. Fourteen days after transduction, the transgenes were efficiently introduced into the contracting monolayer of iPSC-CMs (Figure 6C). AAV2-mediated replacement of *PKP2* increased the expression of desmoglein-2, desmocollin-2, and N-cadherin (Figure 6D), prevented the formation of hole-like structures in contracting cardiomyocytes, and restored CV and DD, as evaluated by motion analysis (Figure 6E). Notably, time-lapse imaging using NHEJ-tdT-iPSC-CMs captured the recovery of desmosomes, which gradually assembled at the cell periphery after AAV-mediated *PKP2* replacement (Figure 6F and Video S5). Importantly, transduction of AAV2-*PKP2* recovered CV and DD in Hetero-iPSC-CMs after monolayer differentiation (Figure 6G) and significantly restored desmosome assembly in Hetero-tdT-iPSC-CMs (Figure 6H). These data demonstrate the proof of concept for *PKP2* replacement therapy in human cells and suggest that our established isogenic set of iPSC-CMs is a useful model for providing distinct readouts for therapeutic development.

Video S5. Continuous observation of the restoration process of desmosome assembly in NHEJ-tdT-iPSC-CMs transduced with AAV2-*PKP*2 Images were captured every 30 min from 1 to 4 day after the transduction.

## Discussion

In this study, we generated a set of isogenic iPSCs consisting of three clones with precisely adjusted expression of plakophilin-2. Motion vector analysis after monolayer differentiation and the generation of desmosome-imaging isogenic lines using fluorescence tagging of endogenous *DSG2* recapitulated reduced contractility and impaired desmosome assembly under the adjusted dose of plakophilin-2 within 2–4 weeks after differentiation. Several molecular mechanisms, including cell death, excessive lipogenesis, nuclear translocation of γ-catenin, altered calcium signaling, and altered cellular metabolism, have been shown to cause AC due to mutations in *PKP2* by using human iPSC-CMs (Austin et al., 2019; Caspi et al., 2013; Kim et al., 2013; Ma et al., 2013). However, contractile dysfunction due to *PKP2* mutations has not been fully studied in human iPSC-CMs, although ventricular regional dysfunction is one of the modified Task Force diagnostic criteria for AC (Towbin et al., 2019). Under our experimental conditions, reduced contractility caused by *PKP2* haploinsufficiency was elicited in a short period of time, within 2 weeks after differentiation. Because the monolayer protocol confers strong contraction to iPSC-CMs on culture plates soon after differentiation (Burridge et al., 2014; Gintant et al., 2019), continuous tensile overload may facilitate the disease phenotype among isogenic iPSC-CMs. By contrast, common pathological phenotypes, including cell death and lipid accumulation, were recapitulated in NHEJ-iPSC-CMs, which lack plakophilin-2 expression, but not in Hetero-iPSC-CMs, which have a one-half dose of plakophilin-2, within 4 weeks. Because recapitulation of cell death and lipid accumulation caused by *PKP2* mutation requires long-term culture (∼2 months) to promote maturation of iPSC-CMs (Caspi et al., 2013; Kim et al., 2013; Ma et al., 2013), these phenotypes may not be suitable as readouts for early-phase evaluation.

Loss of plakophilin-2 deficiency led to reduced expression of desmosomal cadherins at the cellular periphery in NHEJ-iPSC-CMs. By contrast, plakophilin-2 haploinsufficiency in Hetero-iPSC-CMs did not significantly affect the expression or localization of desmosomal proteins in Hetero-iPSC-CMs compared with isogenic HDR-iPSC-CMs, which is consistent with findings that heterozygous *Pkp2* knockout mice do not exhibit significant morphological abnormalities or differences in expression of intercalated disc proteins, including connexin 43, N-cadherin, and plakoglobin (Cerrone et al., 2012). In this study, immunostaining with desmoglein-2 or desmocollin-2 and quantitative analysis using high-content imaging revealed that a one-half dose reduction of plakophilin-2 decreased desmosome assembly at the periphery of iPSC-CMs. These data indicate that among outer dense plaque proteins, desmosomal cadherins are the most prone to instability due to decreased plakophilin-2 expression. We recently reported a case of desmoglein-2-deficient cardiomyopathy caused by a rare homozygous stop-gain mutation and established isogenic iPSC-CMs from the patient lacking desmoglein-2 (Shiba et al., 2021). Although the loss of desmoglein-2 expression significantly decreased contractile function in three-dimensional tissues (Li et al., 2020), desmoglein-2 deficiency did not significantly affect the differentiation efficiency or morphology of iPSC-CMs. These data prompted us to choose *DSG2* as a molecular marker for real-time desmosome imaging in our isogenic iPSCs. Although the copy number of mRNA transcribed from the knockin allele was lower than that in the normal allele, the isogenic clones carrying the identical knockin *DSG2* alleles allowed a relative comparison of desmosome dynamics under the adjusted dose of plakophilin-2 expression.

AAV2-mediated gene replacement of *PKP2* restored desmosomal proteins and suppressed contractile dysfunction in both NHEJ- and Hetero-iPSC-CMs. The recovery of desmosomes after AAV2-mediated gene replacement was sequentially captured using the desmosome-imaging isogenic lines. These findings provide proof of the therapeutic concept in human cardiomyocytes but may not be directly applied to clinical settings, as most clinically identified mutations in *PKP2* are heterozygous and disease is late onset (Awad et al., 2008; Calkins et al., 2017; Ohno et al., 2013; van Tintelen et al., 2006). However, compound or digenic heterozygosity of desmosome genes, including *PKP2*, is not rare, and patients with combined mutations present with a more severe phenotype (Chen et al., 2019; Gandjbakhch et al., 2018; Rigato et al., 2013). A recent large-cohort analysis using high-throughput sequence analysis has highlighted the high levels of *PKP2* mutations in patients diagnosed with dilated cardiomyopathy (Haas et al., 2015). These findings suggest that *PKP2* haploinsufficiency may develop into severe biventricular heart failure when combined with other pathogenic mutations or other exogenous environmental factors. Furthermore, homozygous deletion of *PKP2* causes untreatable fetal heart failure with left-ventricular non-compaction (Ramond et al., 2017). Although early-onset disease with severe manifestations is rare, mouse myocardia with *Pkp2* mutations are affected by structural injury due to exercise load or environmental stress (Cruz et al., 2015; van Opbergen et al., 2019). This highlights the need for unconventional therapeutic approaches to prevent disease progression. The isogenic cells that we established represent a human disease model that recapitulates reduced contractility and impaired desmosome assembly and provides a convenient cellular platform for therapeutic screening to test upstream molecular targets.

## Experimental procedures

Details are provided in the supplemental experimental procedures.

### Human samples

The use of patient-derived samples and genomic analysis was approved by the Ethics Committee of Osaka University Hospital, and written informed consent was obtained from all patients. This study conforms to the ethical guidelines for medical and health research involving human participants in Japan and all principles outlined by the Declaration of Helsinki.

### Transfection of plasmids into human iPSCs and selection of targeted clones

Plasmid constructs for genome editing were transfected into iPSCs, and targeted clones were selected as described (Higo et al., 2021; Li et al., 2015).

### Motion vector analysis

As described, cell motion profiles of cardiomyocytes differentiated from iPSCs were acquired using the Cell Motion Imaging System (SI8000, SONY) (Hayakawa et al., 2014; Ito et al., 2019).

### Data and code availability

The data that support the findings of this study are available from the corresponding author upon reasonable request.

## Author contributions

Conceptualization: H.I., S.N., and S.H.; Methodology: H.I., S.N., S.H., L.J., and L.L.; Investigation: H.I., S.N., S.H., M.S., Y.K., T.K., S.K., T.T., S.O., Y.I, S.Y., M.T., and E.I.; Software: T.T.; Writing: original draft, H.I., S.N., and S.H.; Writing:review & editing, S.T., S.M., Y.S., S.Hikoso, and Y.S.; Funding Acquisition: S.H., S.M., S. Hikoso, and Y.S.; Supervision: S.M. and Y.S.

## Conflict of interest

The Department of Medical Therapeutics for Heart Failure is a Joint Research Department with TOA EIYO Pharmaceutical Company.

## Supplemental information

Document S1. Figures S1–S6, Table S1, and Supplemental experimental procedures

Document S2. Article plus supplemental information
