# Supplementary material for: Modeling reduced contractility and impaired desmosome assembly due to plakophilin-2 deficiency using isogenic iPS cell-derived cardiomyocytes
Source: Stem Cell Reports. 2022 Jan 20;17(2):337–51. doi: 10.1016/j.stemcr.2021.12.016 (PMC8828557; doi:10.1016/j.stemcr.2021.12.016)
Supplement: Document S1. Figures S1–S6, Table S1, and Supplemental experimental procedures [file mmc1.pdf]

Stem Cell Reports, Volume *17*

Supplemental Information

Modeling reduced contractility and impaired desmosome assembly due to plakophilin-2 deficiency using isogenic iPS cell-derived cardiomyocytes

Hiroyuki Inoue, Satoki Nakamura, Shuichiro Higo, Mikio Shiba, Yasuaki Kohama, Takumi Kondo, Satoshi Kameda, Tomoka Tabata, Shota Okuno, Yoshihiko Ikeda, Junjun Li, Li Liu, Satoru Yamazaki, Maki Takeda, Emiko Ito, Seiji Takashima, Shigeru Miyagawa, Yoshiki Sawa, Shungo Hikoso, and Yasushi Sakata

Supplemental Information

Modeling Reduced Contractility and Impaired Desmosome Assembly due to Plakophilin-2 Insufficiency using Isogenic Induced Pluripotent Stem Cell-Derived Cardiomyocytes

Hiroyuki Inoue[1,#], Satoki Nakamura[2,#], Shuichiro Higo[3,*], Mikio Shiba[1], Yasuaki Kohama[4], Takumi Kondo[1], Satoshi Kameda[1], Tomoka Tabata[1], Shota Okuno[1], Yoshihiko Ikeda[5] , Junjun Li[6,7], Li Liu[6,7], Satoru Yamazaki[8], Maki Takeda[6], Emiko Ito[6], Seiji Takashima[9], Shigeru Miyagawa[6], Yoshiki Sawa[6], Shungo Hikoso[1], Yasushi Sakata[1]

[1] Department of Cardiovascular Medicine, Osaka University Graduate School of Medicine, Suita, Osaka, 565-0871, Japan

[2] Osaka Police Hospital, Osaka 543-0035, Japan

[3] Department of Medical Therapeutics for Heart Failure, Osaka University Graduate School of Medicine, Suita, Osaka, 565-0871, Japan

[4] National Hospital Organization, Osaka-Minami Medical Center, Kawachinagano, Osaka 586-8512, Japan

[5] Department of Pathology, National Cerebral and Cardiovascular Center, Suita, Osaka 564-8565, Japan

[6] Department of Cardiovascular Surgery, Osaka University Graduate School of Medicine, Suita, Osaka 565-0871, Japan

[7] Department of Design for Tissue Regeneration, Osaka University Graduate School of Medicine, Suita,

Osaka 565-0871, Japan

[8] Department of Molecular Pharmacology, National Cerebral and Cardiovascular Center, Suita, Osaka 564-8565, Japan.

[9] Department of Medical Biochemistry, Osaka University Graduate School of Medicine, Suita, Osaka 565-0871, Japan

# These authors contributed equally to the work.

***Corresponding Author**

Shuichiro Higo, Associate Professor

Medical Therapeutics for Heart Failure, Osaka University Graduate School of Medicine

Address: 2-2 Yamadaoka, Suita, Osaka 565-0871, Japan Tel: +81-6-6879-3298, Fax: +81-6-6879-3299,

E-mail: higo-s@cardiology.med.osaka-u.ac.jp

Supplementary_Figure_1

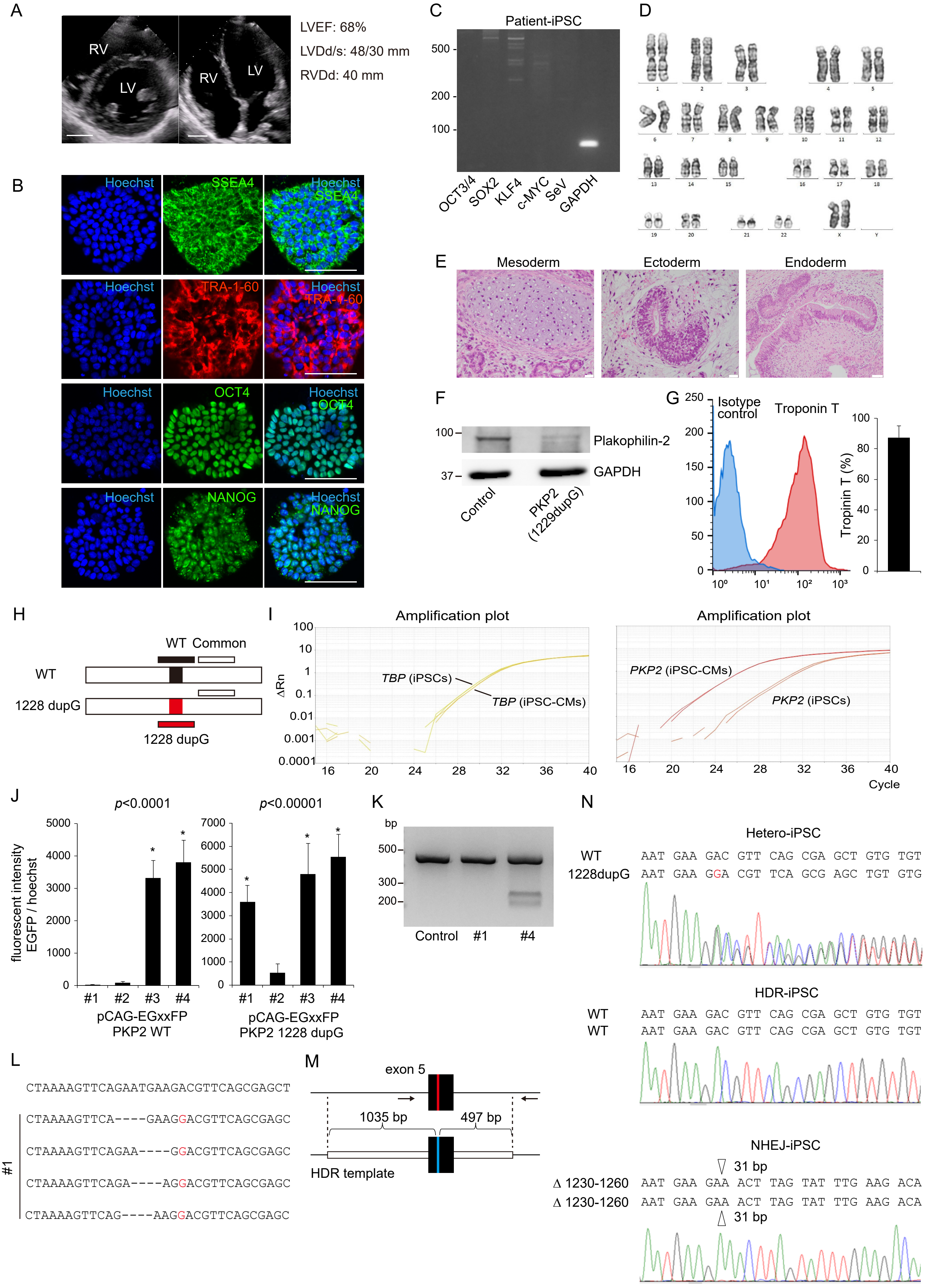

Supplementary_Figure_2

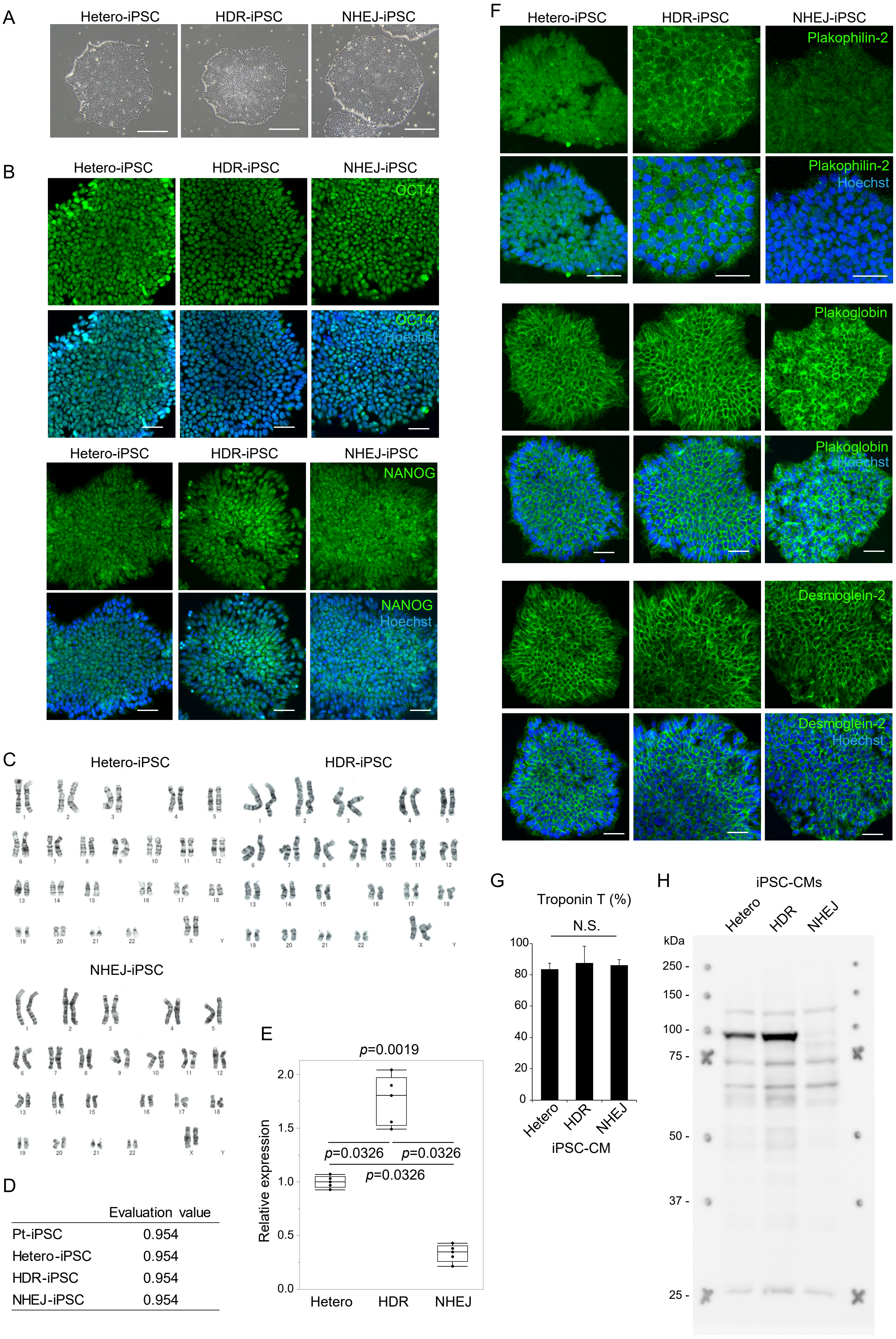

Supplementary_Figure_3

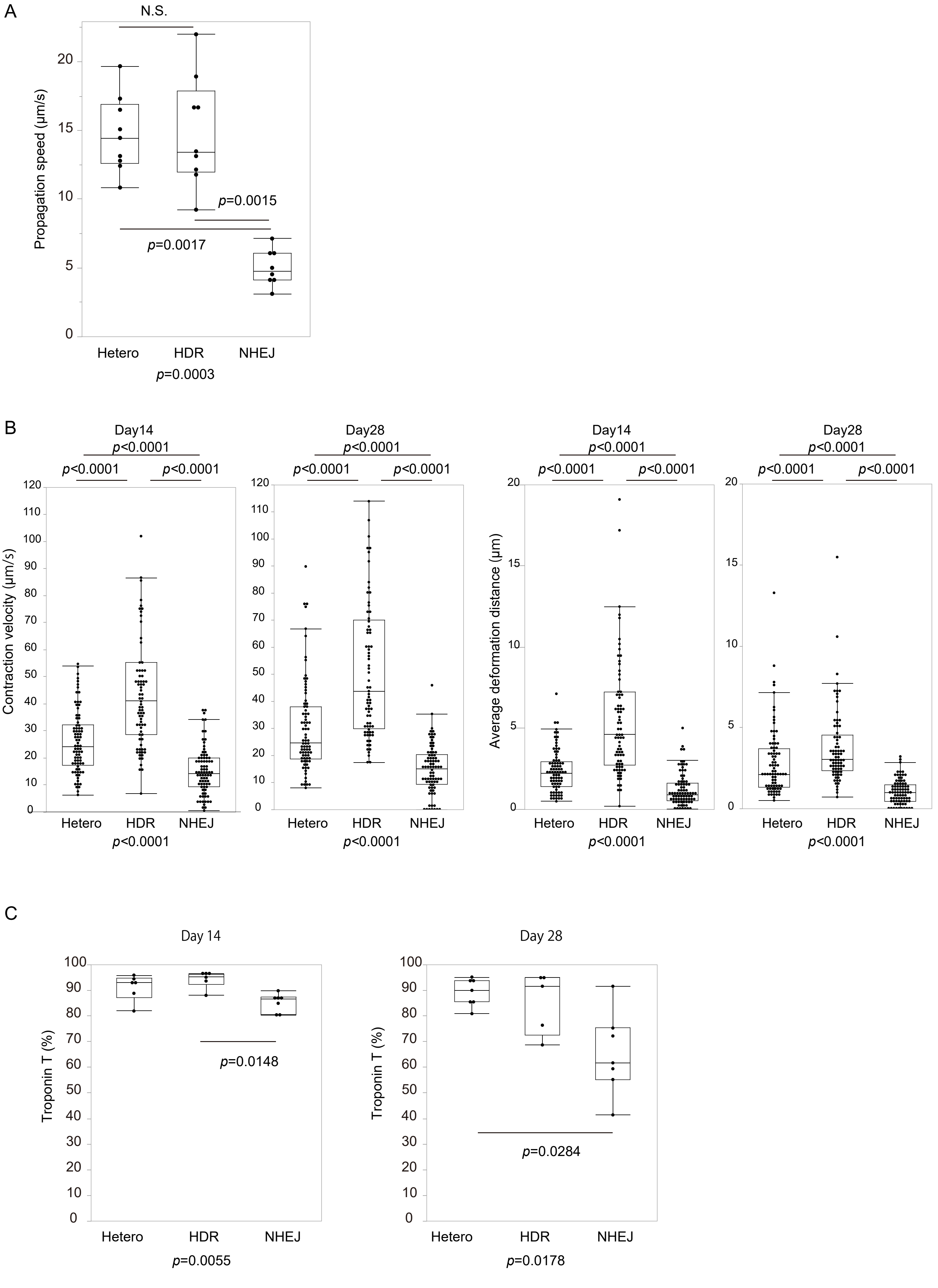

Supplementary_Figure_4

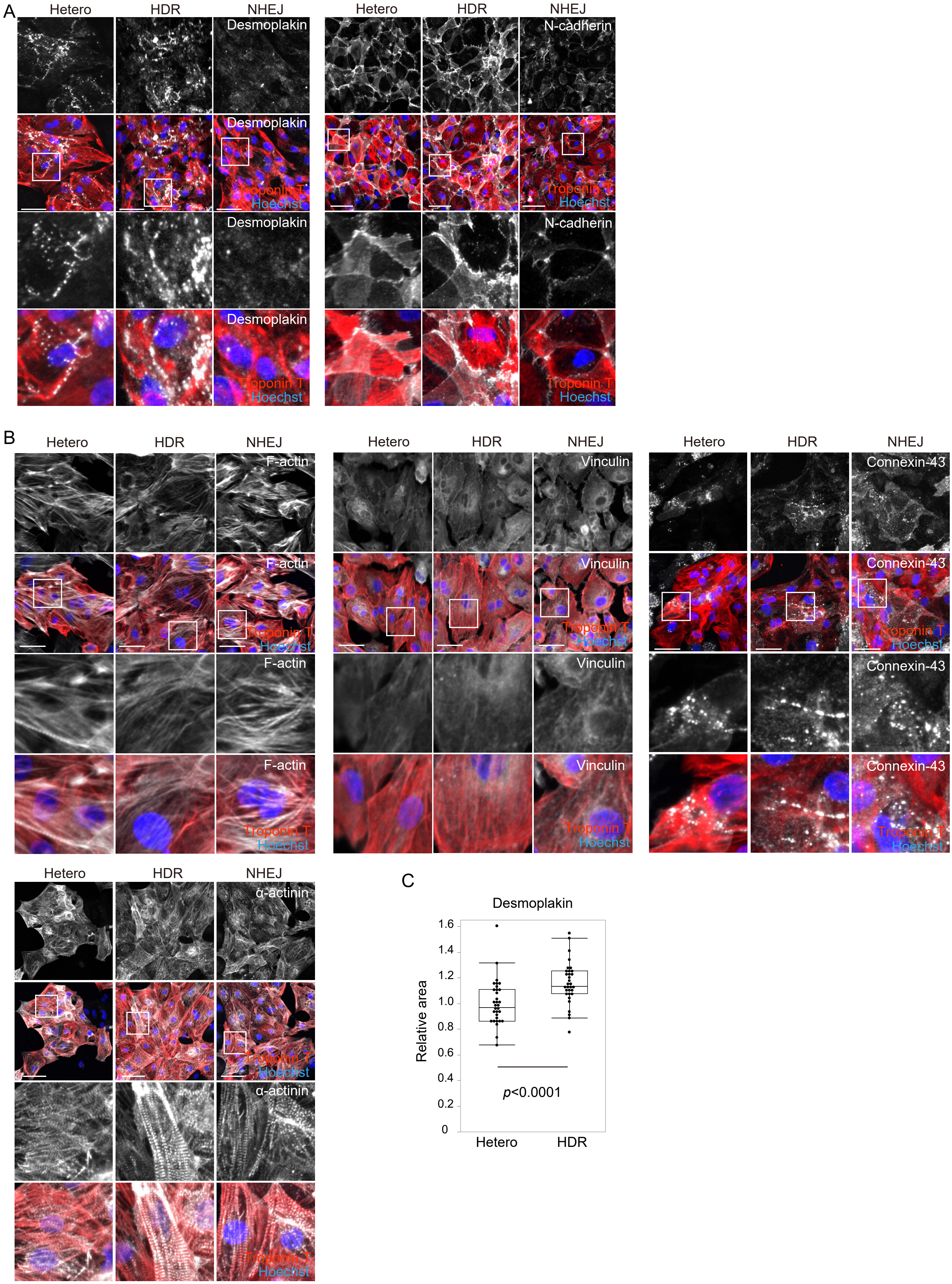

Supplementary_Figure_5

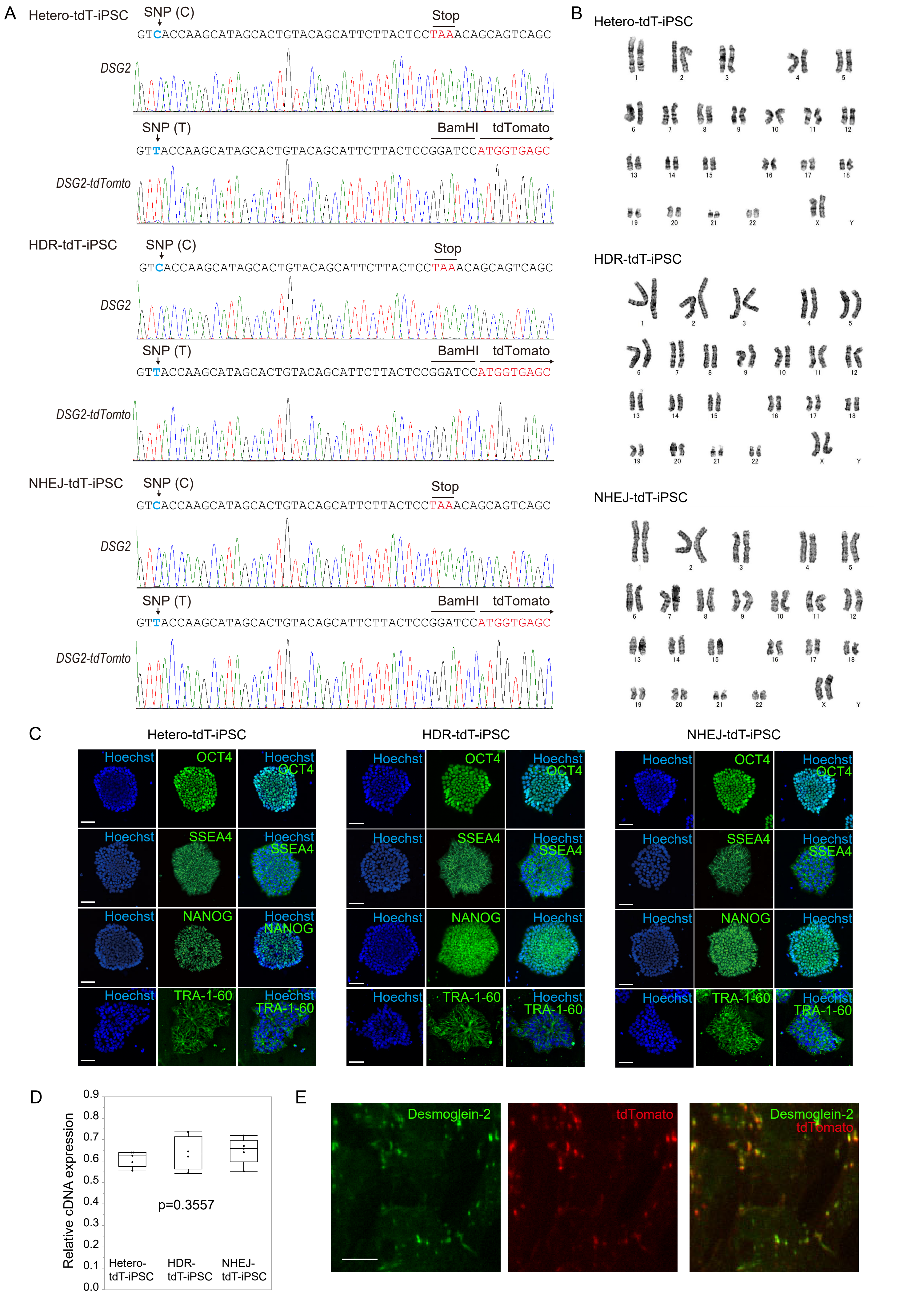

Supplementary_Figure_6

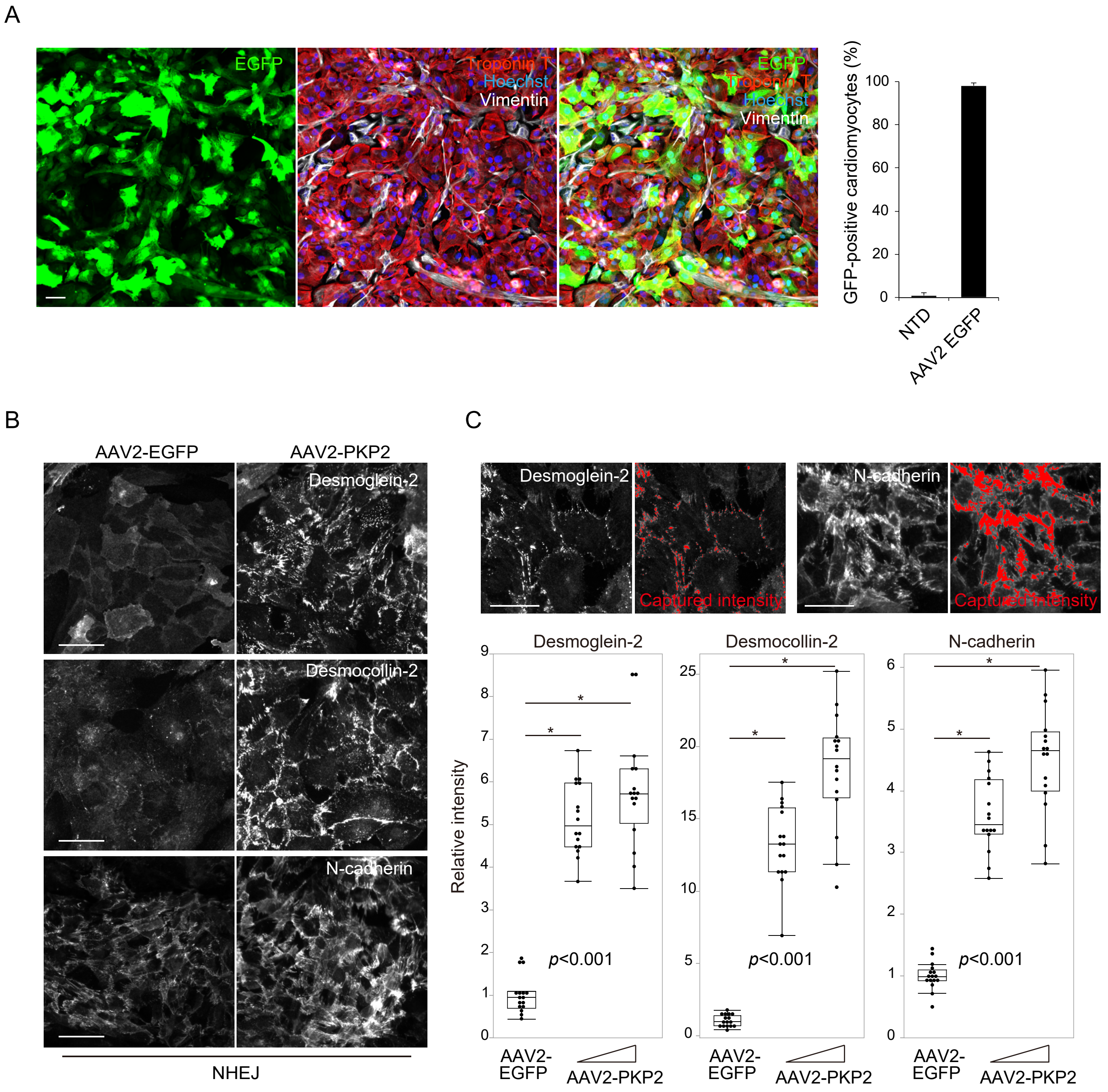

Supplementary Figure Legends

Supplementary Figure 1

A) Parasternal left ventricle short-axis view (left) and four-chamber view (right) of the patient's echocardiogram. LV: left ventricle, RV: right ventricle, EF: ejection fraction, LVDd/s: left ventricular diastolic and systolic diameters, RVDd: right ventricular diastolic diameter. Scale bar: 20 mm.

B) The patient-derived iPSCs were fixed and immunostained with the indicated antibodies against the pluripotent marker proteins. Scale bar: 50 μm.

C) PCR was performed to detect the transgenes generated from residual Sendai viral vectors using the cDNA obtained from patient-derived iPSCs. iPS Transgene/SeV detection primer set (#IDT-DV0301) was used for PCR analysis.

D) Karyotype analysis of the patient-derived iPSCs that were generated.

E) Teratoma formation after subcutaneous injection of iPSCs into NOG mice. Representative H&E staining images of teratoma, including chondrocyte (mesoderm), neuron (ectoderm) and enteron (endoderm) are shown. Scale bars in Mesoderm and Ectoderm: 20 μm. Scale bar in Endoderm: 50 μm.

F) Whole cell lysates were extracted from iPSCs generated from the healthy control or the patient with the *PKP2* mutation and were analyzed by western blotting, using the indicated antibodies.

G) Representative result of FACS analysis in iPSC-CMs after monolayer differentiation. The proportion of troponin T-positive cells were calculated (three independent experiments, mean ± SD).

H) Common qPCR probe that detects both WT and 1228 dupG transcripts and the specific probes that

detect either the WT or 1228 dupG transcript of *PKP2* were designed.

I) Total RNA was extracted from both the patient-derived iPSCs and iPSC-CMs and reverse transcribed to generate cDNA. Quantitative real-time PCR was conducted using the probes targeting *PKP2* (assay ID: qHsaCIP0027871) or *TBP* (assay ID: dHsaCPE5058363). Representative amplification plots are shown. The expression level of *TBP* was used as internal control because the cycle threshold (Ct) value of *TBP* estimated by quantitative real-time PCR was similar to that of *PKP2* in iPSCs, and the Ct value of *TBP* was comparable between iPSCs and iPSC-CMs.

J) The cleaving activities of each gRNA were evaluated using a single strand annealing assay (ANOVA followed by post hoc test (Tukey-Kramer test), *: $p < 0.001$ vs. #2 in WT, *: $p < 0.001$ vs. #2 in 1,228 dupG, five independent experiments, mean ± SD). gRNA #1 specifically cleaved the mutated sequence that contained 1228 dupG but not cleaved WT sequence. gRNA #4 had the highest activity to cleave both WT and 1228 dupG sequence.

K) The cleaving activities of gRNA #1 and #4 targeting the endogenous *PKP2* locus in HEK293T cells were evaluated using the Cel-I assay. gRNA #4 efficiently cleaved the endogenous targeted genomic sequence of *PKP2*.

L) Patient-derived iPSCs were transfected with pX459 encoding gRNA #1. After puromycin selection, genomic DNA was extracted from the iPSC clones. The targeted sequence of *PKP2* was amplified by PCR and cloned into a plasmid vector and sequenced. Representative data from the four individual clones with NHEJ are shown; 1228 dupG is highlighted in red. Genomic cleavages were specifically

introduced into the mutated locus containing the 1228 dupG sequence.

M) Design of HDR repair template, consisting of 1035-bp 5′-terminal and 497-bp 3′-terminal homology arms corresponding to the genomic sequence around exon 5 of *PKP2*. Arrows indicate the positions of PCR primers located inside and outside of the homology arms for sequence analysis.

N) Direct Sanger sequence analysis of the *PKP2* locus using genomic DNA obtained from the isogenic iPS clones that were generated. The duplicated 1228 G sequence is highlighted in red in Hetero sequence. In the NHEJ clone, deletion of the 31 bp sequences was homozygous and occurred in both alleles.

Supplementary Figure 2

A) Bright field images of the isogenic iPSC clones. Scale bar: 250 μm.

B) The isogenic iPS clones cultured in 96-well plates were fixed and immunostained with the indicated antibodies. The nuclei were stained with Hoechst. Bar: 50 μm.

C) Karyotype analysis of the generated isogenic Hetero-, HDR-, and NHEJ-iPSCs.

D) Results of short tandem repeat analysis using genomic DNA obtained from patient-derived iPSCs (Pt-iPSCs), Hetero-, HDR-, and NHEJ-iPSCs. Evaluation value was calculated as the value where (number of coincidental peaks) x 2/total number of peaks in reference sample (genomic DNA obtained from peripheral blood mononuclear cells) + total number of peaks in iPSC sample.

E) Total RNA was extracted both from Hetero-, HDR- and NHEJ-iPSCs. Quantitative real-time PCR was

performed using the common probe that contains the primers amplifying 1517 – 1640 of *PKP2* mRNA (assay ID: qHsaCIP0027871). Obtained data were normalized by the expression levels of *TBP*. Relative expression levels normalized by the value of Hetero-iPSCs are shown (five independent experiments, Kruskal–Wallis test followed by Steel-Dwass test).

F) The isogenic iPS clones cultured in 96-well plates were fixed and immunostained with the indicated antibodies. The nuclei were stained with Hoechst. Bar: 50 μm.

G) Ten days after cardiomyocyte differentiation of the isogenic iPSCs, the proportion of troponin T-positive cells was calculated by FACS analysis (ANOVA, $p = 0.7397$, means ± SD, four independent experiments).

H) Whole cell lysates were extracted from each iPSC-CMs and analyzed by western blot using anti-plakophilin-2 antibody.

Supplementary Figure 3

A) Propagation speed was quantitatively evaluated in Hetero-, HDR- and NHEJ-iPSC-CMs on day 28 using isoclone map data obtained from SI8000 motion analyzer (Kruskal–Wallis test followed by Dunn's test. Number of analyzed images for Hetero-iPSC-CMs: 9, HDR-iPSC-CMs: 9, NHEJ-iPSC-CMs: 8 on day 28, data were collected from three independent experiments).

B) Contraction velocity (CV) and deformation distance (DD) in HDR- and NHEJ-iPSC-CMs on days 14 and 28 under electrical pacing at 1.5 Hz were analyzed using motion vector analysis (Kruskal–Wallis

test followed by Steel-Dwass test). Number of analyzed ROI for all samples were 81. Data were collected from three independent experiments.

C) The proportion of troponin T-positive cells was calculated by FACS analysis. The proportion of troponin T-positive cells was 91.0% vs. 94.0% vs. 85.1% (Hetero- vs. HDR- vs. NHEJ-iPSC-CMs) on day 14, and 88.9% vs. 85.0% vs. 65.0% (Hetero- vs. HDR- vs. NHEJ-iPSC-CMs) on day 28, respectively (Kruskal-Wallis test followed by Steel-Dwass test). Data were collected from six to seven (day 14) and five to seven (day28) independent experiments.

Supplementary Figure 4

A) Hetero-, HDR- and NHEJ-iPSC-CMs were re-plated into 96-well plates at day 10 after differentiation and were subsequently fixed and immunostained at day 14 with the indicated antibodies. Scale bar: 50 μm. High magnification images of the area in the white square are shown in the lower panels.

B) Hetero-, HDR- and NHEJ-iPSC-CMs were re-plated into 96-well plates at day 10 after differentiation and were subsequently fixed and immunostained at day 14 with Alexa Fluor 568 Phalloidin for F-actin staining or the indicated antibodies. Scale bar: 50 μm. High magnification images of the area in the white square are shown in the lower panels.

C) Relative desmosome area of each fluorescent signal in HDR-iPSC-CMs was normalized to that in Hetero-iPSC-CMs (Mann–Whitney test, n = 32 images in each iPSC-CM, data were collected from four independent experiments).

Supplementary Figure 5

A) Sanger sequence analysis of the 3'-terminus of *DSG2* in Hetero-, HDR- and NHEJ-tdT-iPSCs. The upper sequence indicates the allele containing SNP: C and intact 3'-terminus of *DSG2*. The lower sequence indicates the knockin allele containing SNP: T and 3'-terminus of *DSG2* linked to tdToamto sequence.

B) Karyotype analysis of Hetero-, HDR-, NHEJ-tdT-iPSCs.

C) The isogenic iPS clones cultured in 96-well plates were fixed and immunostained with the indicated antibodies. The nuclei were stained with Hoechst. Bar: 50 μm.

D) Relative expression of *DSG2-tdTomato* transcripts normalized by WT *DSG2* transcripts evaluated by ddPCR using cDNA obtained from isogenic tdT-iPSCs (Kruskal-Wallis test, four to five independent experiments).

E) HDR-tdT-iPSC-CMs were fixed and immunostained with anti-desmogelin-2 antibody at day 14 after differentiation. Scale bar: 10 μm.

Supplementary Figure 6

A) NHEJ iPSC-CMs transduced with AAV2-*EGFP* (1.0×10^4 vg/cell) were fixed and immunostained with the indicated antibodies 5 days after transduction. Non-cardiomyocytes were stained with an antibody against vimentin. Scale bar: 50 μm. The proportion of GFP-positive cardiomyocytes were

quantitatively analyzed using high-content imaging (n = 25 images, mean ± SD, data were collected from three independent experiments). NTD indicates the non-transduced control.

B) NHEJ iPSC-CMs transduced with AAV2 encoding *EGFP* or FLAG-tagged *PKP2* were fixed and immunostained with the indicated antibodies 5 days after transduction. Scale bar: 50 μm.

C) The images shown in (B) were quantitatively analyzed by high-content imaging. The upper panels show the raw immunostained images and the captured intensity images detected by high-content imaging. The relative intensities of each fluorescent signal in iPSC-CMs transduced by AAV2-*PKP2* were normalized by those in iPSC-CMs transduced by AAV2-*EGFP* (Kruskal-Wallis test followed by Steel-Dwass test, n = 16 images in each sample, $^{*}p < 0.0001$, data were collected from three independent experiments). 1.0×10^4 viral genomes (vg)/cell of AAV2-*EGFP* and 1.0 or 2.0×10^4 vg/cell of AAV2-*PKP2* were used for transduction.

Supplementary Video 1

Sequential observation of the monolayer iPSC-CMs using motion vector analysis. Bright field images of the fixed positions at specific coordinates of NHEJ-iPSC-CMs at day 14, 18 and 28 are shown.

Supplementary Video 2

Sequential observation of the monolayer of NHEJ-iPSC-CMs using motion vector analysis. Color maps

representing excitation propagation (1/4 speed) of the fixed positions at specific coordinates of NHEJ-iPSC-CMs at days 14 and 19 are shown. At day 14, continuous downward excitation propagation was observed in NHEJ-iPSC-CMs. At day 19, intermittent excitation propagation (2:1 conduction block) in the same fiber structure was observed.

Supplementary Video 3

Supplementary Video 4

Sequential observation of the monolayer iPSC-CMs using motion vector analysis. Bright field images of the fixed positions at specific coordinates of Hetero- (Video 3) and HDR- (Video 4) iPSC-CMs at day 14, 18 and 28 are shown.

Supplementary Video 5

Continuous observation of the restoration process of desmosome assembly in NHEJ-tdT-iPSC-CMs transduced with AAV2-*PKP2*. Images were captured every 30 minutes from 1 to 4 day after the transduction.

Supplementary Table

Oligo DNA	sequence (5'-3')	Application
gRNA against human PKP2 #1	CTCCTAAAAGTTCAGAATGA	sgRNA sequence targeting exon 5 in PKP2
gRNA against human PKP2 #2	GTCCTTCATTCTGAACTTTT	sgRNA sequence targeting exon 5 in PKP2
gRNA against human PKP2 #3	GACGTTCAGCGAGCTGTGTG	sgRNA sequence targeting exon 5 in PKP2
gRNA against human PKP2 #4	ACGTTCAGCGAGCTGTGTGT	sgRNA sequence targeting exon 5 in PKP2
gRNA against human DSG2	TTAAACTCTGGGTCAGTTTG	sgRNA sequence targeting exon 15 in DSG2
Forward primer to amplify HDR template (PKP2)	CCCATTCCTGGTTGTGCCCT	cloning of HDR template (5'- and 3'-terminal homology arms of PKP2)
Reverse primer to amplify HDR template (PKP2)	ATTAGCCAGGTGTGGTAGCA	cloning of HDR template (5'- and 3'-terminal homology arms of PKP2)
Forward primer to amplify genomic sequence around 1228 dupG in PKP2 (BamHI recognition site is added for subcloning)	ggatccACAAGAGCCTCAGTTGTGCT	cloning for pCAG-EGxxFP vector, Cel-I assay to detect genomic cleavage at PKP2 locus<br>PCR for sequence confirmation after sibselection
Reverse primer to amplify genomic sequence around 1228 dupG in PKP2 (EcoRI recognition site is added for subcloning)	gaattcAGGCATCTGGCTGGGGTGCAA	cloning for pCAG-EGxxFP vector, Cel-I assay to detect genomic cleavage at PKP2 locus
Reverse primer to amplify genomic sequence around 1228 dupG in PKP2	CAGTGGCTCATGGCTCATGC	PCR for sequence confirmation after sibselection
Forward primer to amplify genomic sequence around tdTomato knockin site (SphI recognition site is added for subcloning)	gcatgcTCTTTGCAAGAAGCCAATGC	cloning of HDR template (5'- terminal homology arm of DSG2-tdTomato)
Reverse primer to amplify genomic sequence around tdTomato knockin site (BamHI recognition site is added for subcloning)	ggatccGGAGTAAGAATGCTGTACAGT	cloning of HDR template (5'- terminal homology arm of DSG2-tdTomato)
Forward primer to amplify genomic sequence around tdTomato knockin site (HpaI recognition site is added for subcloning)	gttaacTAAACAGCAGTCAGCCACAAACT	cloning of HDR template (3'- terminal homology arm of DSG2-tdTomato)
Reverse primer to amplify genomic sequence around tdTomato knockin site (AflII recognition site is added for subcloning)	cttaagCAAACTCCTGACCACAGTTGA	cloning of HDR template (3'- terminal homology arm of DSG2-tdTomato)
Forward primer to amplify genomic sequence to distinguish the tdTomato knocked-in allele	GCAATCCAGTTACCAGATTCCCAC	PCR for sequence confirmation after sibselection
Reverse primer to amplify genomic sequence to distinguish the tdTomato knocked-in allele	GAAAGTCTGCTGGGTGCAGT	PCR for sequence confirmation after sibselection
OCT3/4_Fw	CCCGAAAGAGAAAGCGAACCAG	detection of Sendai virus-mediated transgenes in iPSCs
OCT3/4_Rv	AATGTATCGAAGGTGCTCAA	detection of Sendai virus-mediated transgenes in iPSCs
SOX2_Fw	ACAAGAGAAAAAACATGTATGG	detection of Sendai virus-mediated transgenes in iPSCs
SOX2_Rv	ATGCGCTGGTTCACGCCCGCGCCCAGG	detection of Sendai virus-mediated transgenes in iPSCs
KLF4_Fw	ACAAGAGAAAAAACATGTATGG	detection of Sendai virus-mediated transgenes in iPSCs
KLF4_Rv	CGCGCTGGCAGGGCCGCTGCTCGAC	detection of Sendai virus-mediated transgenes in iPSCs
c-MYC_Fw	TAACTGACTAGCAGGCTTGTCG	detection of Sendai virus-mediated transgenes in iPSCs
c-MYC_Rv	TCCACATACAGTCCTGGATGATGATG	detection of Sendai virus-mediated transgenes in iPSCs
SeV_Fw	GGATCACTAGGTGATATCGAGC	detection of Sendai virus-mediated transgenes in iPSCs
SeV_Rv	ACCAGACAAGAGTTTAAGAGATATGTATC	detection of Sendai virus-mediated transgenes in iPSCs

Probe for ddPCR or qRT-PCR	Assay ID	
PKP2 WT (HEX), 1228 dupG (FAM)	dMDS329472318	ddPCR probe (annealing temperature: 53°C)
PKP2	qHsaCIP0027871	ddPCR probe (annealing temperature: 54°C)
TBP	dHsaCPE5058363	ddPCR probe (annealing temperature: 53°C)

Custom made primers and probes for ddPCR	Sequence (5'-3')	
Forward primer to amplify genomic sequece around SNP of DSG2	CTGGTCATTCTAATTCTAC	ddPCR probe (annealing temperature: 52°C)
Reverse primer to amplify genomic sequece around SNP of DSG2	GGAGTAAGAATGCTGTA	ddPCR probe (annealing temperature: 52°C)
Probe to detect DSG2 SNP:T (HEX)	ACCAGAGTTACCAAGCA	ddPCR probe (annealing temperature: 52°C)
Probe to detect DSG2 SNP:C (FAM)	ACCAGAGTCACCAAGC	ddPCR probe (annealing temperature: 52°C)

Supplemental Experimental Procedures

Reagents and antibodies

Antibodies used in this study

Oct-3/4 (C-10) (Santa Cruz Biotechnology, Dallas, Texas, USA, Cat# sc-5279, RRID: AB_628051, x500 dilution), TRA-1-60 (Merck Millipore, Burlington, Massachusetts, USA, Cat# MAB4360, RRID: AB_2119183, x400 dilution), SSEA-4 (Merck Millipore, Cat# MAB4304, RRID: AB_177629, x200 dilution), Nanog (Abcam, Cambridge, MA, USA, Cat# ab80892, RRID: AB_2150114, x200 dilution), DSG2 (AH12.2) (Santa Cruz Biotechnology, Cat# sc-80663 RRID: AB_2093438, x1000 dilution (immunostaining), x2000 dilution (western blot)), PKP2 (PROGEN, Germany, Cat#651167, x50 dilution (immunostaining)), PKP2 (Abcam Cat# ab151402, x1000 dilution (western blot)), Plakoglobin (Cell Signaling Technology, Tokyo, Japan, Cat# 2309, RRID: AB_823448, x1000 dilution (immunostaining), x2000 dilution (western blot)), Desmocollin-2/3 (7G6) (Thermo Fisher Scientific, Waltham, Massachusetts, USA, Cat#32-6200, RRID:AB_2533090, x1000 dilution (immunostaining), x2000 dilution (western blot)), Desmoplakin (Abcam, Cambridge, MA, USA, Cat# ab16434, RRID:AB_443375, x200 dilution (immunostaining)), Connexin 43 (Cell Signaling Technology, Cat# 3512, RRID: AB_2294590, x2000 dilution), Troponin T (Abcam Cat# ab64623, RRID:AB_1139590, x1000 dilution (immunostaining), x1000 dilution (western blot)), GAPDH (Santa Cruz Biotechnology Cat# sc-47724, RRID: AB_627678, x2000 dilution (western blot)), Sarcomeric Alpha Actinin (EA-53) (Abcam, Cat# ab9465, RRID: AB_307264, x1000 dilution (immunostaining), x2000 dilution (western blot)), Vinculin (Sigma-Aldrich Cat# V9131, RRID:AB_477629,

x1000 dilution (immunostaining)), Vimentin (Abcam Cat# ab24525 RRID:AB_778824, x1000 dilution (immunostaining)), Alexa Fluor™ 568 Phalloidin (Thermo Fisher Scientific, Waltham, Massachusetts, USA, A12380, x2000 dilution (F-actin staining)), BV421 Mouse IgG1, k Isotype Control (BD Bioscience, Tokyo, Japan, Cat# 562438, RRID: AB_2721018, x300 dilution (FACS)), BV421 Mouse Anti-Cardiac Troponin T (BD Bioscience, Cat# 565618, RRID: AB_2739306, x300 dilution (FACS)). Puromycin dihydrochloride (SIGMA, cat# P9620-10ML).

Amplicon sequence analysis

The genomic DNA was extracted from the peripheral blood of the patient using the QIAAmp DNA mini kit (QIAGEN). We prepared the genomic DNA library using the Ion AmpliSeq Library Kit and Ion Ampliseq Cardiovascular Research Panel, which contains 10,430 PCR amplicons covering 404 genes known to harbor mutations affecting cardiovascular function. The sequencing run was conducted using Ion PGM with 318 Chips. Sequencing data were analyzed using TorrentSuite (version 5.2.2, Life Technologies). Variants with a low-quality score less than 30 or with low read depth less than 30 were excluded. Synonymous mutations without amino acid changes were excluded. Variants were classified as benign when they were present in Human Genetic Variation Database (HGVD)(Higasa et al., 2016; Narahara et al., 2014), ESP 6500 database(Fu et al., 2013; Tennessen et al., 2012), 1000 genomes database(Genomes Project et al., 2015), or in the ExAC database(Lek et al., 2016) with an allele frequency more than 1%. The heterozygous frameshift mutation (c.1228 dupG, p.D410fs) in *PKP2* was not reported in either HGVD, ESP 6500, 1000 genomes,

the ExAC database, or the ARVD/C Genetic Variants Database(van der Zwaag et al., 2009). Pathogenic mutations were not detected in other desmosomal (*DSC2, DSG2, JUP*, and *DSP*) or non-desmosomal (*TMEM43, LMNA, DES, CTNNA3, PLN, TGFB3, TTN, SCN5A, CDH2*) genes.

Cell Culture and cardiomyocyte differentiation

HEK293T cells were maintained in high glucose Dulbecco's Modified Eagle Medium (DMEM, Gibco) supplemented with 10% fetal bovine serum (FBS, Gibco) and penicillin, streptomycin, and glutamine (PSG, Gibco). iPSCs were generated from PBMCs from a patient diagnosed with ARVC harboring the 1228 dupG mutation. PBMCs were separated from the peripheral whole blood using Ficoll–Plaque (GE). Reprogramming was conducted using Sendai virus vectors with OCT3/4, SOX2, KLF4, and c-MYC (CytoTune-iPS 2.0 Sendai Reprogramming Kit, Life Technologies). A total of 12 clones were generated from the patient carrying heterozygous 1228 dupG mutation in *PKP2*. Among the generated clones, the two iPS clones with round shape colony, carrying normal karyotype, with expression of pluripotent markers and normal differentiating ability to cardiomyocytes were selected and cryopreserved. To generate isogenic iPSCs, one iPS clone was transfected with the plasmid vectors for genome editing. At least 24 colonies were selected after electroporation and were screened with Sanger sequencing. We selected HDR-, NHEJ- and Hetero-iPSC (as a control) clones with round shape colony, carrying normal karyotype, with expression of pluripotent markers and normal differentiating ability to cardiomyocytes. These iPSC clones (two original iPSCs, HDR-, NHEJ- and Hetero-iPSCs) were deposited to RIKEN BioResource Research Center. iPSCs

were cultured under feeder-free conditions using StemFit AK02N (AJINOMOTO), as described previously (Nakagawa et al., 2014), on a laminin-coated plate. iPSCs were differentiated into iPSC-CMs using a chemically defined protocol, as previously described(Burridge et al., 2014). The culture medium was exchanged for RPMI 1640 medium (ThermoFisher Scientific, USA) with recombinant human albumin (Sigma-Aldrich) and L-ascorbic acid 2-phosphate (Sigma-Aldrich) for differentiation. iPSCs were treated with CHIR99021 (LC Laboratories, USA) (days 0–2), Wnt-C59 (Selleck Chemicals, USA) and XAV-939 (Cayman) (days 2–4). For sequential observation of HDR-, NHEJ-, and Hetero-iPSC-CMs, differentiated monolayer cardiomyocytes were cultured until day 14 in the RPMI medium. The medium was then exchanged to DMEM supplemented with 10% FBS, 1% penicillin/streptomycin, and 2 mM L-Glutamine (PSG, Gibco, Thermo Fisher Scientific) for further analysis. For continuous observation studies of Hetero- and HDR-iPSC-CMs or for immunostaining experiments followed by high-content imaging, differentiated iPSC-CMs were dissociated with 0.25% Trypsin-EDTA (Gibco, USA) and re-plated into 96-well μClear plates (Greiner) (2×10^4 cells/well) precoated with gelatin (Nitta Gelatin) and incubated with DMEM containing serum.

Transfection of the plasmid components

293T cells were transfected with Lipofectamin 3000 (Invitrogen). iPSCs were transfected by electroporation using NEPA 21 electroporator (Poring pulse: pulse voltage 125 V, pulse width 5 ms, pulse interval 50 ms, pulse number, 2. Transfer pulse: pulse voltage 20 V, pulse width 20 ms, pulse interval 50 ms, pulse number,

5). To introduce a homozygous frameshift mutation via NHEJ that mimics the mutated sequence in *PKP2*, a pX459 vector encoding gRNA #4 was transfected into the patient-derived iPSCs. To replace the mutated sequence via HDR, a pX459 vector encoding gRNA #1 combined with the repair template vector were transfected into patient-derived iPSCs.

Teratoma assay

This animal experiment was conducted properly in compliance with the Guidelines for Animal Experiments of Osaka University. For in vivo teratoma assay, we used immune deficient NOD/Shi-scid, IL-2R γnull mice (NOG mice; female, 7-8weeks) were obtained from In-Vivo Science Inc. (Tokyo, Japan). Fifty microliters (1 × 106) of dissociated hiPSCs were injected into the subcutaneously of the back. After 16 weeks of observation, the subcutaneous tissues were extracted. All surgeries and sacrifices were performed under deep anesthesia enough to minimize the animal suffering. Subcutaneous tissues of NOG mice were fixed with 10% buffered formalin and embedded in paraffin. Serial paraffin-embedded sections cut at a thickness of 0.5 μm were deparaffinized in xylene, dehydrated in a graded series of ethanol, and stained with hematoxylin and eosin.

Droplet digital PCR and quantitative real-time PCR

Droplet digital PCR (ddPCR) was performed as previously described(Gu et al., 2017) using QX200 ddPCR system (BIORAD). To specifically detect the transcripts from WT allele or 1228 dupG allele in *PKP2*, HEX-

or FAM-labeled probe was designed (assay ID: dMDS329472318, BIORAD). To detect both WT and 1228 dupG transcripts, the ready-made FAM-labeled probe for *PKP2* (assay ID: qHsaCIP0027871) was used. After the PCR reaction, the generated droplets were detected and analyzed using QX200 droplet reader (BIORAD). TBP was used for internal control (assay ID: dHsaCPE5058363).

Transfection of plasmids into human iPSCs and selection of targeted clones

Plasmid constructs for genome editing were transfected into iPSCs, as described (Higo et al., 2021; Li et al., 2015), with modifications. Briefly, 5 μg of pX459 plasmid was electroporated into 1×10^5 cells using the NEPA 21 electroporator (poring pulse pulse voltage: 125 V, pulse width: 5 ms, pulse number: 2, NEPA GENE). For HDR-mediated genome editing, 5 μg of repair template DNA plasmid (pCR bluntII-TOPO vector) was additionally transfected. Puromycin (0.3 μg/mL) was added within 48 h after electroporation. Three days after transfection, iPSCs were passaged into 35 mm dishes at a density of 200 cells for clonal colony formation. Simultaneously, genomic DNA was extracted, and genome editing results were evaluated by direct sequencing. After iPSC colonies were formed, at least 24 colonies were picked individually and dissociated into single cells in sterile tubes. The cell suspensions were seeded into two 96-well plates for genotyping and cell expansion. Genomic DNA was extracted; target genomic region was amplified using PCR and evaluated by direct sequencing or sequence analysis after cloning into pCR bluntII-TOPO vectors. To obtain targeted single clone iPSCs, cells were passaged into a new culture dish repeatedly for clonal colony formation.

Motion vector analysis

As described, cell motion profiles of cardiomyocytes differentiated from iPSCs were acquired using the Cell Motion Imaging System (SI8000, SONY) (Hayakawa et al., 2014; Ito et al., 2019). Motion videos were recorded using a 4× objective at a frame rate of 150 fps, with a resolution of 1024 × 1024 pixels. Motion videos were obtained from at least three fields from three wells in each isogenic iPSC-CM cultured in 6- or 12-well plates. In each image, motion parameters were calculated from nine regions of interest (ROIs) with 64 × 64 pixels. Data were acquired from at least three independent experiments. During observation, fixed positions, defined as X- and Y-axes, were sequentially observed. Maximum CV, RV, and average DD during the contraction–relaxation process were calculated as the total area under the CV and RV peaks. CV and DD calculated using the motion vector represent contractile function and contractile force, respectively. Color mapping images allowed visualization of motion propagation of contracting iPSC-CMs. In the measurement under electrical pacing, we stimulated cardiomyocytes by an electrical stimulator (MyoPacer EP, Ion Optix, USA) at 1.5 Hz.

Transmission electron microscopy

iPSC-CMs after monolayer differentiation were fixed with 2.5% glutaraldehyde for 2 h. After fixation with 1% osmium tetraoxide for 90 min, the cells were dehydrated through a graded series of ethanol (50–100%) and propylene oxide and embedded in epoxy resin. Ultrathin sections were cut using an ultramicrotome

(Ultracut E; Reichert-Jung, Vienna, Austria) and stained with uranyl acetate and lead citrate. Microstructures of the differentiated isogenic iPSC-CMs were observed using a TEM (H-7650; Hitachi Co., Tokyo, Japan).

Immunofluorescent staining

iPSCs were seeded into 96-well μClear plates (Greiner) at 1,000 cells/well and incubated at 37ºC for colony formation. Differentiated iPSC-CMs were treated with 0.25% Trypsin-EDTA, suspended with DMEM containing 10% FBS, PSG and 10 μM Y-27632 (Wako) and filtered with 100 μm cell strainer (FALCON). Cardiomyocytes were seeded at 10,000 cells/well into 96-well μClear plates precoated with gelatin (Nitta Gelatin). For immunostaining, cells were fixed with 4% paraformaldehyde for 15 min, permeabilized with 0.5% Triton X-100 for 15 min and blocked with 1% BSA for 30 min at room temperature or overnight at 4°C. Primary antibodies were diluted by 1% bovine serum albumin (BSA), added to each well and incubated for 1 h at room temperature or overnight at 4°C. Secondary antibodies conjugated with Alexa Fluor Dyes (Molecular Probe) including Hoechst 33342 for nuclear staining or Alexa 568-conjugated phalloidin (Thermo) were added and incubated for 30 min at room temperature. All images were acquired using the IN Cell Analyzer 6000 (GE healthcare). For high-content imaging analysis for dot-distributed proteins (desmoglein-2, desmocollin-2 and desmoplakin), 9 – 16 nonoverlap images per well in 96-well plates were obtained using a 20×/0.45NA Nikon lens. The dot-distributed immunofluorescent signals were quantitatively analyzed using IN Cell Developer toolbox (version1.9, GE).

Single strand annealing assays

Targeted genomic sequences were cloned into pCAG-EGxxFP vector(Mashiko et al., 2013) encoding tandem truncated EGFP gene with overlapped sequence separated by the cloning site. pCAG-EGxxFP vector with pX459 vector(Ran et al., 2013) encoding SpCas9 and the indicated sgRNAs were transfected into 293T cells pre-seeded in Greiner CELLSTAR 96-well plate (1×10^4 cells/well). Forty-eight hours after transfection, the fluorescent images of EGFP were obtained by high-content image analysis (IN Cell Analyzer 6000, GE), and quantitatively analyzed using IN Cell Developer Toolbox (GE). A total of 36 nonoverlap images (9 images per well) were obtained from each sample in one experiment using a 10×/0.45NA Nikon lens.

Cel-I assay

HEK293T cells were seeded in a 24 well plate (5×10^4 cells/well) one day before transfection. pX459 vector was transfected into HEK293T cells using Lipofectamine 3000 (Life Technologies). Two days after transfection, the medium was exchanged to the medium containing 1.0 μg/mL puromycin to select the cells expressing Cas9. After puromycin selection, genomic DNA was extracted using QIAamp DNA Mini Kit (QIAGEN). Target regions were amplified by PCR (KOD Fx Neo, TOYOBO) as follows: 94°C for 2 min, followed by 33 cycles of 98°C for 10 s, annealing temperature (depending on primer sequences) for 30 s and 68°C for 30 s. Primer sequences are listed in Supplementary Table. After purification of PCR products using QIAquick PCR purification kit (QIAGEN), PCR fragments both from untreated and treated allele were

hybridized to form hetero DNA duplex. Then, hybridized PCR hetero duplexes were enzymatically digested by mismatch-specific endonuclease, Cel-I at 42°C for 60 min (SURVEYOR Mutation Detection Kit) and electrophoresed.

Plasmid construction

gRNA sequences targeting the genomic region surrounding 1228 dupG mutation in *PKP2* were designed using CRISPR Design Tool(Hsu et al., 2013), and cloned into pX459 vector as previously described(Ran et al., 2013). DNA sequences for 5'-terminal and 3'-terminal homology arms surrounding 1228 dupG mutation in *PKP2* gene were amplified from WT genomic DNA, then cloned into pCR bluntII-TOPO vector (Thermo). Full length human *PKP2* coding sequence was subcloned from ORF clone (Dharmacon) into pENTR/D-TOPO vector (Thermo). For expression in cultured cells, *PKP2* sequence was recombined into pcDNA3.1/nV5-DEST (Thermo) using the Gateway system (Invitrogen). To generate N-terminal FLAG-tagged protein, the FLAG epitope (DYKDDDDK) was inserted before the coding sequence by PCR-based mutagenesis. For AAV generation N-terminally FLAG-tagged full length *PKP2* sequence was subcloned into pAAV vector (TaKaRa).

Generation of AAV and transduction in iPSC-CMs

To generate AAV2, HEK293T cells were transfected with pAAV vector encoding N-terminally FLAG-tagged PKP2 or pAAV vector encoding EGFP as control, pHelper vector and pRC2-mi342 Vector (AAVpro Helper

Free System, TaKaRa) using calcium phosphate transfection (CalPhos Mammalian Transfection Kit,TaKaRa). Seventy-two h after transfection, HEK293T cells were detached by addition of 1/80 volume of 0.5M EDTA (pH 8.0), then pelleted via low-speed centrifugation (2000 x g for 10 min). Cell pellet was lysed with AAV Extraction Solution A and centrifuged (9000 x g for 10 min). AAV Extraction Solution B was added to the collected supernatant and stored at -80 °C. Collected AAV generated from HEK293T cells was purified using AAVpro Purification Kit (TaKaRa), and viral titer was calculated using AAV Titration Kit (TaKaRa). For immunostaining or time-lapse imaging, iPSC-CMs around 10 days after differentiation were replated into 96-well plates (2 x 10^4 cells/well) or glass-based dishes (8 x 10^4 cells/well), then transduced with AAV2 at 1.0 – 2.0 x 10^4 vg/cell. For motion vector analysis or western blotting, iPSC-CMs in 12-well plates (2.0 x 10^6 – 1.0 x 10^7 cells/well) around 10 days after differentiation were transduced with AAV2 at approximately 1.0 x 10^4 vg/cell.

RNA extraction, quantitative real-time PCR

Total RNA was extracted using RNeasy mini kit (QIAGEN) and converted to cDNA using high capacity RNA-to cDNA RT kit (Thermo). Quantitative real-time PCR was performed using SYBR green or probe method (THUNDERBIRD SYBR, probe qPCR mix, TOYOBO). All of the samples were processed in duplicate. The level of each transcript was quantified by the threshold cycle (Ct) method using TBP or GAPDH as internal controls. PCR primers and probes used for quantitative PCR are listed in supplementary table.

Western Blotting

For western blotting, cells were washed with cold PBS and directly lysed with SDS buffer (10% SDS, 50mM Tris-HCl (pH7.4), 5mM EDTA). The protein concentration was determined by BCA Protein Assay Kit (Thermo). Lysate samples were mixed with 4 × Laemmli sample buffer (BIORAD) with mercaptoethanol (2.5%). Proteins were separated by SDS-PAGE and transferred to PVDF membrane. Antibodies were diluted by 3% nonfat milk. After blocking with 3% nonfat milk for 1 h, the transferred membrane was incubated with primary antibody at 4°C overnight and with secondary antibody at room temperature for 30 min. The membrane signals were detected by chemiluminescence using ECL or ECL prime reagent (GE). The protein expression level was quantified using ImageQuant TL (GE). The expression levels of each protein were normalized by those of GAPDH.

Statistical Analysis

Normally distributed data were analyzed by ANOVA followed by post hoc test (Tukey-Kramer test) for comparison in more than three groups and were represented as means ± S.D. Data that were not normally distributed were analyzed by Mann-Whitney test for comparison in two groups, or Kruskall-Wallis test followed by post hoc test (Dunn's test or Steel-Dwass test) for comparison in more than three groups and were represented as box plots. The box plot consists of a box ranging from the 25th quantile to the 75th quantile. The horizontal line in each box indicates the median value. The whiskers indicate 1.5 times the

interquartile range with outliers, or minimum and maximum values otherwise. Statistical analysis was conducted with JMP (SAS, Cary, NC) and we considered a *p*-value of less than 0.05 to be statistically significant.

Reference

Burridge, P.W., Matsa, E., Shukla, P., Lin, Z.C., Churko, J.M., Ebert, A.D., Lan, F., Diecke, S., Huber, B., Mordwinkin, N.M., *et al.* (2014). Chemically defined generation of human cardiomyocytes. Nat Methods *11*, 855-860.

Fu, W., O'Connor, T.D., Jun, G., Kang, H.M., Abecasis, G., Leal, S.M., Gabriel, S., Rieder, M.J., Altshuler, D., Shendure, J., *et al.* (2013). Analysis of 6,515 exomes reveals the recent origin of most human protein-coding variants. Nature *493*, 216-220.

Genomes Project, C., Auton, A., Brooks, L.D., Durbin, R.M., Garrison, E.P., Kang, H.M., Korbel, J.O., Marchini, J.L., McCarthy, S., McVean, G.A., *et al.* (2015). A global reference for human genetic variation. Nature *526*, 68-74.

Gu, M., Shao, N.Y., Sa, S., Li, D., Termglinchan, V., Ameen, M., Karakikes, I., Sosa, G., Grubert, F., Lee, J., *et al.* (2017). Patient-Specific iPSC-Derived Endothelial Cells Uncover Pathways that Protect against Pulmonary Hypertension in BMPR2 Mutation Carriers. Cell Stem Cell *20*, 490-504 e495.

Hayakawa, T., Kunihiro, T., Ando, T., Kobayashi, S., Matsui, E., Yada, H., Kanda, Y., Kurokawa, J., and Furukawa, T. (2014). Image-based evaluation of contraction-relaxation kinetics of human-induced pluripotent stem cell-derived cardiomyocytes: Correlation and complementarity with extracellular electrophysiology. J Mol Cell Cardiol *77*, 178-191.

Higasa, K., Miyake, N., Yoshimura, J., Okamura, K., Niihori, T., Saitsu, H., Doi, K., Shimizu, M., Nakabayashi, K., Aoki, Y., *et al.* (2016). Human genetic variation database, a reference database of genetic variations in the Japanese population. Journal of human genetics *61*, 547-553.

Higo, S., Hikoso, S., Miyagawa, S., and Sakata, Y. (2021). Genome Editing in Human Induced Pluripotent Stem Cells (hiPSCs). Methods Mol Biol *2320*, 235-245.

Hsu, P.D., Scott, D.A., Weinstein, J.A., Ran, F.A., Konermann, S., Agarwala, V., Li, Y., Fine, E.J., Wu, X., Shalem, O., *et al.* (2013). DNA targeting specificity of RNA-guided Cas9 nucleases. Nat Biotechnol *31*, 827-832.

Ito, M., Hara, H., Takeda, N., Naito, A.T., Nomura, S., Kondo, M., Hata, Y., Uchiyama, M., Morita, H., and Komuro, I. (2019). Characterization of a small molecule that promotes cell cycle activation of human induced pluripotent stem cell-derived cardiomyocytes. J Mol Cell Cardiol *128*, 90-95.

Lek, M., Karczewski, K.J., Minikel, E.V., Samocha, K.E., Banks, E., Fennell, T., O'Donnell-Luria, A.H., Ware, J.S., Hill, A.J., Cummings, B.B., *et al.* (2016). Analysis of protein-coding genetic variation in 60,706 humans. Nature *536*, 285-291.

Li, H.L., Fujimoto, N., Sasakawa, N., Shirai, S., Ohkame, T., Sakuma, T., Tanaka, M., Amano, N., Watanabe, A., Sakurai, H., *et al.* (2015). Precise correction of the dystrophin gene in duchenne muscular dystrophy patient induced pluripotent stem cells by TALEN and CRISPR-Cas9. Stem Cell Reports *4*, 143-154.

Mashiko, D., Fujihara, Y., Satouh, Y., Miyata, H., Isotani, A., and Ikawa, M. (2013). Generation of

mutant mice by pronuclear injection of circular plasmid expressing Cas9 and single guided RNA. Sci Rep *3*, 3355.

Nakagawa, M., Taniguchi, Y., Senda, S., Takizawa, N., Ichisaka, T., Asano, K., Morizane, A., Doi, D., Takahashi, J., Nishizawa, M.*, et al.* (2014). A novel efficient feeder-free culture system for the derivation of human induced pluripotent stem cells. Sci Rep *4*, 3594.

Narahara, M., Higasa, K., Nakamura, S., Tabara, Y., Kawaguchi, T., Ishii, M., Matsubara, K., Matsuda, F., and Yamada, R. (2014). Large-scale East-Asian eQTL mapping reveals novel candidate genes for LD mapping and the genomic landscape of transcriptional effects of sequence variants. PLoS One *9*, e100924.

Ran, F.A., Hsu, P.D., Wright, J., Agarwala, V., Scott, D.A., and Zhang, F. (2013). Genome engineering using the CRISPR-Cas9 system. Nat Protoc *8*, 2281-2308.

Tennessen, J.A., Bigham, A.W., O'Connor, T.D., Fu, W., Kenny, E.E., Gravel, S., McGee, S., Do, R., Liu, X., Jun, G.*, et al.* (2012). Evolution and functional impact of rare coding variation from deep sequencing of human exomes. Science *337*, 64-69.

van der Zwaag, P.A., Jongbloed, J.D., van den Berg, M.P., van der Smagt, J.J., Jongbloed, R., Bikker, H., Hofstra, R.M., and van Tintelen, J.P. (2009). A genetic variants database for arrhythmogenic right ventricular dysplasia/cardiomyopathy. Hum Mutat *30*, 1278-1283.
