# Supplementary material for: Modeling reduced contractility and impaired desmosome assembly due to plakophilin-2 deficiency using isogenic iPS cell-derived cardiomyocytes
Source: Stem Cell Reports. 2022 Jan 20;17(2):337–51. doi: 10.1016/j.stemcr.2021.12.016 (PMC8828557; doi:10.1016/j.stemcr.2021.12.016)
Supplement: Document S2. Article plus supplemental information [file mmc7.pdf]

Stem Cell Reports

ISSCR

Article

OPEN ACCESS

Modeling reduced contractility and impaired desmosome assembly due to plakophilin-2 deficiency using isogenic iPS cell-derived cardiomyocytes

Hiroyuki Inoue,[1,10] Satoki Nakamura,[2,10] Shuichiro Higo,[3,*] Mikio Shiba,[1] Yasuaki Kohama,[4] Takumi Kondo,[1] Satoshi Kameda,[1] Tomoka Tabata,[1] Shota Okuno,[1] Yoshihiko Ikeda,[5] Junjun Li,[6,7] Li Liu,[6,7] Satoru Yamazaki,[8] Maki Takeda,[6] Emiko Ito,[6] Seiji Takashima,[9] Shigeru Miyagawa,[6] Yoshiki Sawa,[6] Shungo Hikoso,[1] and Yasushi Sakata[1]

[1]Department of Cardiovascular Medicine, Osaka University Graduate School of Medicine, Suita, Osaka 565-0871, Japan
[2]Osaka Police Hospital, Osaka 543-0035, Japan
[3]Department of Medical Therapeutics for Heart Failure, Osaka University Graduate School of Medicine, Suita, Osaka 565-0871, Japan
[4]Cardiovascular Division, National Hospital Organization, Osaka-Minami Medical Center, Kawachinagano, Osaka 586-8512, Japan
[5]Department of Pathology, National Cerebral and Cardiovascular Center, Suita, Osaka 564-8565, Japan
[6]Department of Cardiovascular Surgery, Osaka University Graduate School of Medicine, Suita, Osaka 565-0871, Japan
[7]Department of Design for Tissue Regeneration, Osaka University Graduate School of Medicine, Suita, Osaka 565-0871, Japan
[8]Department of Molecular Pharmacology, National Cerebral and Cardiovascular Center, Suita, Osaka 564-8565, Japan
[9]Department of Medical Biochemistry, Osaka University Graduate School of Medicine, Suita, Osaka 565-0871, Japan
[10]These authors contributed equally
*Correspondence: higo-s@cardiology.med.osaka-u.ac.jp
https://doi.org/10.1016/j.stemcr.2021.12.016

SUMMARY

Loss-of-function mutations in *PKP2*, which encodes plakophilin-2, cause arrhythmogenic cardiomyopathy (AC). Restoration of deficient molecules can serve as upstream therapy, thereby requiring a human model that recapitulates disease pathology and provides distinct readouts in phenotypic analysis for proof of concept for gene replacement therapy. Here, we generated isogenic induced pluripotent stem cell-derived cardiomyocytes (iPSC-CMs) with precisely adjusted expression of plakophilin-2 from a patient with AC carrying a heterozygous frameshift *PKP2* mutation. After monolayer differentiation, plakophilin-2 deficiency led to reduced contractility, disrupted intercalated disc structures, and impaired desmosome assembly in iPSC-CMs. Allele-specific fluorescent labeling of endogenous *DSG2* encoding desmoglein-2 in the generated isogenic lines enabled real-time desmosome-imaging under an adjusted dose of plakophilin-2. Adeno-associated virus-mediated gene replacement of *PKP2* recovered contractility and restored desmosome assembly, which was sequentially captured by desmosome-imaging in plakophilin-2-deficient iPSC-CMs. Our isogenic set of iPSC-CMs recapitulates AC pathology and provides a rapid and convenient cellular platform for therapeutic development.

INTRODUCTION

Arrhythmogenic cardiomyopathy (AC), defined as an arrhythmogenic heart muscle disorder not explained by ischemic, hypertensive, or valvular disease, is caused by mutations in genes involved in various cellular functions, including desmosomes, ion channels, cytoskeleton, calcium regulation, or sarcomere (Austin et al., 2019; Towbin et al., 2019). AC cases with predominant right ventricular dysfunction have been diagnosed with arrhythmogenic right-ventricular cardiomyopathy (ARVC), which is a rare, life-threatening, intractable disease that leads to adverse ventricular arrhythmia, ventricular dilatation, and reduced cardiac contraction. Focal fatty infiltration, cardiomyocyte loss, and fibrofatty replacement are observed in the heart tissue of patients with AC (Calkins et al., 2017; Haugaa et al., 2016). Analysis of epidemiological data and results from experimental studies using genetically modified mice have revealed that mutations in desmosomal genes (*DSC2*, *DSG2*, *JUP*, *DSP*, and *PKP2*) lead to AC (Awad et al., 2008; Padron-Barthe et al., 2017), with *PKP2* being the most common gene associated with AC. Plakophilin-2 is located in the outer dense plaque of desmosomes, where it interacts with desmosomal cadherins and desmoplakin (Al-Jassar et al., 2013; Padron-Barthe et al., 2017). In mice, the loss of plakophilin-2 during development leads to reduced trabeculation, cytoskeletal disarray, and cardiac wall rupture (Grossmann et al., 2004), suggesting that plakophilin-2 plays a fundamental role in maintaining the structural integrity of cardiomyocytes. In the clinical setting, most *PKP2* mutations identified in patients with AC are heterozygous and lead to late-onset disease (Calkins et al., 2017; Ohno et al., 2013; van Tintelen et al., 2006). This suggests that the haploinsufficiency of *PKP2* gradually affects cardiac function during a substantial latent asymptomatic period. Conversely, patients with compound or digenic heterozygosity of desmosome genes, including *PKP2*, present with a more severe phenotype (Chen et al., 2019; Gandjbakhch et al., 2018; Rigato et al., 2013). In an extremely rare case in humans, homozygous deletion of *PKP2* led to left ventricular non-compaction and patient death at 12 days of

This is an open access article under the CC BY license (http://creativecommons.org/licenses/by/4.0/).

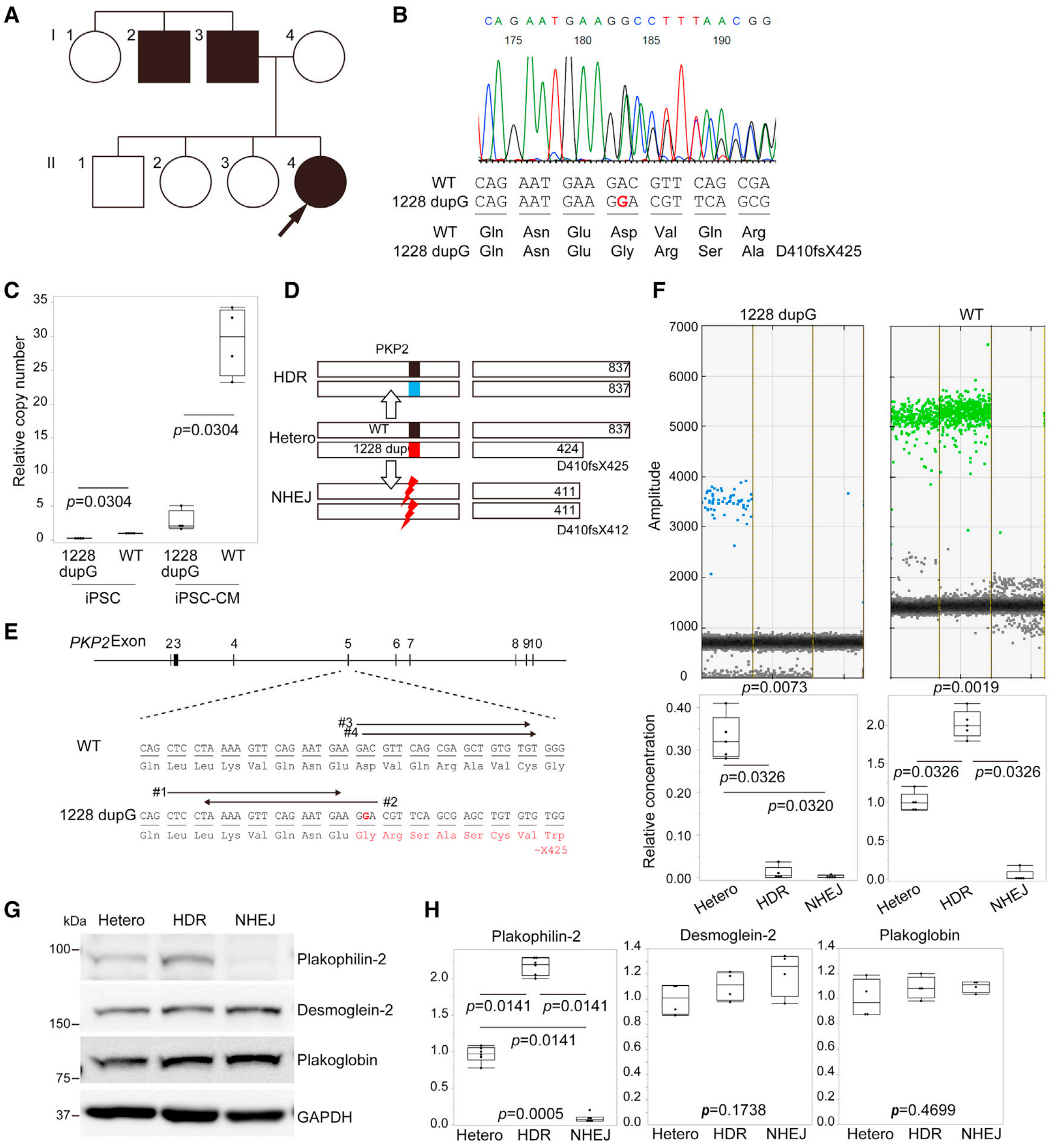

Figure 1. Generation of isogenic iPSCs and differentiation to cardiomyocytes

(A) Family pedigree chart of the proband. Cases who presented with ventricular arrhythmia are shown as black circles (females) or black boxes (males). The proband is indicated by an arrow. The proband's father was diagnosed with AC with a *PKP2* mutation.

(B) Direct Sanger sequence analysis using genomic DNA extracted from the peripheral blood of the patient.

(C) Relative copy number of *PKP2* transcripts in iPSCs and iPSC-CMs was calculated and normalized to that of TATA binding protein (*TBP*) transcripts in each sample. Relative copy number was calculated as the ratio normalized to the levels of WT transcripts in iPSCs (Mann-Whitney test, four independent experiments).

(D) Scheme for generating isogenic iPSCs and the predicted length of plakophilin-2 protein in isogenic iPSC clones.

(E) The targeted site of genome editing around the 1228 dupG mutation in exon 5 of human *PKP2*. gRNA #1 used a mutant AGG sequence as the PAM sequence, and gRNA #2 contained a 20-bp sequence corresponding to the mutant sequence at its 5′ region. gRNA #3 and #4 target the downstream sequence of 1228 dupG.

(legend continued on next page)

age due to severe fetal-onset heart failure (Ramond et al., 2017). For cases with severe clinical phenotypes, restoring gene function may serve as an upstream therapy for the loss of function of *PKP2*. To test this hypothesis, a human cellular model that recapitulates disease pathology and provides distinct readouts for phenotypic analysis is needed. Induced pluripotent stem cell-derived cardiomyocytes (iPSC-CMs) carrying *PKP2* mutations exhibit a significant decrease in the levels of plakophilin-2, plakoglobin, and the gap-junction protein connexin 43 (Caspi et al., 2013; Ma et al., 2013). Distorted desmosomes or clusters of lipid droplets were observed in iPSC-CMs. Findings from a study using iPSC-CMs carrying a homozygous frameshift mutation in *PKP2*, revealed that induction of adult-like metabolism by treatment with adipogenic stimuli exaggerates lipogenesis and apoptosis in iPSC-CMs (Kim et al., 2013). Although these studies demonstrate the pathological role of mutations in *PKP2* in AC iPSC-CMs, the use of control iPSCs derived from healthy individuals cannot completely exclude the influence of different genetic backgrounds. Moreover, how the human *PKP2* mutation affects contractile function in a differentiated monolayer of iPSC-CMs remains unknown.

In this study, we established iPSCs from a patient with AC carrying a heterozygous frameshift *PKP2* mutation and generated an isogenic set of iPSC clones harboring three genotypes (heterozygous mutation, homozygous corrected, and homozygous mutation) using CRISPR/Cas9 genome editing. The arrangement of the haplotype of *PKP2* alleles led to a dose adjustment of protein expression in the isogenic set of iPSC-CMs and demonstrated that plakophilin-2 deficiency led to reduced contractility, disrupted intercalated disc structures, and impaired desmosome assembly in iPSC-CMs. We further generated isogenic lines in which allele-specific fluorescent labeling of endogenous *DSG2* allowed real-time imaging of desmosome assembly under an adjusted dose of plakophilin-2. Adeno-associated virus (AAV)-mediated gene replacement of *PKP2* recovered contractility, and desmosome dynamics during the recovery phase were sequentially captured through desmosome imaging in plakophilin-2-deficient iPSC-CMs. The isogenic set of iPSC-CMs with adjusted levels of *PKP2* expression recapitulates reduced contractility and impaired desmosome assembly and provides a useful cellular model for phenotypic analysis and the development of therapeutics.

RESULTS

Generation of iPSCs from a patient with AC harboring a heterozygous frameshift mutation in *PKP2* and their differentiation to cardiomyocytes

We encountered a 19-year-old female patient diagnosed with AC according to the three major criteria (Marcus et al., 2010), namely repolarization abnormalities in the electrocardiogram, lethal arrhythmias, and family history (Figure 1A), with pathological mutations in *PKP2* (c.1228 dupG, p.D410fsX425,; Figure 1B). Echocardiography revealed that ejection fraction and left-ventricular diameter were within normal limits; however, right-ventricular diameter was slightly dilated (Figure S1A). We screened 404 genes related to inherited cardiovascular disease and confirmed that no deleterious mutations were present in other desmosomal genes, including *DSC2*, *DSG2*, *JUP*, and *DSP*, involved in the etiology of AC. We generated iPSCs from patient-derived peripheral blood mononuclear cells (PBMCs). The generated iPSCs were positive for SSEA4, TRA-1-60, OCT3/4, and NANOG (Figure S1B), negative for Sendai virus-mediated transgenes (Figure S1C), and had a normal karyotype (Figure S1D) and tri-lineage differentiation capacity (Figure S1E). Levels of plakophilin-2 expression were lower in patient-derived iPSCs than in iPSCs generated from a healthy control (Figure S1F). We differentiated the iPSCs into cardiomyocytes according to the chemically defined monolayer protocol, as described (Burridge et al., 2014), which yielded approximately 80%–90% troponin T-positive cardiomyocytes at day 10 after induction of differentiation (Figure S1G). To evaluate the levels of transcript expression from each allele in iPSCs and iPSC-CMs, PCR probes that specifically detect wild-type (WT) and 1228 dupG transcript were used (Figure S1H). For inter-sample comparison of *PKP2* expression between iPSCs and iPSC-CMs, levels of TATA binding protein (*TBP*) were used as internal control (Figure S1I). Results from droplet digital PCR (ddPCR) analysis using cDNA obtained from patient-derived iPSCs and iPSC-CMs revealed that the copy number of the 1228 dupG transcript was 27% lower than that of the WT transcript, and the relative copy number of WT transcripts in iPSC-CMs at day 10 increased approximately 30-fold compared with undifferentiated iPSCs (Figure 1C). The copy number of *PKP2* 1228 dupG transcripts remained low, whereas the copy number of WT *PKP2* transcripts was approximately

(F) Representative positive droplet signals from ddPCR analysis are shown in the top. The concentration (copies/l) of each *PKP2* transcript in the cDNA samples was normalized to that of the *TBP* transcript. Relative copy number was calculated as the ratio normalized to the value of the WT transcript in Hetero-iPSCs (Kruskal-Wallis test followed by Steel-Dwass test, five independent experiments).
(G) Whole-cell lysates were extracted from each iPSC clone and analyzed using western blotting with the indicated antibodies.
(H) Quantification of protein expression normalized to GAPDH expression (Kruskal-Wallis test followed by Steel-Dwass test, four to six independent experiments).

10-fold higher than that of the 1228 dupG transcripts in iPSC-CMs. These data suggest that *PKP2* transcripts containing 1228 dupG were unstable and that the difference in the absolute level of transcripts between WT and 1228 dupG increased in differentiated cardiomyocytes.

Generating a set of isogenic iPSCs using CRISPR/Cas9 genome editing

We generated iPSC clones with the same genetic background and precisely modified genotypes to correct the dosage of *PKP2* transcripts through homology-directed repair (HDR) using CRISPR/Cas9 genome editing. We also aimed to generate the isogenic model expected to exhibit distinct phenotype by introducing a homozygous frameshift mutation through non-homologous end-joining (NHEJ) in *PKP2* of patient-derived iPSCs (Figure 1D). We designed two mutant allele-specific gRNAs (#1 and #2) and common gRNAs (#3 and #4), which targeted both the WT and mutant alleles (Figure 1E). We validated their cleavage ability using the single-strand annealing assay (Mashiko et al., 2013), Cel-I assay, and Sanger sequencing (Figures S1J, S1K, and S1L), and we selected gRNA #1 and #4 for further experiments to induce HDR and NHEJ, respectively. The repair template vector was constructed to replace the mutant sequence with HDR (Figure S1M). After several rounds of sib selection, we obtained an iPSC clone containing homozygous WT alleles (HDR-iPSC) and an iPSC clone containing homozygous frameshift alleles (NHEJ-iPSC). We also obtained a control iPSC clone in which the heterozygous frameshift mutation at *PKP2* remained intact during the same sib selection procedure (Hetero-iPSC) (Figure S1N). The NHEJ clone harbored 31 bp deletions (Δ1230–1260) in both alleles, which were expected to produce truncated plakophilin-2 containing 411 amino acids (Figure 1D). The iPSC clones had uniformly round colonies (Figure S2A), expressed pluripotent markers (Figure S2B), exhibited normal karyotypes (Figure S2C), and had an identical genetic background compared with the patient, as evaluated by short tandem repeat analysis (Figure S2D). Quantitative real-time PCR analysis using a common probe targeting both transcripts (Figure S1H) revealed that relative mRNA expression of *PKP2* was recovered from Hetero- to HDR-iPSCs, and reduced amounts of *PKP2* mRNA, with homozygous frameshift mutations, were transcribed in NHEJ-iPSCs (Figure S2E). ddPCR analysis using a specific probe revealed that mutant transcripts from the 1228 dupG allele were completely abolished in HDR- and NHEJ-iPSCs, and transcripts from the WT allele were recovered in Hetero-to HDR-iPSCs with a 2-fold increase in levels (Figure 1F). Western blot analysis revealed that plakophilin-2 expression was abolished in NHEJ-iPSCs and recovered in HDR-iPSCs (Figures 1G and 1H). Plakoglobin and desmoglein-2, which are encoded by *JUP* and *DSG2*, respectively, are the major components of desmosomes. The expression levels of these proteins are decreased in iPSC-CMs (Caspi et al., 2013; Ma et al., 2013) and myocardium (Rasmussen et al., 2014) in patients with AC with mutations in *PKP2*. In an undifferentiated state, neither protein expression levels nor cellular localization of these junctional proteins was affected in the isogenic iPSCs (Figures 1G, 1H, and S2F). The efficiencies of differentiation, evaluated using fluorescence-activated cell sorting (FACS) using anti-troponin T antibody, were comparable between these cells, and >80% were identified as differentiated without purification (Figure S2G). Truncated plakophilin-2 protein transcribed from the mutant *PKP2* locus containing a frameshift mutation was not detected in either Hetero- or NHEJ-iPSC-CMs (Figure S2H).

Plakophilin-2 insufficiency decreases the contractility of the differentiated monolayer iPSC-CMs

The monolayer differentiation protocol using chemically defined medium produces contractile sheets of cardiomyocytes from iPSCs within 14 days and provides a rapid and convenient platform for functional analysis (Burridge et al., 2014; Sharma et al., 2018). Although reduced contractility and arrhythmogenicity are the major diagnostic criteria of AC (Marcus et al., 2010), how *PKP2* mutation affects the contractility of a differentiated monolayer of iPSC-CMs is unknown. To investigate cell morphology and contractility, differentiated isogenic iPSC-CMs were sequentially evaluated as monolayer cardiomyocytes. Differentiated cardiomyocytes as a monolayer in a chemically defined medium are not suited for long-term adhesion and occasionally detach from the surface (Burridge et al., 2014). We incubated iPSC-CMs without lactate purification and exchanged the medium with serum-containing medium on day 14. This promoted the proliferation of non-cardiomyocytes. However, it allowed us to continuously observe the same culture plate (without the need to replate) and evaluate the kinetic properties of cultured iPSC-CMs in real time using contraction velocity (CV) and deformation distance (DD), representing contractile function and contractile force, respectively, defined using motion vector analysis (Hayakawa et al., 2014; Ito et al., 2019; Wu et al., 2019). From days 8–10, the connected layer structure and coordinated dynamic contraction were similarly observed in both Hetero- and HDR-iPSC-CMs. In NHEJ-iPSC-CMs, hole-like defects, which gradually increased from days 8 to 10, appeared in the connecting cardiomyocytes (Figure 2A), suggesting fragile cell-cell adhesions under increased contractile tension. NHEJ-iPSC-CMs exhibited net-like structures around day 14 and progressive contractile dysfunction over time from days 14 to 28 (Figures 2B, 2C and Video S1). NHEJ-iPSC-CMs exhibited progressive conduction disturbances, as evaluated by color maps

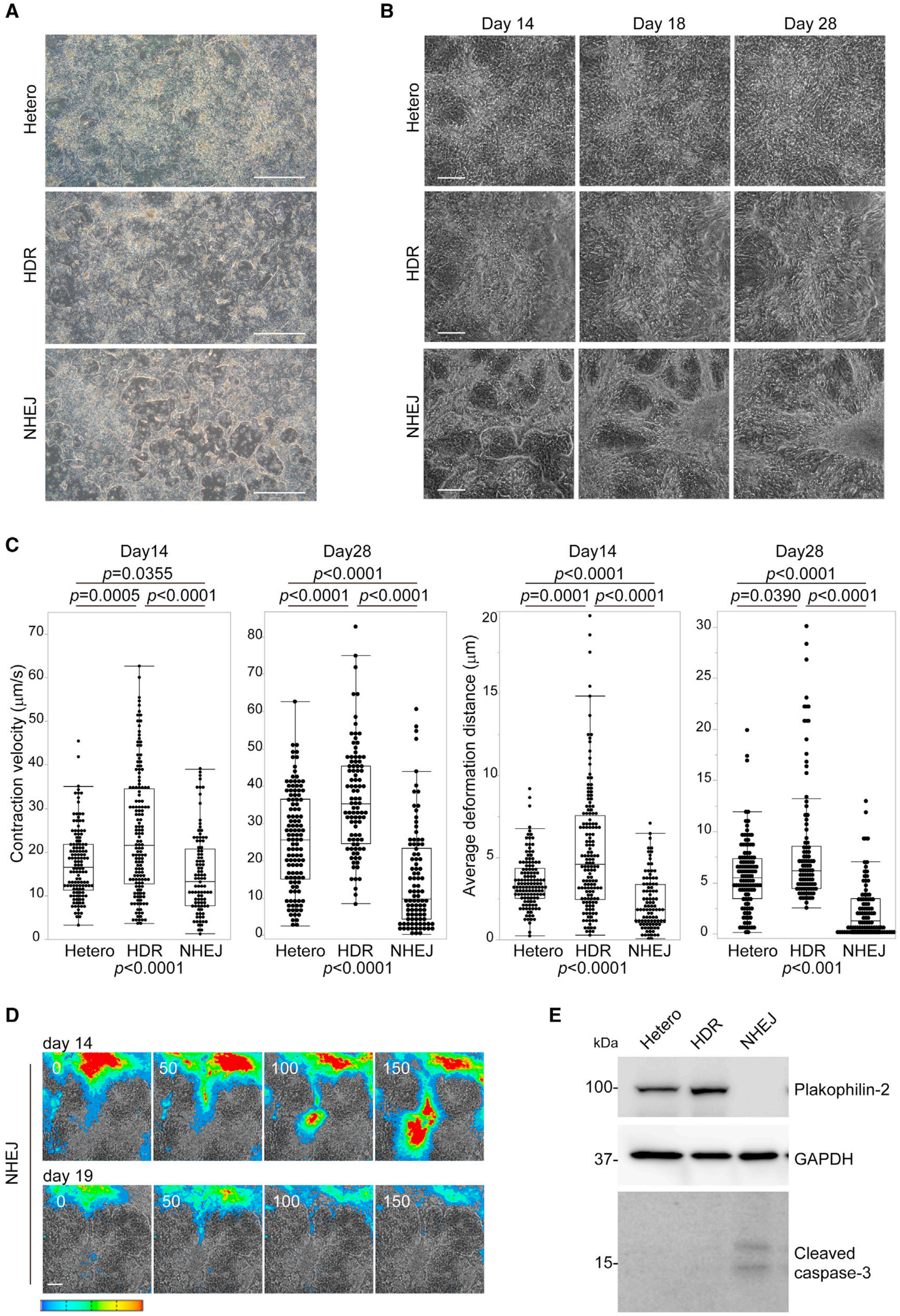

(legend on next page)

converted from motion amplitude (Figure 2D and Video S2), but these parameters were comparable between Hetero- and HDR-iPSC-CMs (Figure S3A). By contrast, Hetero-iPSC-CMs, with a one-half dose of plakophilin-2, did not exhibit apparent morphological differences compared with HDR-iPSC-CMs. However, sequential observation using motion vector analysis clarified that CV and DD were significantly decreased in Hetero-iPSC-CMs compared with the levels in HDR-iPSC-CMs at both day 14 and day 28 after differentiation (Figure 2, Videos S3 and S4). Motion analysis under continuous pacing confirmed the decreased contractility in Hetero- and NHEJ-iPSC-CMs both on day 14 and on day 28 after differentiation (Figure S3B). Increased levels of apoptosis have been observed in iPSC-CMs carrying *PKP2* mutations (Caspi et al., 2013; Kim et al., 2013). Under our experimental conditions, cleaved caspase-3, an apoptosis marker, was detected in NHEJ-iPSC-CMs, and not Hetero- or HDR-iPSC-CMs, 28 days after differentiation (Figure 2). FACS analysis on days 14 and 28 after differentiation demonstrated that the proportion of troponin T-positive cardiomyocytes was gradually decreased in NHEJ-iPSC-CMs compared with Hetero- or HDR-iPSC-CMs (Figure S3C), suggesting the myocyte loss under continuous tension in plakophilin-2-deficient iPSC-CMs. These data suggest that the monolayer differentiation protocol revealed a distinct phenotype in NHEJ-iPSC-CMs and elicited reduced contractility as a pathological phenotype caused by plakophilin-2 haploinsufficiency in Hetero-iPSC-CMs in a short period of time.

Plakophilin-2 insufficiency disrupts intercalated disc structure

Reduced contractility in Hetero-iPSC-CMs within 28 days after monolayer differentiation indicates a damaged microstructure caused by insufficient plakophilin-2. To evaluate differences in subcellular morphology, we fixed isogenic iPSC-CMs 28 days after differentiation and observed them using transmission electron microscopy (TEM). Sarcomere structures with Z-lines were observed in isogenic iPSC-CMs (Figure 3A). NHEJ-iPSC-CMs exhibited significantly increased desmosome gap lengths and severely disrupted intercalated disc structures (Figures 3B and 3C). By contrast, significant morphological abnormalities in desmosomes were not observed in Hetero-iPSC-CMs compared with HDR-iPSC-CMs (Figure 3D). Quantitative analysis targeting desmosomes, represented as electron-dense areas, revealed that, in Hetero-iPSC-CMs, desmosomal gap width was significantly increased compared with HDR-iPSC-CMs (Figure 3C). A TEM study using beating embryoid bodies 40 days after differentiation demonstrated widened and distorted desmosomes in iPSC-CMs with heterozygous *PKP2* frameshift mutations compared with iPSC-CMs generated from healthy controls (Caspi et al., 2013). Our data suggest that microstructural abnormalities were produced in iPSC-CMs at a relatively early phase after differentiation, probably because of the stronger contractile tension promoted by the monolayer differentiation protocol. Studies have reported abnormal lipid accumulation in iPSC-CMs with *PKP2* mutations after differentiation or after additional adipogenic stimulation (Caspi et al., 2013; Kim et al., 2013; Ma et al., 2013). Under our experimental conditions, significant cytosolic lipid droplets were observed in NHEJ-iPSC-CMs 28 days after differentiation (Figure 3E), whereas lipid accumulation was not remarkable in either Hetero- or HDR-iPSC-CMs.

Plakophilin-2 haploinsufficiency impairs desmosome assembly in iPSC-CMs

The monolayer differentiation protocol elicited decreased contraction and disrupted intercalated disc structures caused by plakophilin-2 haploinsufficiency. To identify the initial molecular processes involved, we evaluated the localization and expression levels of desmosomal proteins in isogenic iPSC-CMs using immunostaining and western blotting. At the cell-cell junction, plakophilin-2 expression was recovered in Hetero-to HDR-iPSC-CMs and abolished in NHEJ-iPSC-CMs (Figures 4A, 4B, and 4C). Plakoglobin, an anchoring protein that connects desmosomal cadherins

Figure 2. Plakophilin-2 insufficiency decreases the contractility of the differentiated monolayer iPSC-CMs

(A) Bright-field image of the monolayer Hetero-, HDR-, or NHEJ-iPSC-CMs at day 10 after induction of differentiation. Scale bar: 1 mm.

(B) Sequential observation of the monolayer of iPSC-CMs using motion vector analysis. Bright-field images of fixed positions at specific coordinates on days 14, 18, and 28 are shown. Scale bar: 200 μm.

(C) Contraction velocity (CV) and deformation distance (DD) in HDR- and NHEJ-iPSC-CMs on days 14 and 28 were analyzed using motion vector analysis (Kruskal-Wallis test followed by Steel-Dwass test). Number of analyzed regions of interest (ROI) for Hetero: 129, HDR: 148, and NHEJ: 96 on day 14. Number of analyzed ROI for Hetero: 117, HDR: 94, NHEJ: 94 on day 28. Data were collected from three independent experiments.

(D) Label-free detection of excitation propagation using motion vector analysis excitation. Excitation propagation through the oriented fiber structure was sequentially observed in NHEJ-iPSC-CMs on days 14 and 19. Serial consecutive fluorescence images obtained every 50 ms are shown. Scale bar: 200 μm. Color range from blue to red represents motion velocity from 0 to 30 μm/s, respectively.

(E) Whole-cell lysates were extracted from Hetero-, HDR-, and NHEJ-iPSC-CMs at 28 days after differentiation and analyzed using western blotting with the indicated antibodies.

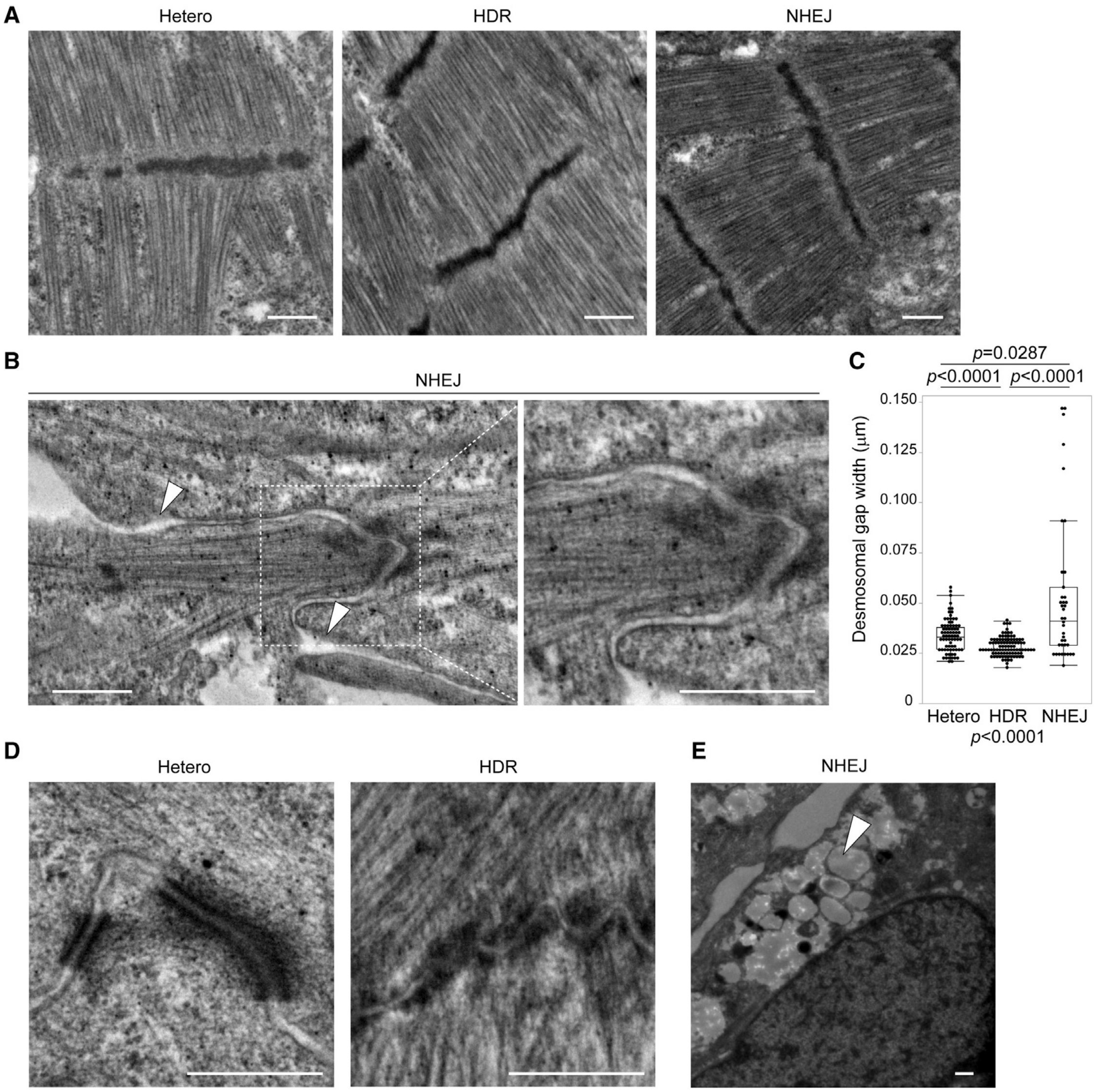

Figure 3. Plakophilin-2 insufficiency disrupts intercalated disc structures

(A) Sarcomere structure in isogenic iPSC-CMs 28 days after differentiation was observed using transmission electron microscopy (TEM). Scale bar: 500 nm.

(B) Representative desmosome structures and dissociated intercalated discs (arrowheads) in NHEJ-iPSC CMs are shown. The area enclosed within the white dotted square is enlarged on the right. Scale bar: 500 nm.

(C) Desmosomal gap width was calculated using TEM images by a blinded operator (Kruskal-Wallis test followed by Steel-Dwass test). Number of analyzed regions in Hetero-iPSC-CMs: 76, HDR-iPSC-CMs: 95, NHEJ-iPSC-CMs: 39. Data were collected from three independent experiments.

(D) Representative desmosome structures in Hetero- and HDR-iPSC-CMs. Scale bar: 500 nm.

(E) Cytosolic accumulation of lipid droplets in NHEJ-iPSC-CMs (arrowhead). Scale bar: 500 nm.

to desmoplakin, is found in both desmosomes and *fascia adherens* (Sheikh et al., 2009). Desmoglein-2 and desmocollin-2 are desmosomal cadherins that form homo- and heteropolymers in intercellular spaces; their C-terminal tails are located in the cytoplasm and are connected to plakophilin-2 (Al-Jassar et al., 2013). Levels of plakoglobin, desmoglein-2, and desmocollin-2 expression were significantly decreased in NHEJ-iPSC-CMs. Plakoglobin remained localized at the cell-cell junctions (Figure 4C), whereas desmoglein-2 and desmocollin-2 were completely dislodged from the cellular periphery and were diffusely distributed in the cytosol in NHEJ-iPSC-CMs (Figure 4D).

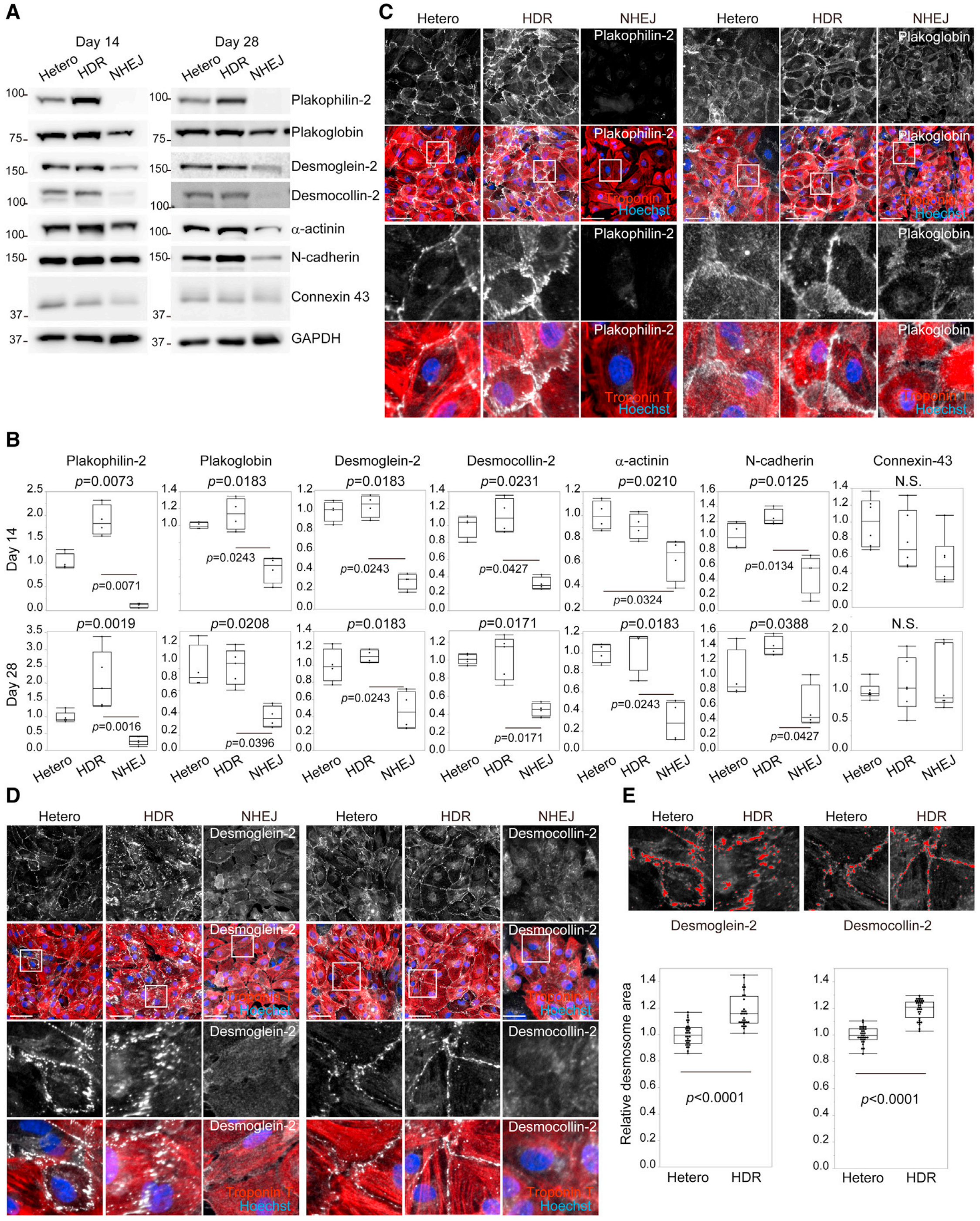

Figure 4. Plakophilin-2 haploinsufficiency leads to impaired desmosome assembly in iPSC-CMs

(A) Whole-cell lysates were extracted from Hetero-, HDR-, and NHEJ-iPSC-CMs on days 14 and 28 after differentiation and analyzed by western blotting using the indicated antibodies.

(B) Quantified protein expression levels normalized by GAPDH expression are shown (Kruskal-Wallis test followed by Dunn's test, four to six independent experiments).

(C and D)Hetero-, HDR-, and NHEJ-iPSC-CMs were replated on 96-well plates at day 10 after differentiation and subsequently fixed and immunostained at day 14 with the indicated antibodies. Scale bar: 50 μm. Areas enclosed within white squares are enlarged at the bottom.

(legend continued on next page)

By contrast, expression levels or cellular localization of desmosomal proteins was not significantly affected in Hetero-iPSC-CMs compared with HDR-iPSC-CMs (Figures 4A, 4B, and 4D), which is consistent with findings that both abundance and localization of intercalated disc proteins are unaffected by plakophilin-2 haploinsufficiency in murine hearts (Cerrone et al., 2012). Peripheral localization of desmoplakin was decreased in NHEJ-iPSC-CMs but was not significantly affected in Hetero-iPSC-CMs (Figure S4A). *Fascia adherens* junctions, which span the extracellular space and link cytoskeletal actin filaments with junction complexes, include transmembrane proteins that are mainly composed of N-cadherin (Lyon et al., 2015). Immunostaining and western blot analysis revealed that N-cadherin expression was significantly decreased in NHEJ-iPSC-CMs but was not significantly different between Hetero- and HDR-iPSC-CMs (Figures 4A, 4B, and S4A). Localization of α-actinin, cytoskeletal actin, or vinculin, which make up the *fascia adherens* network, or expression levels of connexin 43 were not significantly affected in both Hetero- and NHEJ-iPSC-CMs (Figures 4A, 4B, and S4B). Desmoglein-2, desmocollin-2, and desmoplakin were expressed at the cell-cell junctions with punctate distribution in both Hetero- and HDR-iPSC-CMs. To evaluate the effect of plakophilin-2 haploinsufficiency on desmosomal cadherins and desmoplakin, we performed high-content imaging (Ishizu et al., 2017) for quantifying the area of desmosome distribution and found that it was significantly decreased in Hetero-iPSC-CMs compared with HDR-iPSC-CMs (Figures 4E and S4C).These data suggest that the loss of plakophilin-2 affected the stability of intercalated disc proteins, and plakophilin-2 haploinsufficiency did not affect the expression or localization of desmosomal proteins but decreased the area of desmosomes, with punctate distribution after monolayer differentiation.

Allele-specific fluorescent labeling of *DSG2* captures desmosome dynamics in isogenic iPSC-CMs

We recently reported that the loss of desmoglein-2 in human iPSCs does not affect the differentiation process or cell morphology in iPSC-CMs (Shiba et al., 2021). On the basis of these findings, we speculated that fluorescent labeling of endogenous desmoglein-2 could be a useful marker for assessing the degree of deterioration or restoration of desmosomes affected by the insufficient expression of plakophilin-2 in cardiomyocytes. To establish a model for desmosome-imaging, the tdTomato fluorescent reporter was knocked-in at the 3′ terminus of *DSG2* in the three established isogenic iPSCs using genome editing. Because a synonymous single-nucleotide mutation (SNP: T > C) was identified just upstream of the stop codon of *DSG2*, we designed the repair template DNA containing T at the SNP site in the 5′-homology arm to distinguish the knocked-in allele after genome editing (Figure 5A). After repeated rounds of sib selection, followed by PCR and Sanger sequencing (Figures 5B and S5A), we established an isogenic set of iPSCs containing an identical set of *DSG2* alleles in which tdTomato was introduced specifically into SNP: T allele, whereas SNP: C allele remained intact (named Hetero-tdT-, HDR-tdT-, and NHEJ-tdT-iPSC; Figure 5C). All the isogenic tdT-iPSCs generated showed a normal karyotype and were positive for pluripotent markers (Figures S5B and S5C). ddPCR analysis using a specific probe targeting SNP: T or C revealed that *DSG2-tdTomato* transcripts were similarly expressed in isogenic iPSCs at a median rate of 62.5%–65.8% compared with WT *DSG2* transcripts (Figure S5D). Western blotting and immunofluorescence staining demonstrated that the desmoglein-2-tdTomato fusion protein was similarly expressed in isogenic iPSCs (Figures 5D and 5E). Desmoglein-2-tdTomato initially localized at the cell periphery, gradually assembled after differentiation, and then exhibited punctate distribution (Figure 5F). These tdTomato signals were merged with the immunofluorescence signals detected by the anti-desmoglein-2 antibody (Figure S5E). Cellular localization of desmoglein-2-tdTomato fusion protein was similar between Hetero-tdT- and HDR-tdT-iPSC-CMs and not detected in NHEJ-tdT-iPSCMs (Figure 5G). Quantitative analysis using high-content imaging revealed that the desmosome area represented by tdTomato fluorescence in live cells was significantly smaller in Hetero-tdT-iPSC-CMs than in HDR-tdT-iPSC-CMs (Figures 5H and 5I), suggesting that our established isogenic knockin model captured impaired desmosome assembly caused by plakophilin-2 haploinsufficiency in live iPSC-CMs.

AAV-mediated gene delivery of *PKP2* recovered contractility and desmosome assembly in plakophilin-2-deficient iPSC-CMs

To test proof of concept for gene replacement therapy in human cells, we generated an AAV, containing the N-terminal, FLAG-tagged, full-length human *PKP2* sequence driven by the CMV promoter (AAV2-*PKP2*; Figure 6A). We selected the AAV2 serotype because AAV2 has been shown to efficiently transduce iPSC-CMs (Guan et al., 2015), and the use of AAV2 led to high transduction efficiency in iPSC-CMs under our experimental conditions (Kohama et al.,

(E) The images shown in (D) were quantitatively analyzed using high-content imaging. Top: raw immunostained images and captured intensity images detected using high-content imaging. Relative desmosome area of each fluorescent signal in HDR-iPSC-CMs was normalized to that in Hetero-iPSC-CMs (Mann-Whitney test, n = 32 images in each iPSC-CM from four independent experiments).

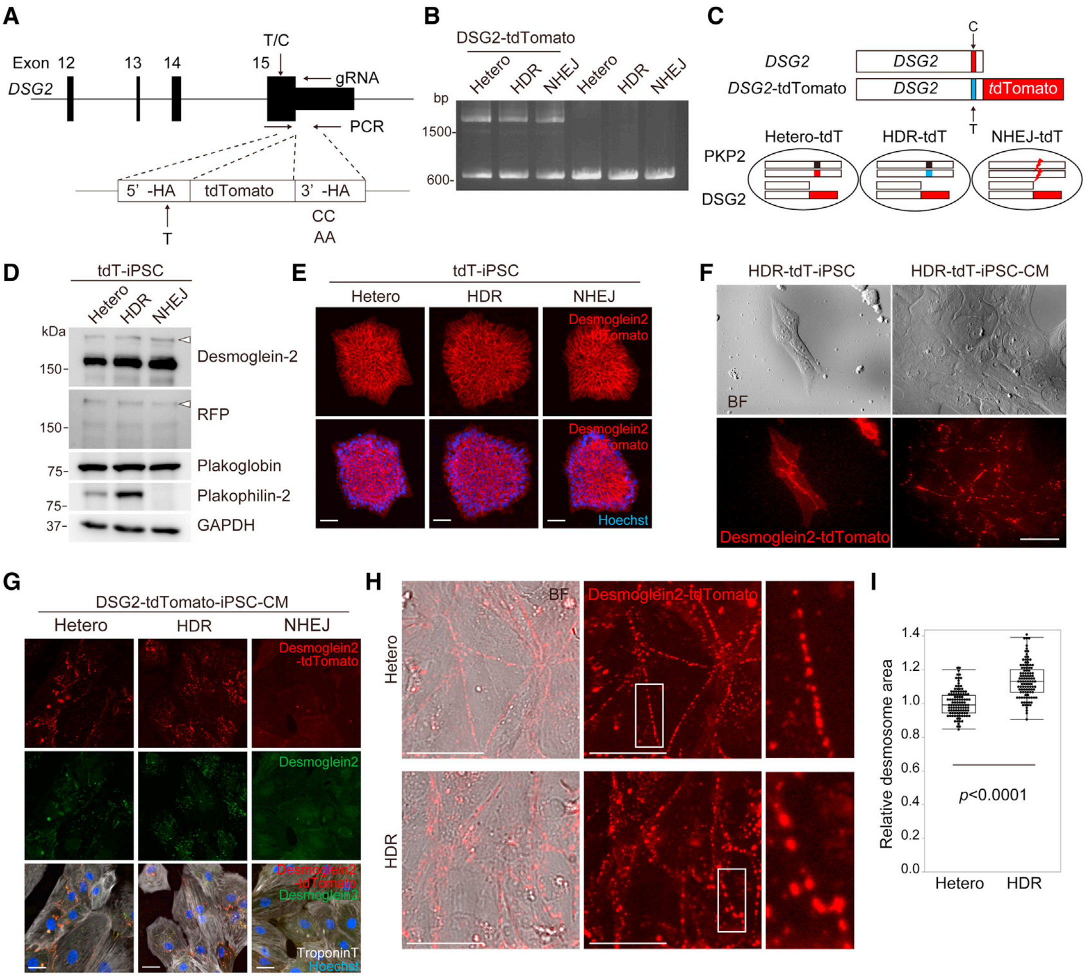

Figure 5. Allele-specific fluorescent labeling of *DSG2* captures desmosome dynamics in isogenic iPSC-CMs
(A) The targeted site of genome editing around the 3′ terminus of exon 15 of human *DSG2*. The repair template DNA contained T at the SNP site in the 5′-homology arm to distinguish the knocked-in allele after genome editing. The 3′-homology arm contained PAM sequence modification from CC to AA to avoid recleavage by Cas9. Arrows indicate the positions of the PCR primers used to distinguish the knocked-in allele.
(B) Electrophoresis of PCR products using genomic DNA extracted from Hetero-, HDR-, NHEJ-tdT-iPSCs, or original isogenic iPSCs. PCR products at 2,277 bp were derived from the knocked-in allele, and those at 632 bp were derived from the non-edited allele.
(C) Scheme showing isogenic iPSCs containing the identical set of *DSG2* alleles in which tdTomato was introduced specifically into the SNP: T allele; the other SNP: C allele remained intact.
(D) Whole-cell lysates were extracted from each iPSC line and analyzed using western blotting with the indicated antibodies. Arrowheads indicate the desmoglein-2-tdTomato fusion protein.
(E) Isogenic tdT-iPSCs were fixed, and nuclei were stained with Hoechst stain. Scale bar: 50 μm.
(F) Live-cell images (bright-field and fluorescence images) of HDR-tdT-iPSCs and HDR-tdT-iPSC-CMs on day 14 after differentiation. Scale bar: 50 μm.
(G) Isogenic tdT-iPSCs were fixed and immunostained with anti-troponin T and desmoglein-2 antibodies. Nuclei were stained with Hoechst stain. Scale bar: 50 μm.
(H) Live-cell images of Hetero- and HDR-tdT-iPSC-CMs 14 days after differentiation were obtained using high-content imaging. Areas enclosed within white squares in the middle are enlarged in at the right. Scale bars: 50 μm.
(I) The images shown in (H) were quantitatively analyzed using high-content imaging. The relative desmosome area of each fluorescent signal in HDR-tdT-iPSC-CMs was normalized to that in Hetero-tdT-iPSC-CMs (Mann-Whitney test, n = 108 images in each iPSC-CM from four independent experiments).

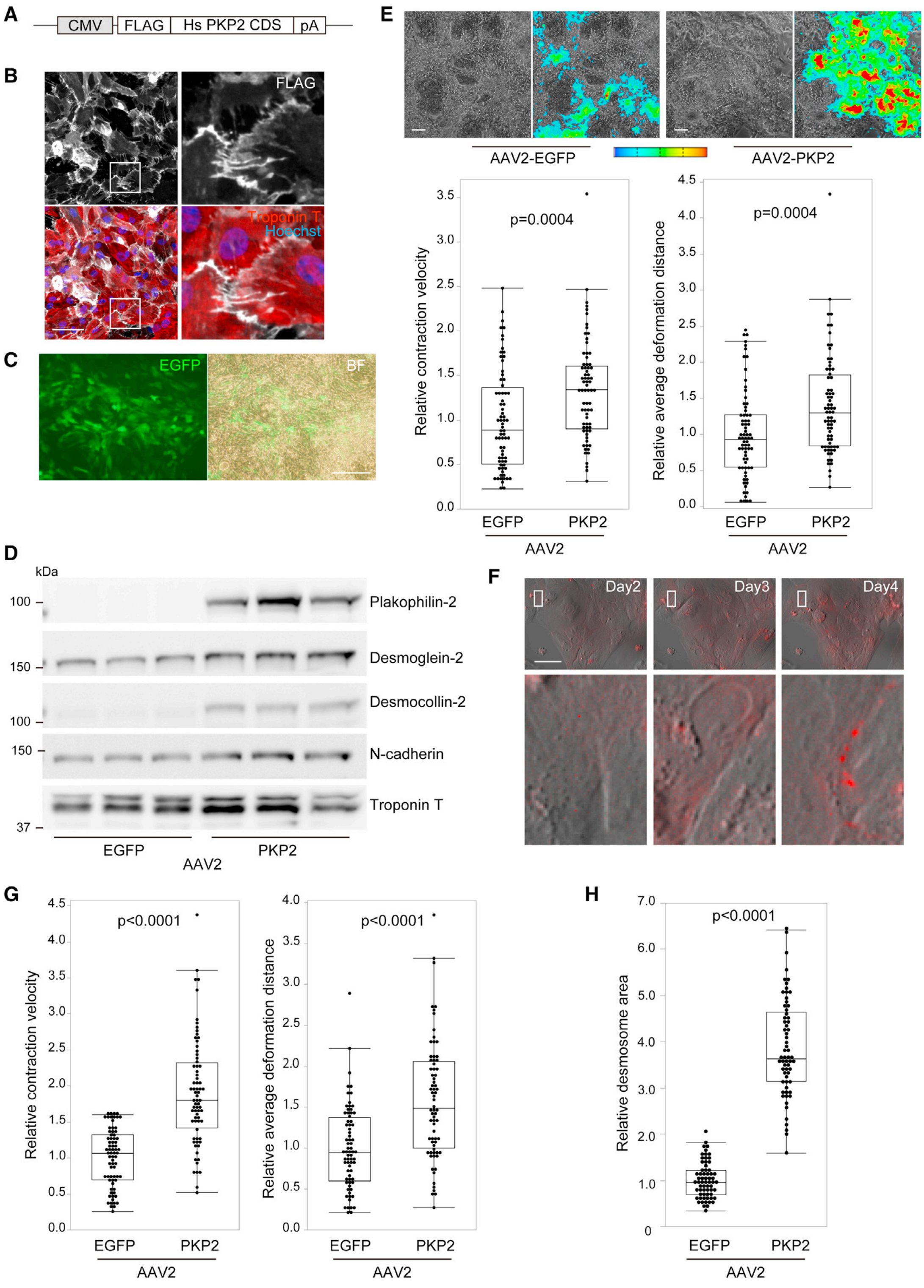

Figure 6. AAV-mediated gene delivery of *PKP2* recovered contractility and desmosome assembly in plakophilin-2-deficient iPSC-CMs

(A) N-terminal FLAG-tagged full-length human *PKP2* coding sequence (Hs *PKP2* CDS) was subcloned into the expression vector with a CMV promoter and poly(A) sequence to generate the AAV2 vector.

(legend continued on next page)

2020; Shiba et al., 2021) (Figure S6A). FLAG-tagged plakophilin-2 protein localized properly at the cellular periphery in iPSC-CMs (Figure 6B), and AAV2-mediated delivery of plakophilin-2 restored the localization of desmoglein-2, desmocollin-2, and N-cadherin in the cell-cell junctions of NHEJ-iPSC-CMs (Figures S6B and S6C). To evaluate the effect of gene replacement in contracting monolayer cardiomyocytes, AAV2-*PKP2* was transduced into NHEJ-iPSC-CMs at day 10, a time when these cells initially exhibited decreased contractility. Fourteen days after transduction, the transgenes were efficiently introduced into the contracting monolayer of iPSC-CMs (Figure 6C). AAV2-mediated replacement of *PKP2* increased the expression of desmoglein-2, desmocollin-2, and N-cadherin (Figure 6D), prevented the formation of hole-like structures in contracting cardiomyocytes, and restored CV and DD, as evaluated by motion analysis (Figure 6E). Notably, time-lapse imaging using NHEJ-tdT-iPSC-CMs captured the recovery of desmosomes, which gradually assembled at the cell periphery after AAV-mediated *PKP2* replacement (Figure 6F and Video S5). Importantly, transduction of AAV2-*PKP2* recovered CV and DD in Hetero-iPSC-CMs after monolayer differentiation (Figure 6G) and significantly restored desmosome assembly in Hetero-tdT-iPSC-CMs (Figure 6H). These data demonstrate the proof of concept for *PKP2* replacement therapy in human cells and suggest that our established isogenic set of iPSC-CMs is a useful model for providing distinct readouts for therapeutic development.

DISCUSSION

In this study, we generated a set of isogenic iPSCs consisting of three clones with precisely adjusted expression of plakophilin-2. Motion vector analysis after monolayer differentiation and the generation of desmosome-imaging isogenic lines using fluorescence tagging of endogenous *DSG2* recapitulated reduced contractility and impaired desmosome assembly under the adjusted dose of plakophilin-2 within 2–4 weeks after differentiation. Several molecular mechanisms, including cell death, excessive lipogenesis, nuclear translocation of γ-catenin, altered calcium signaling, and altered cellular metabolism, have been shown to cause AC due to mutations in *PKP2* by using human iPSC-CMs (Austin et al., 2019; Caspi et al., 2013; Kim et al., 2013; Ma et al., 2013). However, contractile dysfunction due to *PKP2* mutations has not been fully studied in human iPSC-CMs, although ventricular regional dysfunction is one of the modified Task Force diagnostic criteria for AC (Towbin et al., 2019). Under our experimental conditions, reduced contractility caused by *PKP2* haploinsufficiency was elicited in a short period of time, within 2 weeks after differentiation. Because the monolayer protocol confers strong contraction to iPSC-CMs on culture plates soon after differentiation (Burridge et al., 2014; Gintant et al., 2019), continuous tensile overload may facilitate the disease phenotype among isogenic iPSC-CMs. By contrast, common pathological phenotypes, including cell death and lipid accumulation, were recapitulated in NHEJ-iPSC-CMs, which lack plakophilin-2 expression, but not in Hetero-iPSC-CMs, which have a one-half dose of plakophilin-2, within 4 weeks. Because recapitulation of cell death and lipid accumulation caused by *PKP2* mutation requires long-term culture (∼2 months) to promote maturation of iPSC-CMs (Caspi et al., 2013; Kim et al., 2013; Ma et al., 2013), these phenotypes may not be suitable as readouts for early-phase evaluation.

(B) NHEJ-iPSC-CMs transduced with AAV2 encoding FLAG-tagged *PKP2* were fixed and immunostained with the indicated antibodies 5 days after transduction. Areas enclosed within white squares in the left are enlarged in the panels on the right. Scale bar: 50 μm.
(C) Monolayer contracting NHEJ-iPSC-CMs cultured in 12-well plates at day 10 after differentiation were transduced with approximately 1.0×10^4 vg/cell of AAV2-*EGFP* or AAV2-*PKP2*. Fourteen days after transduction, EGFP expression in contracting NHEJ-iPSC-CMs was observed through fluorescence microscopy. Scale bar: 200 μm.
(D) NHEJ-iPSC-CMs were treated as described in (C). Whole-cell lysates were extracted from iPSC-CMs 14 days after transduction and analyzed by western blotting using the indicated antibodies.
(E) Bright-field images and excitation propagation were detected by motion vectors in NHEJ-iPSC-CMs 14 days after transduction, either with AAV2-*EGFP* or with AAV2-*PKP2*. Color range from blue to red represents motion velocity from 0 to 30 μm/s, respectively. Scale bar: 100 μm. CV and DD in iPSC-CMs were calculated using motion vector analysis (Mann-Whitney test, number of analyzed ROIs, AAV2-*EGFP*: 72, AAV2-*PKP2*: 72). Data were collected from three independent experiments.
(F) Sequential merged images of bright-field and tdTomato fluorescence after AAV2-*PKP2* transduction in NHEJ-tdT-iPSC-CMs. Scale bar: 50 μm.
(G) Hetero-iPSC-CMs were treated as described (C). CV and DD in iPSC-CMs were calculated using motion vector analysis (Mann-Whitney test, number of analyzed ROIs, AAV2-*EGFP*: 72, AAV2-*PKP2*: 72). Data were collected from three independent experiments.
(H) Hetero-tdT-iPSC-CMs at day 10 after differentiation were replated and transduced with AAV2-*EGFP* or AAV2-*PKP2*. Fourteen days after transduction; desmosome area was assessed using live-cell high-content imaging. Relative desmosome area of each fluorescence signal in Hetero-iPSC-CMs transduced with AAV2-*PKP2* were normalized to those with AAV2-*EGFP* (Mann-Whitney test, n = 64 images in each iPSC-CM from four independent experiments).

Loss of plakophilin-2 deficiency led to reduced expression of desmosomal cadherins at the cellular periphery in NHEJ-iPSC-CMs. By contrast, plakophilin-2 haploinsufficiency in Hetero-iPSC-CMs did not significantly affect the expression or localization of desmosomal proteins in Hetero-iPSC-CMs compared with isogenic HDR-iPSC-CMs, which is consistent with findings that heterozygous *Pkp2* knockout mice do not exhibit significant morphological abnormalities or differences in expression of intercalated disc proteins, including connexin 43, N-cadherin, and plakoglobin (Cerrone et al., 2012). In this study, immunostaining with desmoglein-2 or desmocollin-2 and quantitative analysis using high-content imaging revealed that a one-half dose reduction of plakophilin-2 decreased desmosome assembly at the periphery of iPSC-CMs. These data indicate that among outer dense plaque proteins, desmosomal cadherins are the most prone to instability due to decreased plakophilin-2 expression. We recently reported a case of desmoglein-2-deficient cardiomyopathy caused by a rare homozygous stop-gain mutation and established isogenic iPSC-CMs from the patient lacking desmoglein-2 (Shiba et al., 2021). Although the loss of desmoglein-2 expression significantly decreased contractile function in three-dimensional tissues (Li et al., 2020), desmoglein-2 deficiency did not significantly affect the differentiation efficiency or morphology of iPSC-CMs. These data prompted us to choose *DSG2* as a molecular marker for real-time desmosome imaging in our isogenic iPSCs. Although the copy number of mRNA transcribed from the knockin allele was lower than that in the normal allele, the isogenic clones carrying the identical knockin *DSG2* alleles allowed a relative comparison of desmosome dynamics under the adjusted dose of plakophilin-2 expression.

AAV2-mediated gene replacement of *PKP2* restored desmosomal proteins and suppressed contractile dysfunction in both NHEJ- and Hetero-iPSC-CMs. The recovery of desmosomes after AAV2-mediated gene replacement was sequentially captured using the desmosome-imaging isogenic lines. These findings provide proof of the therapeutic concept in human cardiomyocytes but may not be directly applied to clinical settings, as most clinically identified mutations in *PKP2* are heterozygous and disease is late onset (Awad et al., 2008; Calkins et al., 2017; Ohno et al., 2013; van Tintelen et al., 2006). However, compound or digenic heterozygosity of desmosome genes, including *PKP2*, is not rare, and patients with combined mutations present with a more severe phenotype (Chen et al., 2019; Gandjbakhch et al., 2018; Rigato et al., 2013). A recent large-cohort analysis using high-throughput sequence analysis has highlighted the high levels of *PKP2* mutations in patients diagnosed with dilated cardiomyopathy (Haas et al., 2015). These findings suggest that *PKP2* haploinsufficiency may develop into severe biventricular heart failure when combined with other pathogenic mutations or other exogenous environmental factors. Furthermore, homozygous deletion of *PKP2* causes untreatable fetal heart failure with left-ventricular non-compaction (Ramond et al., 2017). Although early-onset disease with severe manifestations is rare, mouse myocardia with *Pkp2* mutations are affected by structural injury due to exercise load or environmental stress (Cruz et al., 2015; van Opbergen et al., 2019). This highlights the need for unconventional therapeutic approaches to prevent disease progression. The isogenic cells that we established represent a human disease model that recapitulates reduced contractility and impaired desmosome assembly and provides a convenient cellular platform for therapeutic screening to test upstream molecular targets.

EXPERIMENTAL PROCEDURES

Details are provided in the supplemental experimental procedures.

Human samples

The use of patient-derived samples and genomic analysis was approved by the Ethics Committee of Osaka University Hospital, and written informed consent was obtained from all patients. This study conforms to the ethical guidelines for medical and health research involving human participants in Japan and all principles outlined by the Declaration of Helsinki.

Transfection of plasmids into human iPSCs and selection of targeted clones

Plasmid constructs for genome editing were transfected into iPSCs, and targeted clones were selected as described (Higo et al., 2021; Li et al., 2015).

Motion vector analysis

As described, cell motion profiles of cardiomyocytes differentiated from iPSCs were acquired using the Cell Motion Imaging System (SI8000, SONY) (Hayakawa et al., 2014; Ito et al., 2019).

Data and code availability

The data that support the findings of this study are available from the corresponding author upon reasonable request.

SUPPLEMENTAL INFORMATION

Supplemental information can be found online at https://doi.org/10.1016/j.stemcr.2021.12.016.

AUTHOR CONTRIBUTIONS

Conceptualization: H.I., S.N., and S.H.; Methodology: H.I., S.N., S.H., L.J., and L.L.; Investigation: H.I., S.N., S.H., M.S., Y.K., T.K., S.K., T.T., S.O., Y.I, S.Y., M.T., and E.I.; Software: T.T.; Writing: original draft, H.I., S.N., and S.H.; Writing:review & editing, S.T., S.M., Y.S., S.Hikoso, and Y.S.; Funding Acquisition: S.H., S.M., S. Hikoso, and Y.S.; Supervision: S.M. and Y.S.

CONFLICT OF INTEREST

The Department of Medical Therapeutics for Heart Failure is a Joint Research Department with TOA EIYO Pharmaceutical Company.

ACKNOWLEDGMENTS

We thank M. Moriyasu for technical assistance. This work was supported by JSPS KAKENHI grant numbers 18K08069, 18K19543, 19K08489, 20K21602, and 21H02915, the Japan Agency for Medical Research and Development (19bm0804008h0003), the Cell Science Research Foundation, a grant for Basic Research of the Japanese Circulation Society, SENSHIN Medical Research Foundation, Daiichi Sankyo, and Mitsubishi Tanabe Pharma. The Department of Medical Therapeutics for Heart Failure was an endowment department supported by Actelion Pharmaceuticals Japan (2015–2020). This study was supported by the Center of Medical Innovation and Translational Research and the Center for Medical Research and Education at the Graduate School of Medicine, Osaka University. S.N. was supported by the Osaka University Medical Doctor Scientist Training Program.

Received: June 5, 2020
Revised: December 20, 2021
Accepted: December 21, 2021
Published: January 20, 2022

REFERENCES

Al-Jassar, C., Bikker, H., Overduin, M., and Chidgey, M. (2013). Mechanistic basis of desmosome-targeted diseases. J. Mol. Biol. *425*, 4006–4022.

Austin, K.M., Trembley, M.A., Chandler, S.F., Sanders, S.P., Saffitz, J.E., Abrams, D.J., and Pu, W.T. (2019). Molecular mechanisms of arrhythmogenic cardiomyopathy. Nat. Rev. Cardiol. *16*, 519–537.

Awad, M.M., Calkins, H., and Judge, D.P. (2008). Mechanisms of disease: molecular genetics of arrhythmogenic right ventricular dysplasia/cardiomyopathy. Nat. Clin. Pract. Cardiovasc. Med. *5*, 258–267.

Burridge, P.W., Matsa, E., Shukla, P., Lin, Z.C., Churko, J.M., Ebert, A.D., Lan, F., Diecke, S., Huber, B., Mordwinkin, N.M., et al. (2014). Chemically defined generation of human cardiomyocytes. Nat. Methods *11*, 855–860.

Calkins, H., Corrado, D., and Marcus, F. (2017). Risk stratification in arrhythmogenic right ventricular cardiomyopathy. Circulation *136*, 2068–2082.

Caspi, O., Huber, I., Gepstein, A., Arbel, G., Maizels, L., Boulos, M., and Gepstein, L. (2013). Modeling of arrhythmogenic right ventricular cardiomyopathy with human induced pluripotent stem cells. Circ. Cardiovasc. Genet. *6*, 557–568.

Cerrone, M., Noorman, M., Lin, X., Chkourko, H., Liang, F.X., van der Nagel, R., Hund, T., Birchmeier, W., Mohler, P., van Veen, T.A., et al. (2012). Sodium current deficit and arrhythmogenesis in a murine model of plakophilin-2 haploinsufficiency. Cardiovasc. Res. *95*, 460–468.

Chen, K., Rao, M., Guo, G., Duru, F., Chen, L., Chen, X., Song, J., and Hu, S. (2019). Recessive variants in plakophilin-2 contributes to early-onset arrhythmogenic cardiomyopathy with severe heart failure. Europace *21*, 970–977.

Cruz, F.M., Sanz-Rosa, D., Roche-Molina, M., Garcia-Prieto, J., Garcia-Ruiz, J.M., Pizarro, G., Jimenez-Borreguero, L.J., Torres, M., Bernad, A., Ruiz-Cabello, J., et al. (2015). Exercise triggers ARVC phenotype in mice expressing a disease-causing mutated version of human plakophilin-2. J. Am. Coll. Cardiol. *65*, 1438–1450.

Gandjbakhch, E., Redheuil, A., Pousset, F., Charron, P., and Frank, R. (2018). Clinical diagnosis, imaging, and genetics of arrhythmogenic right ventricular cardiomyopathy/dysplasia: JACC state-of-the-art Review. J. Am. Coll. Cardiol. *72*, 784–804.

Gintant, G., Burridge, P., Gepstein, L., Harding, S., Herron, T., Hong, C., Jalife, J., and Wu, J.C. (2019). Use of human induced pluripotent stem cell-derived cardiomyocytes in preclinical cancer drug cardiotoxicity testing: a scientific statement from the American heart association. Circ. Res. *125*, e75–e92.

Grossmann, K.S., Grund, C., Huelsken, J., Behrend, M., Erdmann, B., Franke, W.W., and Birchmeier, W. (2004). Requirement of plakophilin 2 for heart morphogenesis and cardiac junction formation. J. Cell Biol. *167*, 149–160.

Guan, X., Wang, Z., Czerniecki, S., Mack, D., François, V., Blouin, V., Moullier, P., and Childers, M.K. (2015). Use of adeno-associated virus to enrich cardiomyocytes derived from human stem cells. Hum. Gene Ther. Clin. Dev. *26*, 194–201.

Haas, J., Frese, K.S., Peil, B., Kloos, W., Keller, A., Nietsch, R., Feng, Z., Müller, S., Kayvanpour, E., Vogel, B., et al. (2015). Atlas of the clinical genetics of human dilated cardiomyopathy. Eur. Heart J. *36*, 1123–1135a.

Haugaa, K.H., Haland, T.F., Leren, I.S., Saberniak, J., and Edvardsen, T. (2016). Arrhythmogenic right ventricular cardiomyopathy, clinical manifestations, and diagnosis. Europace *18*, 965–972.

Hayakawa, T., Kunihiro, T., Ando, T., Kobayashi, S., Matsui, E., Yada, H., Kanda, Y., Kurokawa, J., and Furukawa, T. (2014). Image-based evaluation of contraction-relaxation kinetics of human-induced pluripotent stem cell-derived cardiomyocytes: correlation and complementarity with extracellular electrophysiology. J. Mol. Cell Cardiol. *77*, 178–191.

Higo, S., Hikoso, S., Miyagawa, S., and Sakata, Y. (2021). Genome editing in human induced pluripotent stem cells (hiPSCs). Methods Mol. Biol. *2320*, 235–245.

Ishizu, T., Higo, S., Masumura, Y., Kohama, Y., Shiba, M., Higo, T., Shibamoto, M., Nakagawa, A., Morimoto, S., Takashima, S., et al. (2017). Targeted genome replacement via homology-directed repair in non-dividing cardiomyocytes. Sci. Rep. *7*, 9363.

Ito, M., Hara, H., Takeda, N., Naito, A.T., Nomura, S., Kondo, M., Hata, Y., Uchiyama, M., Morita, H., and Komuro, I. (2019). Characterization of a small molecule that promotes cell cycle activation of human induced pluripotent stem cell-derived cardiomyocytes. J. Mol. Cell Cardiol. *128*, 90–95.

Kim, C., Wong, J., Wen, J., Wang, S., Wang, C., Spiering, S., Kan, N.G., Forcales, S., Puri, P.L., Leone, T.C., et al. (2013). Studying arrhythmogenic right ventricular dysplasia with patient-specific iPSCs. Nature *494*, 105–110.

Kohama, Y., Higo, S., Masumura, Y., Shiba, M., Kondo, T., Ishizu, T., Higo, T., Nakamura, S., Kameda, S., Tabata, T., et al. (2020). Adeno-

associated virus-mediated gene delivery promotes S-phase entry-independent precise targeted integration in cardiomyocytes. Sci. Rep. *10*, 15348.

Li, H.L., Fujimoto, N., Sasakawa, N., Shirai, S., Ohkame, T., Sakuma, T., Tanaka, M., Amano, N., Watanabe, A., Sakurai, H., et al. (2015). Precise correction of the dystrophin gene in duchenne muscular dystrophy patient induced pluripotent stem cells by TALEN and CRISPR-Cas9. Stem Cell Rep. *4*, 143–154.

Li, J., Zhang, L., Yu, L., Minami, I., Miyagawa, S., Horning, M., Dong, J., Qiao, J., Qu, X., Hua, Y., et al. (2020). Circulating re-entrant waves promote maturation of hiPSC-derived cardiomyocytes in self-organized tissue ring. Commun. Biol. *3*, 122.

Lyon, R.C., Zanella, F., Omens, J.H., and Sheikh, F. (2015). Mechanotransduction in cardiac hypertrophy and failure. Circ. Res. *116*, 1462–1476.

Ma, D., Wei, H., Lu, J., Ho, S., Zhang, G., Sun, X., Oh, Y., Tan, S.H., Ng, M.L., Shim, W., et al. (2013). Generation of patient-specific induced pluripotent stem cell-derived cardiomyocytes as a cellular model of arrhythmogenic right ventricular cardiomyopathy. Eur. Heart J. *34*, 1122–1133.

Marcus, F.I., McKenna, W.J., Sherrill, D., Basso, C., Bauce, B., Bluemke, D.A., Calkins, H., Corrado, D., Cox, M.G., Daubert, J.P., et al. (2010). Diagnosis of arrhythmogenic right ventricular cardiomyopathy/dysplasia: proposed modification of the task force criteria. Circulation *121*, 1533–1541.

Mashiko, D., Fujihara, Y., Satouh, Y., Miyata, H., Isotani, A., and Ikawa, M. (2013). Generation of mutant mice by pronuclear injection of circular plasmid expressing Cas9 and single guided RNA. Sci. Rep. *3*, 3355.

Ohno, S., Nagaoka, I., Fukuyama, M., Kimura, H., Itoh, H., Makiyama, T., Shimizu, A., and Horie, M. (2013). Age-dependent clinical and genetic characteristics in Japanese patients with arrhythmogenic right ventricular cardiomyopathy/dysplasia. Circ. J. *77*, 1534–1542.

Padron-Barthe, L., Dominguez, F., Garcia-Pavia, P., and Lara-Pezzi, E. (2017). Animal models of arrhythmogenic right ventricular cardiomyopathy: what have we learned and where do we go? Insight for therapeutics. Basic Res. Cardiol. *112*, 50.

Ramond, F., Janin, A., Di Filippo, S., Chanavat, V., Chalabreysse, L., Roux-Buisson, N., Sanlaville, D., Touraine, R., and Millat, G. (2017). Homozygous PKP2 deletion associated with neonatal left ventricle noncompaction. Clin. Genet. *91*, 126–130.

Rasmussen, T.B., Nissen, P.H., Palmfeldt, J., Gehmlich, K., Dalager, S., Jensen, U.B., Kim, W.Y., Heickendorff, L., Mølgaard, H., Jensen, H.K., et al. (2014). Truncating plakophilin-2 mutations in arrhythmogenic cardiomyopathy are associated with protein haploinsufficiency in both myocardium and epidermis. Circ. Cardiovasc. Genet. *7*, 230–240.

Rigato, I., Bauce, B., Rampazzo, A., Zorzi, A., Pilichou, K., Mazzotti, E., Migliore, F., Marra, M.P., Lorenzon, A., De Bortoli, M., et al. (2013). Compound and digenic heterozygosity predicts lifetime arrhythmic outcome and sudden cardiac death in desmosomal gene-related arrhythmogenic right ventricular cardiomyopathy. Circ. Cardiovasc. Genet. *6*, 533–542.

Sharma, A., McKeithan, W.L., Serrano, R., Kitani, T., Burridge, P.W., Del Alamo, J.C., Mercola, M., and Wu, J.C. (2018). Use of human induced pluripotent stem cell-derived cardiomyocytes to assess drug cardiotoxicity. Nat. Protoc. *13*, 3018–3041.

Sheikh, F., Ross, R.S., and Chen, J. (2009). Cell-cell connection to cardiac disease. Trends Cardiovasc. Med. *19*, 182–190.

Shiba, M., Higo, S., Kondo, T., Li, J., Liu, L., Ikeda, Y., Kohama, Y., Kameda, S., Tabata, T., Inoue, H., et al. (2021). Phenotypic recapitulation and correction of desmoglein-2-deficient cardiomyopathy using human induced pluripotent stem cell-derived cardiomyocytes. Hum. Mol. Genet. *30*, 1384–1397.

Towbin, J.A., McKenna, W.J., Abrams, D.J., Ackerman, M.J., Calkins, H., Darrieux, F.C.C., Daubert, J.P., de Chillou, C., DePasquale, E.C., Desai, M.Y., et al. (2019). 2019 HRS expert consensus statement on evaluation, risk stratification, and management of arrhythmogenic cardiomyopathy. Heart Rhythm *16*, e301–e372.

van Opbergen, C.J.M., Noorman, M., Pfenniger, A., Copier, J.S., Vermij, S.H., Li, Z., van der Nagel, R., Zhang, M., de Bakker, J.M.T., Glass, A.M., et al. (2019). Plakophilin-2 haploinsufficiency causes calcium handling deficits and modulates the cardiac response towards stress. Int. J. Mol. Sci. *20*, 4076.

van Tintelen, J.P., Entius, M.M., Bhuiyan, Z.A., Jongbloed, R., Wiesfeld, A.C., Wilde, A.A., van der Smagt, J., Boven, L.G., Mannens, M.M., van Langen, I.M., et al. (2006). Plakophilin-2 mutations are the major determinant of familial arrhythmogenic right ventricular dysplasia/cardiomyopathy. Circulation *113*, 1650–1658.

Wu, H., Yang, H., Rhee, J.W., Zhang, J.Z., Lam, C.K., Sallam, K., Chang, A.C.Y., Ma, N., Lee, J., Zhang, H., et al. (2019). Modelling diastolic dysfunction in induced pluripotent stem cell-derived cardiomyocytes from hypertrophic cardiomyopathy patients. Eur. Heart J. *40*, 3685–3695.

Stem Cell Reports, Volume *17*

Supplemental Information

Modeling reduced contractility and impaired desmosome assembly due to plakophilin-2 deficiency using isogenic iPS cell-derived cardiomyocytes

Hiroyuki Inoue, Satoki Nakamura, Shuichiro Higo, Mikio Shiba, Yasuaki Kohama, Takumi Kondo, Satoshi Kameda, Tomoka Tabata, Shota Okuno, Yoshihiko Ikeda, Junjun Li, Li Liu, Satoru Yamazaki, Maki Takeda, Emiko Ito, Seiji Takashima, Shigeru Miyagawa, Yoshiki Sawa, Shungo Hikoso, and Yasushi Sakata

Supplemental Information

Modeling Reduced Contractility and Impaired Desmosome Assembly due to Plakophilin-2 Insufficiency using Isogenic Induced Pluripotent Stem Cell-Derived Cardiomyocytes

Hiroyuki Inoue[1,#], Satoki Nakamura[2,#], Shuichiro Higo[3,*], Mikio Shiba[1], Yasuaki Kohama[4], Takumi Kondo[1], Satoshi Kameda[1], Tomoka Tabata[1], Shota Okuno[1], Yoshihiko Ikeda[5] , Junjun Li[6,7], Li Liu[6,7], Satoru Yamazaki[8], Maki Takeda[6], Emiko Ito[6], Seiji Takashima[9], Shigeru Miyagawa[6], Yoshiki Sawa[6], Shungo Hikoso[1], Yasushi Sakata[1]

[1] Department of Cardiovascular Medicine, Osaka University Graduate School of Medicine, Suita, Osaka, 565-0871, Japan

[2] Osaka Police Hospital, Osaka 543-0035, Japan

[3] Department of Medical Therapeutics for Heart Failure, Osaka University Graduate School of Medicine, Suita, Osaka, 565-0871, Japan

[4] National Hospital Organization, Osaka-Minami Medical Center, Kawachinagano, Osaka 586-8512, Japan

[5] Department of Pathology, National Cerebral and Cardiovascular Center, Suita, Osaka 564-8565, Japan

[6] Department of Cardiovascular Surgery, Osaka University Graduate School of Medicine, Suita, Osaka 565-0871, Japan

[7] Department of Design for Tissue Regeneration, Osaka University Graduate School of Medicine, Suita,

Osaka 565-0871, Japan
[8] Department of Molecular Pharmacology, National Cerebral and Cardiovascular Center, Suita, Osaka 564-8565, Japan.
[9] Department of Medical Biochemistry, Osaka University Graduate School of Medicine, Suita, Osaka 565-0871, Japan
# These authors contributed equally to the work.

***Corresponding Author**
Shuichiro Higo, Associate Professor
Medical Therapeutics for Heart Failure, Osaka University Graduate School of Medicine
Address: 2-2 Yamadaoka, Suita, Osaka 565-0871, Japan Tel: +81-6-6879-3298, Fax: +81-6-6879-3299,
E-mail: higo-s@cardiology.med.osaka-u.ac.jp

Supplementary_Figure_1

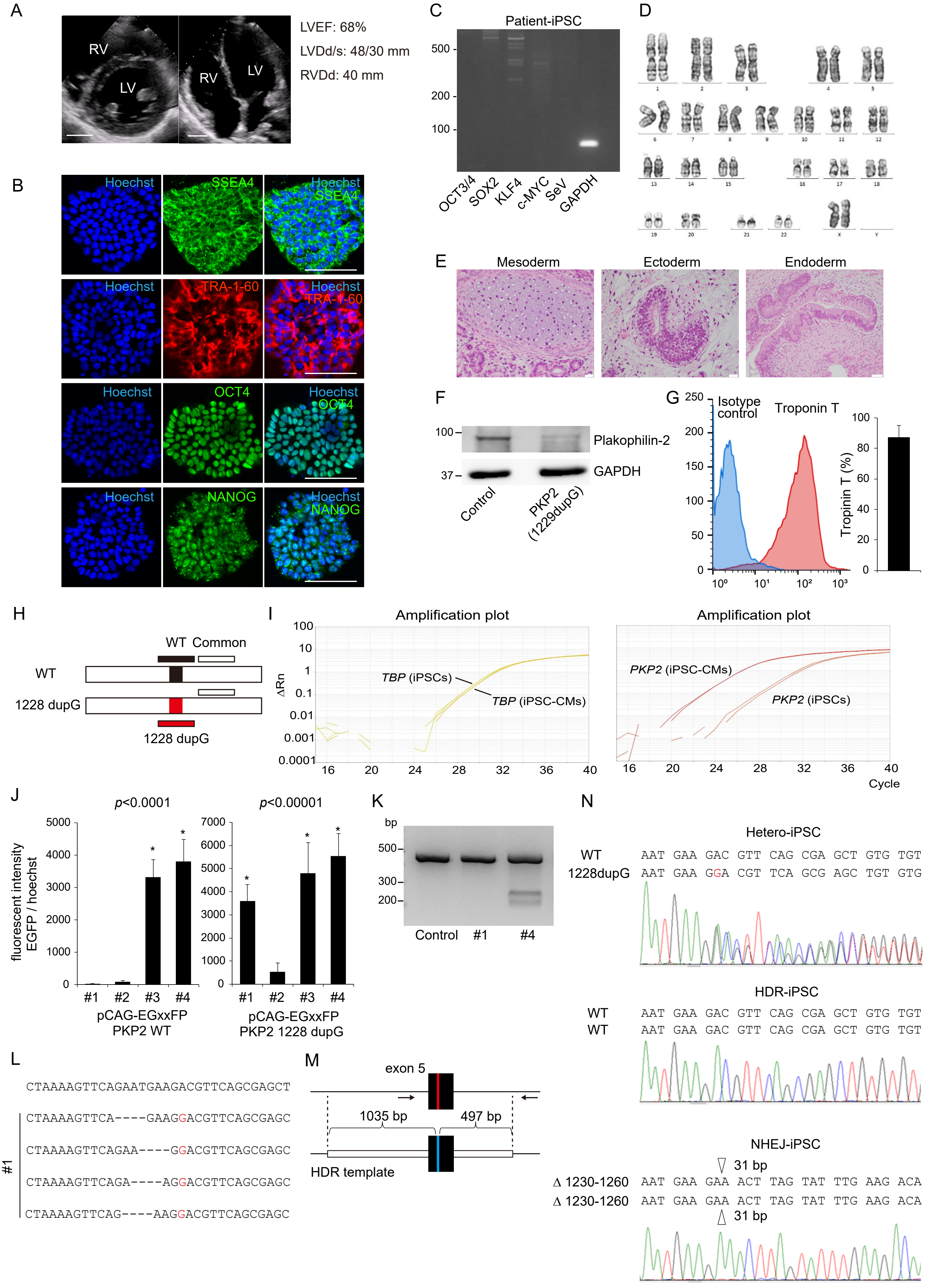

Supplementary_Figure_2

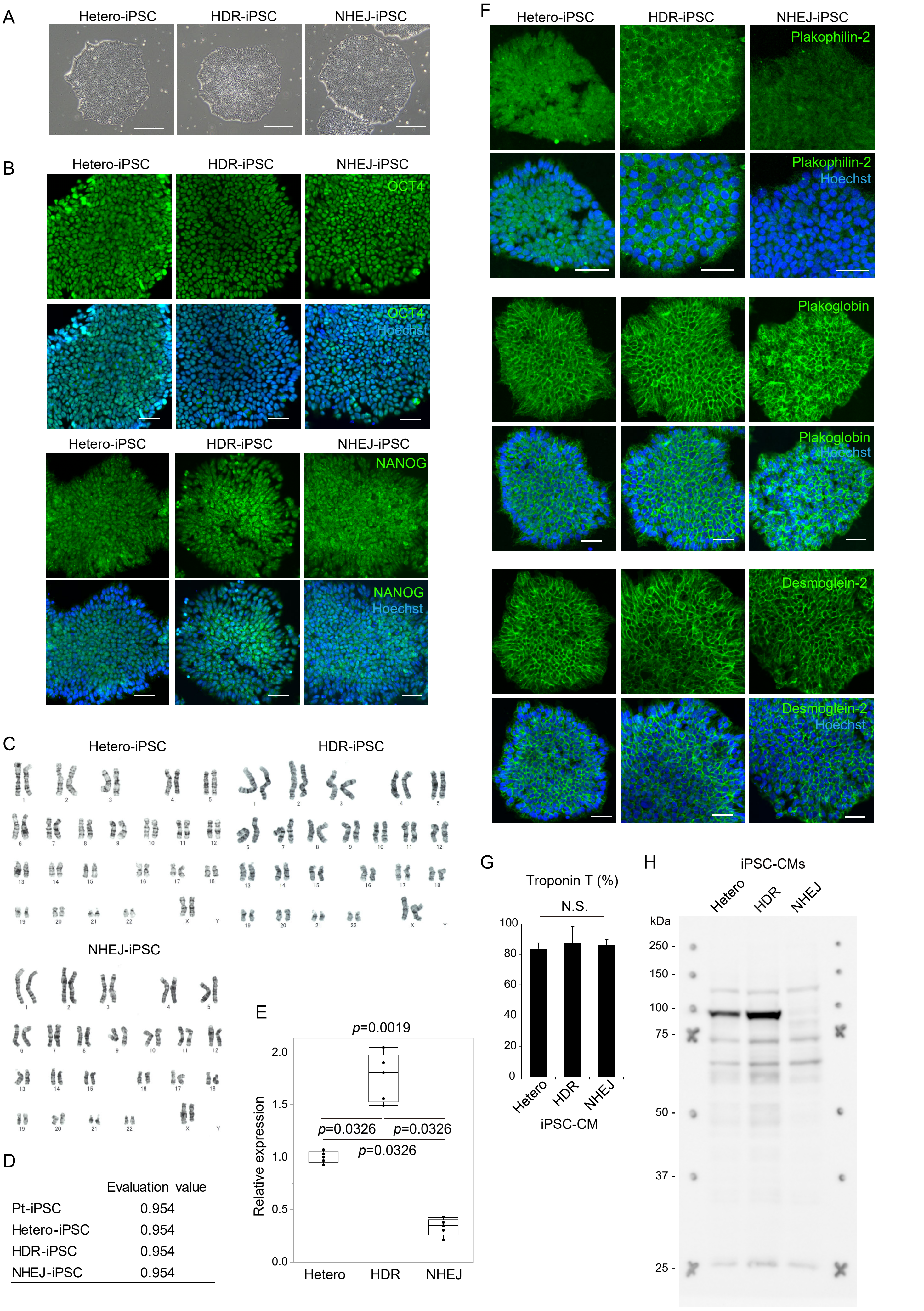

Supplementary_Figure_3

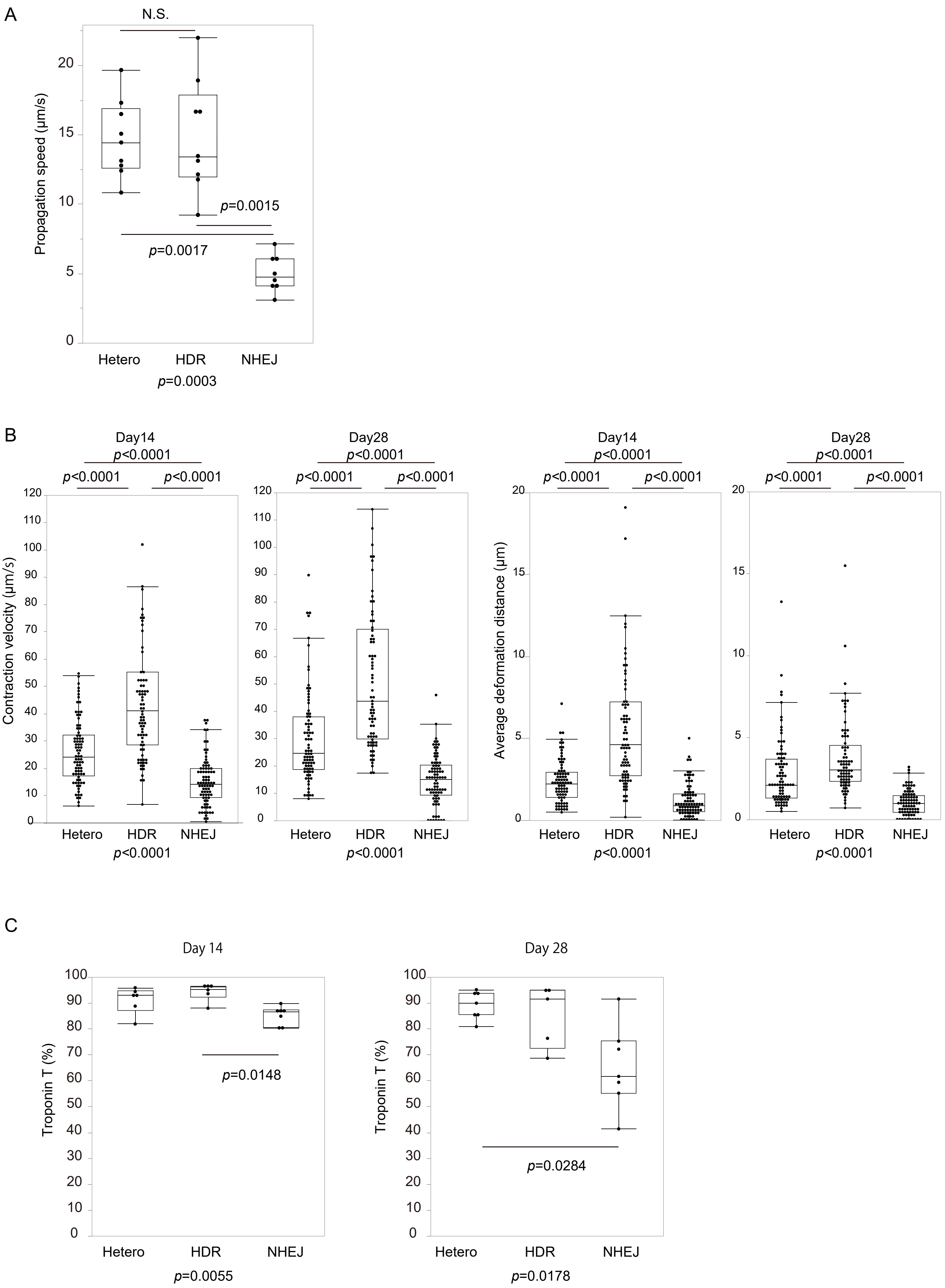

Supplementary_Figure_4

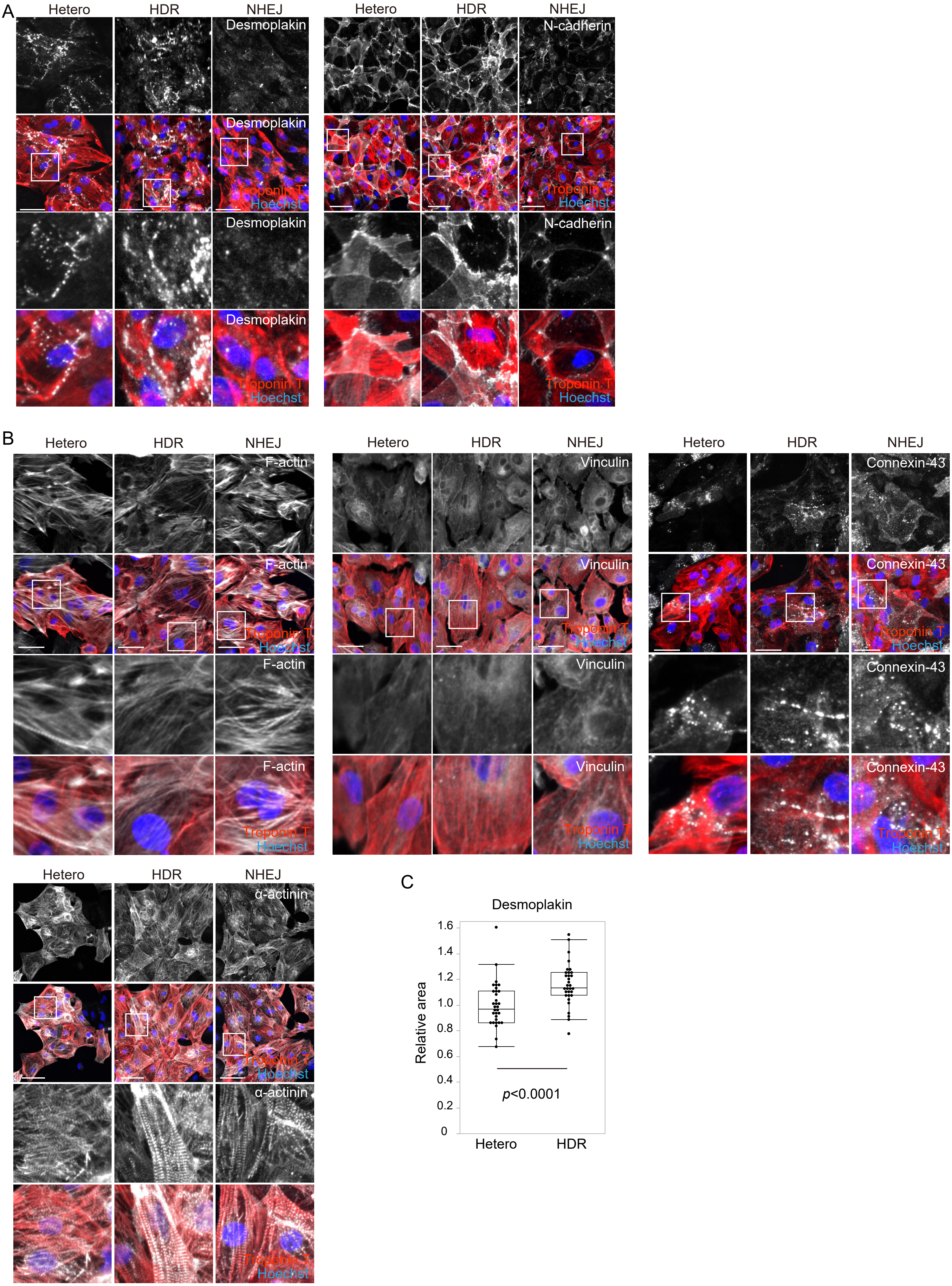

Supplementary_Figure_5

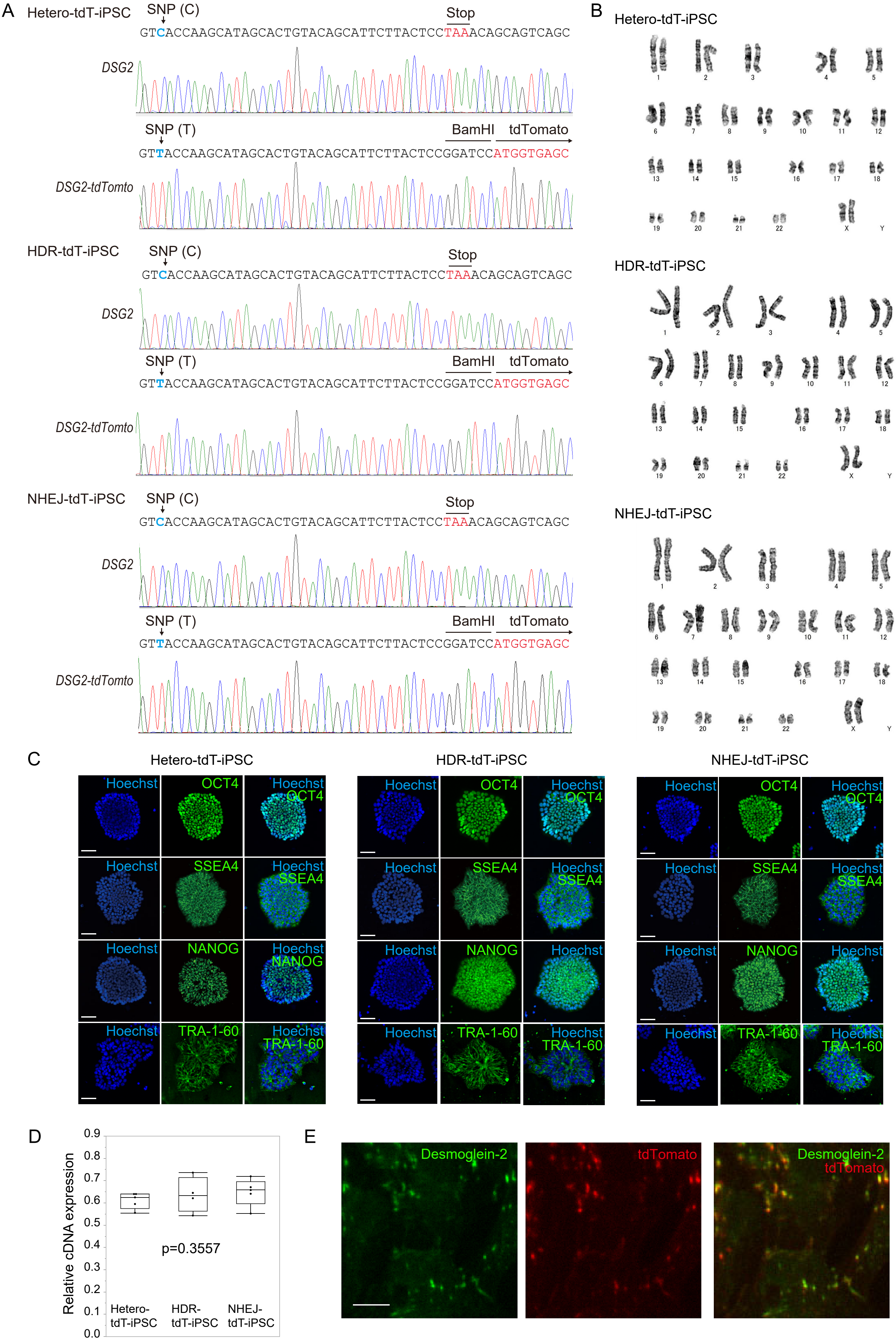

Supplementary_Figure_6

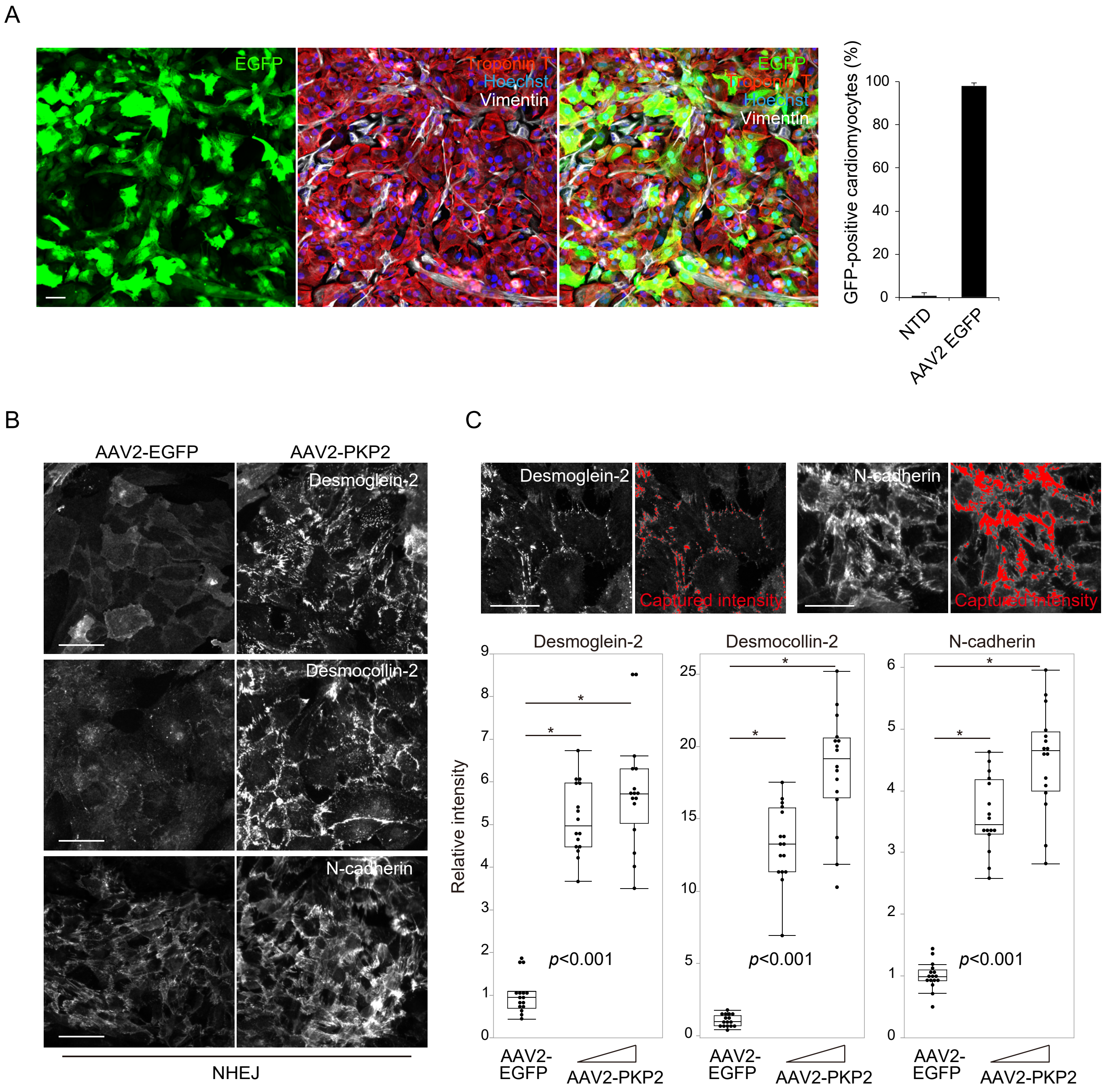

Supplementary Figure Legends

Supplementary Figure 1

A) Parasternal left ventricle short-axis view (left) and four-chamber view (right) of the patient's echocardiogram. LV: left ventricle, RV: right ventricle, EF: ejection fraction, LVDd/s: left ventricular diastolic and systolic diameters, RVDd: right ventricular diastolic diameter. Scale bar: 20 mm.

B) The patient-derived iPSCs were fixed and immunostained with the indicated antibodies against the pluripotent marker proteins. Scale bar: 50 μm.

C) PCR was performed to detect the transgenes generated from residual Sendai viral vectors using the cDNA obtained from patient-derived iPSCs. iPS Transgene/SeV detection primer set (#IDT-DV0301) was used for PCR analysis.

D) Karyotype analysis of the patient-derived iPSCs that were generated.

E) Teratoma formation after subcutaneous injection of iPSCs into NOG mice. Representative H&E staining images of teratoma, including chondrocyte (mesoderm), neuron (ectoderm) and enteron (endoderm) are shown. Scale bars in Mesoderm and Ectoderm: 20 μm. Scale bar in Endoderm: 50 μm.

F) Whole cell lysates were extracted from iPSCs generated from the healthy control or the patient with the *PKP2* mutation and were analyzed by western blotting, using the indicated antibodies.

G) Representative result of FACS analysis in iPSC-CMs after monolayer differentiation. The proportion of troponin T-positive cells were calculated (three independent experiments, mean ± SD).

H) Common qPCR probe that detects both WT and 1228 dupG transcripts and the specific probes that

detect either the WT or 1228 dupG transcript of *PKP2* were designed.

I) Total RNA was extracted from both the patient-derived iPSCs and iPSC-CMs and reverse transcribed to generate cDNA. Quantitative real-time PCR was conducted using the probes targeting *PKP2* (assay ID: qHsaCIP0027871) or *TBP* (assay ID: dHsaCPE5058363). Representative amplification plots are shown. The expression level of *TBP* was used as internal control because the cycle threshold (Ct) value of *TBP* estimated by quantitative real-time PCR was similar to that of *PKP2* in iPSCs, and the Ct value of *TBP* was comparable between iPSCs and iPSC-CMs.

J) The cleaving activities of each gRNA were evaluated using a single strand annealing assay (ANOVA followed by post hoc test (Tukey-Kramer test), *: $p < 0.001$ vs. #2 in WT, *: $p < 0.001$ vs. #2 in 1,228 dupG, five independent experiments, mean ± SD). gRNA #1 specifically cleaved the mutated sequence that contained 1228 dupG but not cleaved WT sequence. gRNA #4 had the highest activity to cleave both WT and 1228 dupG sequence.

K) The cleaving activities of gRNA #1 and #4 targeting the endogenous *PKP2* locus in HEK293T cells were evaluated using the Cel-I assay. gRNA #4 efficiently cleaved the endogenous targeted genomic sequence of *PKP2*.

L) Patient-derived iPSCs were transfected with pX459 encoding gRNA #1. After puromycin selection, genomic DNA was extracted from the iPSC clones. The targeted sequence of *PKP2* was amplified by PCR and cloned into a plasmid vector and sequenced. Representative data from the four individual clones with NHEJ are shown; 1228 dupG is highlighted in red. Genomic cleavages were specifically

introduced into the mutated locus containing the 1228 dupG sequence.

M) Design of HDR repair template, consisting of 1035-bp 5′-terminal and 497-bp 3′-terminal homology arms corresponding to the genomic sequence around exon 5 of *PKP2*. Arrows indicate the positions of PCR primers located inside and outside of the homology arms for sequence analysis.

N) Direct Sanger sequence analysis of the *PKP2* locus using genomic DNA obtained from the isogenic iPS clones that were generated. The duplicated 1228 G sequence is highlighted in red in Hetero sequence. In the NHEJ clone, deletion of the 31 bp sequences was homozygous and occurred in both alleles.

Supplementary Figure 2

A) Bright field images of the isogenic iPSC clones. Scale bar: 250 μm.

B) The isogenic iPS clones cultured in 96-well plates were fixed and immunostained with the indicated antibodies. The nuclei were stained with Hoechst. Bar: 50 μm.

C) Karyotype analysis of the generated isogenic Hetero-, HDR-, and NHEJ-iPSCs.

D) Results of short tandem repeat analysis using genomic DNA obtained from patient-derived iPSCs (Pt-iPSCs), Hetero-, HDR-, and NHEJ-iPSCs. Evaluation value was calculated as the value where (number of coincidental peaks) x 2/total number of peaks in reference sample (genomic DNA obtained from peripheral blood mononuclear cells) + total number of peaks in iPSC sample.

E) Total RNA was extracted both from Hetero-, HDR- and NHEJ-iPSCs. Quantitative real-time PCR was

performed using the common probe that contains the primers amplifying 1517 – 1640 of *PKP2* mRNA (assay ID: qHsaCIP0027871). Obtained data were normalized by the expression levels of *TBP*. Relative expression levels normalized by the value of Hetero-iPSCs are shown (five independent experiments, Kruskal–Wallis test followed by Steel-Dwass test).

F) The isogenic iPS clones cultured in 96-well plates were fixed and immunostained with the indicated antibodies. The nuclei were stained with Hoechst. Bar: 50 μm.

G) Ten days after cardiomyocyte differentiation of the isogenic iPSCs, the proportion of troponin T-positive cells was calculated by FACS analysis (ANOVA, $p = 0.7397$, means ± SD, four independent experiments).

H) Whole cell lysates were extracted from each iPSC-CMs and analyzed by western blot using anti-plakophilin-2 antibody.

Supplementary Figure 3

A) Propagation speed was quantitatively evaluated in Hetero-, HDR- and NHEJ-iPSC-CMs on day 28 using isoclone map data obtained from SI8000 motion analyzer (Kruskal–Wallis test followed by Dunn's test. Number of analyzed images for Hetero-iPSC-CMs: 9, HDR-iPSC-CMs: 9, NHEJ-iPSC-CMs: 8 on day 28, data were collected from three independent experiments).

B) Contraction velocity (CV) and deformation distance (DD) in HDR- and NHEJ-iPSC-CMs on days 14 and 28 under electrical pacing at 1.5 Hz were analyzed using motion vector analysis (Kruskal–Wallis

test followed by Steel-Dwass test). Number of analyzed ROI for all samples were 81. Data were collected from three independent experiments.

C) The proportion of troponin T-positive cells was calculated by FACS analysis. The proportion of troponin T-positive cells was 91.0% vs. 94.0% vs. 85.1% (Hetero- vs. HDR- vs. NHEJ-iPSC-CMs) on day 14, and 88.9% vs. 85.0% vs. 65.0% (Hetero- vs. HDR- vs. NHEJ-iPSC-CMs) on day 28, respectively (Kruskal-Wallis test followed by Steel-Dwass test). Data were collected from six to seven (day 14) and five to seven (day28) independent experiments.

Supplementary Figure 4

A) Hetero-, HDR- and NHEJ-iPSC-CMs were re-plated into 96-well plates at day 10 after differentiation and were subsequently fixed and immunostained at day 14 with the indicated antibodies. Scale bar: 50 μm. High magnification images of the area in the white square are shown in the lower panels.

B) Hetero-, HDR- and NHEJ-iPSC-CMs were re-plated into 96-well plates at day 10 after differentiation and were subsequently fixed and immunostained at day 14 with Alexa Fluor 568 Phalloidin for F-actin staining or the indicated antibodies. Scale bar: 50 μm. High magnification images of the area in the white square are shown in the lower panels.

C) Relative desmosome area of each fluorescent signal in HDR-iPSC-CMs was normalized to that in Hetero-iPSC-CMs (Mann–Whitney test, n = 32 images in each iPSC-CM, data were collected from four independent experiments).

Supplementary Figure 5

A) Sanger sequence analysis of the 3'-terminus of *DSG2* in Hetero-, HDR- and NHEJ-tdT-iPSCs. The upper sequence indicates the allele containing SNP: C and intact 3'-terminus of *DSG2*. The lower sequence indicates the knockin allele containing SNP: T and 3'-terminus of *DSG2* linked to tdToamto sequence.

B) Karyotype analysis of Hetero-, HDR-, NHEJ-tdT-iPSCs.

C) The isogenic iPS clones cultured in 96-well plates were fixed and immunostained with the indicated antibodies. The nuclei were stained with Hoechst. Bar: 50 μm.

D) Relative expression of *DSG2-tdTomato* transcripts normalized by WT *DSG2* transcripts evaluated by ddPCR using cDNA obtained from isogenic tdT-iPSCs (Kruskal-Wallis test, four to five independent experiments).

E) HDR-tdT-iPSC-CMs were fixed and immunostained with anti-desmogelin-2 antibody at day 14 after differentiation. Scale bar: 10 μm.

Supplementary Figure 6

A) NHEJ iPSC-CMs transduced with AAV2-*EGFP* (1.0×10^4 vg/cell) were fixed and immunostained with the indicated antibodies 5 days after transduction. Non-cardiomyocytes were stained with an antibody against vimentin. Scale bar: 50 μm. The proportion of GFP-positive cardiomyocytes were

quantitatively analyzed using high-content imaging (n = 25 images, mean ± SD, data were collected from three independent experiments). NTD indicates the non-transduced control.

B) NHEJ iPSC-CMs transduced with AAV2 encoding *EGFP* or FLAG-tagged *PKP2* were fixed and immunostained with the indicated antibodies 5 days after transduction. Scale bar: 50 µm.

C) The images shown in (B) were quantitatively analyzed by high-content imaging. The upper panels show the raw immunostained images and the captured intensity images detected by high-content imaging. The relative intensities of each fluorescent signal in iPSC-CMs transduced by AAV2-*PKP2* were normalized by those in iPSC-CMs transduced by AAV2-*EGFP* (Kruskal-Wallis test followed by Steel-Dwass test, n = 16 images in each sample, $^{*}p < 0.0001$, data were collected from three independent experiments). 1.0×10^4 viral genomes (vg)/cell of AAV2-*EGFP* and 1.0 or 2.0×10^4 vg/cell of AAV2-*PKP2* were used for transduction.

Supplementary Video 1

Sequential observation of the monolayer iPSC-CMs using motion vector analysis. Bright field images of the fixed positions at specific coordinates of NHEJ-iPSC-CMs at day 14, 18 and 28 are shown.

Supplementary Video 2

Sequential observation of the monolayer of NHEJ-iPSC-CMs using motion vector analysis. Color maps

representing excitation propagation (1/4 speed) of the fixed positions at specific coordinates of NHEJ-iPSC-CMs at days 14 and 19 are shown. At day 14, continuous downward excitation propagation was observed in NHEJ-iPSC-CMs. At day 19, intermittent excitation propagation (2:1 conduction block) in the same fiber structure was observed.

Supplementary Video 3

Supplementary Video 4

Sequential observation of the monolayer iPSC-CMs using motion vector analysis. Bright field images of the fixed positions at specific coordinates of Hetero- (Video 3) and HDR- (Video 4) iPSC-CMs at day 14, 18 and 28 are shown.

Supplementary Video 5

Continuous observation of the restoration process of desmosome assembly in NHEJ-tdT-iPSC-CMs transduced with AAV2-*PKP2*. Images were captured every 30 minutes from 1 to 4 day after the transduction.

Supplementary Table

Oligo DNA	sequence (5'-3')	Application
gRNA against human PKP2 #1	CTCCTAAAAGTTCAGAATGA	sgRNA sequence targeting exon 5 in PKP2
gRNA against human PKP2 #2	GTCCTTCATTCTGAACTTTT	sgRNA sequence targeting exon 5 in PKP2
gRNA against human PKP2 #3	GACGTTCAGCGAGCTGTGTG	sgRNA sequence targeting exon 5 in PKP2
gRNA against human PKP2 #4	ACGTTCAGCGAGCTGTGTGT	sgRNA sequence targeting exon 5 in PKP2
gRNA against human DSG2	TTAAACTCTGGGTCAGTTTG	sgRNA sequence targeting exon 15 in DSG2
Forward primer to amplify HDR template (PKP2)	CCCATTCCTGGTTGTGCCCT	cloning of HDR template (5'- and 3'-terminal homology arms of PKP2)
Reverse primer to amplify HDR template (PKP2)	ATTAGCCAGGTGTGGTAGCA	cloning of HDR template (5'- and 3'-terminal homology arms of PKP2)
Forward primer to amplify genomic sequence around 1228 dupG in PKP2 (BamHI recognition site is added for subcloning)	ggatccACAAGAGCCTCAGTTGTGCT	cloning for pCAG-EGxxFP vector, Cel-I assay to detect genomic cleavage at PKP2 locus<br>PCR for sequence confirmation after sibselection
Reverse primer to amplify genomic sequence around 1228 dupG in PKP2 (EcoRI recognition site is added for subcloning)	gaattcAGGCATCTGGCTGGGGTGCAA	cloning for pCAG-EGxxFP vector, Cel-I assay to detect genomic cleavage at PKP2 locus
Reverse primer to amplify genomic sequence around 1228 dupG in PKP2	CAGTGGCTCATGGCTCATGC	PCR for sequence confirmation after sibselection
Forward primer to amplify genomic sequence around tdTomato knockin site (SphI recognition site is added for subcloning)	gcatgcTCTTTGCAAGAAGCCAATGC	cloning of HDR template (5'- terminal homology arm of DSG2-tdTomato)
Reverse primer to amplify genomic sequence around tdTomato knockin site (BamHI recognition site is added for subcloning)	ggatccGGAGTAAGAATGCTGTACAGT	cloning of HDR template (5'- terminal homology arm of DSG2-tdTomato)
Forward primer to amplify genomic sequence around tdTomato knockin site (HpaI recognition site is added for subcloning)	gttaacTAAACAGCAGTCAGCCACAAACT	cloning of HDR template (3'- terminal homology arm of DSG2-tdTomato)
Reverse primer to amplify genomic sequence around tdTomato knockin site (AflII recognition site is added for subcloning)	cttaagCAAACTCCTGACCACAGTTGA	cloning of HDR template (3'- terminal homology arm of DSG2-tdTomato)
Forward primer to amplify genomic sequence to distinguish the tdTomato knocked-in allele	GCAATCCAGTTACCAGATTCCCAC	PCR for sequence confirmation after sibselection
Reverse primer to amplify genomic sequence to distinguish the tdTomato knocked-in allele	GAAAGTCTGCTGGGTGCAGT	PCR for sequence confirmation after sibselection
OCT3/4_Fw	CCCGAAAGAGAAAGCGAACCAG	detection of Sendai virus-mediated transgenes in iPSCs
OCT3/4_Rv	AATGTATCGAAGGTGCTCAA	detection of Sendai virus-mediated transgenes in iPSCs
SOX2_Fw	ACAAGAGAAAAAACATGTATGG	detection of Sendai virus-mediated transgenes in iPSCs
SOX2_Rv	ATGCGCTGGTTCACGCCCGCGCCCAGG	detection of Sendai virus-mediated transgenes in iPSCs
KLF4_Fw	ACAAGAGAAAAAACATGTATGG	detection of Sendai virus-mediated transgenes in iPSCs
KLF4_Rv	CGCGCTGGCAGGGCCGCTGCTCGAC	detection of Sendai virus-mediated transgenes in iPSCs
c-MYC_Fw	TAACTGACTAGCAGGCTTGTCG	detection of Sendai virus-mediated transgenes in iPSCs
c-MYC_Rv	TCCACATACAGTCCTGGATGATGATG	detection of Sendai virus-mediated transgenes in iPSCs
SeV_Fw	GGATCACTAGGTGATATCGAGC	detection of Sendai virus-mediated transgenes in iPSCs
SeV_Rv	ACCAGACAAGAGTTTAAGAGATATGTATC	detection of Sendai virus-mediated transgenes in iPSCs

Probe for ddPCR or qRT-PCR	Assay ID	
PKP2 WT (HEX), 1228 dupG (FAM)	dMDS329472318	ddPCR probe (annealing temperature: 53°C)
PKP2	qHsaCIP0027871	ddPCR probe (annealing temperature: 54°C)
TBP	dHsaCPE5058363	ddPCR probe (annealing temperature: 53°C)

Custom made primers and probes for ddPCR	Sequence (5'-3')	
Forward primer to amplify genomic sequece around SNP of DSG2	CTGGTCATTCTAATTCTAC	ddPCR probe (annealing temperature: 52°C)
Reverse primer to amplify genomic sequece around SNP of DSG2	GGAGTAAGAATGCTGTA	ddPCR probe (annealing temperature: 52°C)
Probe to detect DSG2 SNP:T (HEX)	ACCAGAGTTACCAAGCA	ddPCR probe (annealing temperature: 52°C)
Probe to detect DSG2 SNP:C (FAM)	ACCAGAGTCACCAAGC	ddPCR probe (annealing temperature: 52°C)

Supplemental Experimental Procedures

Reagents and antibodies

Antibodies used in this study

Oct-3/4 (C-10) (Santa Cruz Biotechnology, Dallas, Texas, USA, Cat# sc-5279, RRID: AB_628051, x500 dilution), TRA-1-60 (Merck Millipore, Burlington, Massachusetts, USA, Cat# MAB4360, RRID: AB_2119183, x400 dilution), SSEA-4 (Merck Millipore, Cat# MAB4304, RRID: AB_177629, x200 dilution), Nanog (Abcam, Cambridge, MA, USA, Cat# ab80892, RRID: AB_2150114, x200 dilution), DSG2 (AH12.2) (Santa Cruz Biotechnology, Cat# sc-80663 RRID: AB_2093438, x1000 dilution (immunostaining), x2000 dilution (western blot)), PKP2 (PROGEN, Germany, Cat#651167, x50 dilution (immunostaining)), PKP2 (Abcam Cat# ab151402, x1000 dilution (western blot)), Plakoglobin (Cell Signaling Technology, Tokyo, Japan, Cat# 2309, RRID: AB_823448, x1000 dilution (immunostaining), x2000 dilution (western blot)), Desmocollin-2/3 (7G6) (Thermo Fisher Scientific, Waltham, Massachusetts, USA, Cat#32-6200, RRID:AB_2533090, x1000 dilution (immunostaining), x2000 dilution (western blot)), Desmoplakin (Abcam, Cambridge, MA, USA, Cat# ab16434, RRID:AB_443375, x200 dilution (immunostaining)), Connexin 43 (Cell Signaling Technology, Cat# 3512, RRID: AB_2294590, x2000 dilution), Troponin T (Abcam Cat# ab64623, RRID:AB_1139590, x1000 dilution (immunostaining), x1000 dilution (western blot)), GAPDH (Santa Cruz Biotechnology Cat# sc-47724, RRID: AB_627678, x2000 dilution (western blot)), Sarcomeric Alpha Actinin (EA-53) (Abcam, Cat# ab9465, RRID: AB_307264, x1000 dilution (immunostaining), x2000 dilution (western blot)), Vinculin (Sigma-Aldrich Cat# V9131, RRID:AB_477629,

x1000 dilution (immunostaining)), Vimentin (Abcam Cat# ab24525 RRID:AB_778824, x1000 dilution (immunostaining)), Alexa Fluor™ 568 Phalloidin (Thermo Fisher Scientific, Waltham, Massachusetts, USA, A12380, x2000 dilution (F-actin staining)), BV421 Mouse IgG1, k Isotype Control (BD Bioscience, Tokyo, Japan, Cat# 562438, RRID: AB_2721018, x300 dilution (FACS)), BV421 Mouse Anti-Cardiac Troponin T (BD Bioscience, Cat# 565618, RRID: AB_2739306, x300 dilution (FACS)). Puromycin dihydrochloride (SIGMA, cat# P9620-10ML).

Amplicon sequence analysis

The genomic DNA was extracted from the peripheral blood of the patient using the QIAAmp DNA mini kit (QIAGEN). We prepared the genomic DNA library using the Ion AmpliSeq Library Kit and Ion Ampliseq Cardiovascular Research Panel, which contains 10,430 PCR amplicons covering 404 genes known to harbor mutations affecting cardiovascular function. The sequencing run was conducted using Ion PGM with 318 Chips. Sequencing data were analyzed using TorrentSuite (version 5.2.2, Life Technologies). Variants with a low-quality score less than 30 or with low read depth less than 30 were excluded. Synonymous mutations without amino acid changes were excluded. Variants were classified as benign when they were present in Human Genetic Variation Database (HGVD)(Higasa et al., 2016; Narahara et al., 2014), ESP 6500 database(Fu et al., 2013; Tennessen et al., 2012), 1000 genomes database(Genomes Project et al., 2015), or in the ExAC database(Lek et al., 2016) with an allele frequency more than 1%. The heterozygous frameshift mutation (c.1228 dupG, p.D410fs) in *PKP2* was not reported in either HGVD, ESP 6500, 1000 genomes,

the ExAC database, or the ARVD/C Genetic Variants Database(van der Zwaag et al., 2009). Pathogenic mutations were not detected in other desmosomal (*DSC2, DSG2, JUP*, and *DSP*) or non-desmosomal (*TMEM43, LMNA, DES, CTNNA3, PLN, TGFB3, TTN, SCN5A, CDH2*) genes.

Cell Culture and cardiomyocyte differentiation

HEK293T cells were maintained in high glucose Dulbecco's Modified Eagle Medium (DMEM, Gibco) supplemented with 10% fetal bovine serum (FBS, Gibco) and penicillin, streptomycin, and glutamine (PSG, Gibco). iPSCs were generated from PBMCs from a patient diagnosed with ARVC harboring the 1228 dupG mutation. PBMCs were separated from the peripheral whole blood using Ficoll–Plaque (GE). Reprogramming was conducted using Sendai virus vectors with OCT3/4, SOX2, KLF4, and c-MYC (CytoTune-iPS 2.0 Sendai Reprogramming Kit, Life Technologies). A total of 12 clones were generated from the patient carrying heterozygous 1228 dupG mutation in *PKP2*. Among the generated clones, the two iPS clones with round shape colony, carrying normal karyotype, with expression of pluripotent markers and normal differentiating ability to cardiomyocytes were selected and cryopreserved. To generate isogenic iPSCs, one iPS clone was transfected with the plasmid vectors for genome editing. At least 24 colonies were selected after electroporation and were screened with Sanger sequencing. We selected HDR-, NHEJ- and Hetero-iPSC (as a control) clones with round shape colony, carrying normal karyotype, with expression of pluripotent markers and normal differentiating ability to cardiomyocytes. These iPSC clones (two original iPSCs, HDR-, NHEJ- and Hetero-iPSCs) were deposited to RIKEN BioResource Research Center. iPSCs

were cultured under feeder-free conditions using StemFit AK02N (AJINOMOTO), as described previously (Nakagawa et al., 2014), on a laminin-coated plate. iPSCs were differentiated into iPSC-CMs using a chemically defined protocol, as previously described(Burridge et al., 2014). The culture medium was exchanged for RPMI 1640 medium (ThermoFisher Scientific, USA) with recombinant human albumin (Sigma-Aldrich) and L-ascorbic acid 2-phosphate (Sigma-Aldrich) for differentiation. iPSCs were treated with CHIR99021 (LC Laboratories, USA) (days 0–2), Wnt-C59 (Selleck Chemicals, USA) and XAV-939 (Cayman) (days 2–4). For sequential observation of HDR-, NHEJ-, and Hetero-iPSC-CMs, differentiated monolayer cardiomyocytes were cultured until day 14 in the RPMI medium. The medium was then exchanged to DMEM supplemented with 10% FBS, 1% penicillin/streptomycin, and 2 mM L-Glutamine (PSG, Gibco, Thermo Fisher Scientific) for further analysis. For continuous observation studies of Hetero- and HDR-iPSC-CMs or for immunostaining experiments followed by high-content imaging, differentiated iPSC-CMs were dissociated with 0.25% Trypsin-EDTA (Gibco, USA) and re-plated into 96-well μClear plates (Greiner) (2×10^4 cells/well) precoated with gelatin (Nitta Gelatin) and incubated with DMEM containing serum.

Transfection of the plasmid components

293T cells were transfected with Lipofectamin 3000 (Invitrogen). iPSCs were transfected by electroporation using NEPA 21 electroporator (Poring pulse: pulse voltage 125 V, pulse width 5 ms, pulse interval 50 ms, pulse number, 2. Transfer pulse: pulse voltage 20 V, pulse width 20 ms, pulse interval 50 ms, pulse number,

5). To introduce a homozygous frameshift mutation via NHEJ that mimics the mutated sequence in *PKP2*, a pX459 vector encoding gRNA #4 was transfected into the patient-derived iPSCs. To replace the mutated sequence via HDR, a pX459 vector encoding gRNA #1 combined with the repair template vector were transfected into patient-derived iPSCs.

Teratoma assay

This animal experiment was conducted properly in compliance with the Guidelines for Animal Experiments of Osaka University. For in vivo teratoma assay, we used immune deficient NOD/Shi-scid, IL-2R γnull mice (NOG mice; female, 7-8weeks) were obtained from In-Vivo Science Inc. (Tokyo, Japan). Fifty microliters (1 × 106) of dissociated hiPSCs were injected into the subcutaneously of the back. After 16 weeks of observation, the subcutaneous tissues were extracted. All surgeries and sacrifices were performed under deep anesthesia enough to minimize the animal suffering. Subcutaneous tissues of NOG mice were fixed with 10% buffered formalin and embedded in paraffin. Serial paraffin-embedded sections cut at a thickness of 0.5 μm were deparaffinized in xylene, dehydrated in a graded series of ethanol, and stained with hematoxylin and eosin.

Droplet digital PCR and quantitative real-time PCR

Droplet digital PCR (ddPCR) was performed as previously described(Gu et al., 2017) using QX200 ddPCR system (BIORAD). To specifically detect the transcripts from WT allele or 1228 dupG allele in *PKP2*, HEX-

or FAM-labeled probe was designed (assay ID: dMDS329472318, BIORAD). To detect both WT and 1228 dupG transcripts, the ready-made FAM-labeled probe for *PKP2* (assay ID: qHsaCIP0027871) was used. After the PCR reaction, the generated droplets were detected and analyzed using QX200 droplet reader (BIORAD). TBP was used for internal control (assay ID: dHsaCPE5058363).

Transfection of plasmids into human iPSCs and selection of targeted clones

Plasmid constructs for genome editing were transfected into iPSCs, as described (Higo et al., 2021; Li et al., 2015), with modifications. Briefly, 5 μg of pX459 plasmid was electroporated into 1×10^5 cells using the NEPA 21 electroporator (poring pulse pulse voltage: 125 V, pulse width: 5 ms, pulse number: 2, NEPA GENE). For HDR-mediated genome editing, 5 μg of repair template DNA plasmid (pCR bluntII-TOPO vector) was additionally transfected. Puromycin (0.3 μg/mL) was added within 48 h after electroporation. Three days after transfection, iPSCs were passaged into 35 mm dishes at a density of 200 cells for clonal colony formation. Simultaneously, genomic DNA was extracted, and genome editing results were evaluated by direct sequencing. After iPSC colonies were formed, at least 24 colonies were picked individually and dissociated into single cells in sterile tubes. The cell suspensions were seeded into two 96-well plates for genotyping and cell expansion. Genomic DNA was extracted; target genomic region was amplified using PCR and evaluated by direct sequencing or sequence analysis after cloning into pCR bluntII-TOPO vectors. To obtain targeted single clone iPSCs, cells were passaged into a new culture dish repeatedly for clonal colony formation.

Motion vector analysis

As described, cell motion profiles of cardiomyocytes differentiated from iPSCs were acquired using the Cell Motion Imaging System (SI8000, SONY) (Hayakawa et al., 2014; Ito et al., 2019). Motion videos were recorded using a 4× objective at a frame rate of 150 fps, with a resolution of 1024 × 1024 pixels. Motion videos were obtained from at least three fields from three wells in each isogenic iPSC-CM cultured in 6- or 12-well plates. In each image, motion parameters were calculated from nine regions of interest (ROIs) with 64 × 64 pixels. Data were acquired from at least three independent experiments. During observation, fixed positions, defined as X- and Y-axes, were sequentially observed. Maximum CV, RV, and average DD during the contraction–relaxation process were calculated as the total area under the CV and RV peaks. CV and DD calculated using the motion vector represent contractile function and contractile force, respectively. Color mapping images allowed visualization of motion propagation of contracting iPSC-CMs. In the measurement under electrical pacing, we stimulated cardiomyocytes by an electrical stimulator (MyoPacer EP, Ion Optix, USA) at 1.5 Hz.

Transmission electron microscopy

iPSC-CMs after monolayer differentiation were fixed with 2.5% glutaraldehyde for 2 h. After fixation with 1% osmium tetraoxide for 90 min, the cells were dehydrated through a graded series of ethanol (50–100%) and propylene oxide and embedded in epoxy resin. Ultrathin sections were cut using an ultramicrotome

(Ultracut E; Reichert-Jung, Vienna, Austria) and stained with uranyl acetate and lead citrate. Microstructures of the differentiated isogenic iPSC-CMs were observed using a TEM (H-7650; Hitachi Co., Tokyo, Japan).

Immunofluorescent staining

iPSCs were seeded into 96-well μClear plates (Greiner) at 1,000 cells/well and incubated at 37ºC for colony formation. Differentiated iPSC-CMs were treated with 0.25% Trypsin-EDTA, suspended with DMEM containing 10% FBS, PSG and 10 μM Y-27632 (Wako) and filtered with 100 μm cell strainer (FALCON). Cardiomyocytes were seeded at 10,000 cells/well into 96-well μClear plates precoated with gelatin (Nitta Gelatin). For immunostaining, cells were fixed with 4% paraformaldehyde for 15 min, permeabilized with 0.5% Triton X-100 for 15 min and blocked with 1% BSA for 30 min at room temperature or overnight at 4°C. Primary antibodies were diluted by 1% bovine serum albumin (BSA), added to each well and incubated for 1 h at room temperature or overnight at 4°C. Secondary antibodies conjugated with Alexa Fluor Dyes (Molecular Probe) including Hoechst 33342 for nuclear staining or Alexa 568-conjugated phalloidin (Thermo) were added and incubated for 30 min at room temperature. All images were acquired using the IN Cell Analyzer 6000 (GE healthcare). For high-content imaging analysis for dot-distributed proteins (desmoglein-2, desmocollin-2 and desmoplakin), 9 – 16 nonoverlap images per well in 96-well plates were obtained using a 20×/0.45NA Nikon lens. The dot-distributed immunofluorescent signals were quantitatively analyzed using IN Cell Developer toolbox (version1.9, GE).

Single strand annealing assays

Targeted genomic sequences were cloned into pCAG-EGxxFP vector(Mashiko et al., 2013) encoding tandem truncated EGFP gene with overlapped sequence separated by the cloning site. pCAG-EGxxFP vector with pX459 vector(Ran et al., 2013) encoding SpCas9 and the indicated sgRNAs were transfected into 293T cells pre-seeded in Greiner CELLSTAR 96-well plate (1×10^4 cells/well). Forty-eight hours after transfection, the fluorescent images of EGFP were obtained by high-content image analysis (IN Cell Analyzer 6000, GE), and quantitatively analyzed using IN Cell Developer Toolbox (GE). A total of 36 nonoverlap images (9 images per well) were obtained from each sample in one experiment using a 10×/0.45NA Nikon lens.

Cel-I assay

HEK293T cells were seeded in a 24 well plate (5×10^4 cells/well) one day before transfection. pX459 vector was transfected into HEK293T cells using Lipofectamine 3000 (Life Technologies). Two days after transfection, the medium was exchanged to the medium containing 1.0 μg/mL puromycin to select the cells expressing Cas9. After puromycin selection, genomic DNA was extracted using QIAamp DNA Mini Kit (QIAGEN). Target regions were amplified by PCR (KOD Fx Neo, TOYOBO) as follows: 94°C for 2 min, followed by 33 cycles of 98°C for 10 s, annealing temperature (depending on primer sequences) for 30 s and 68°C for 30 s. Primer sequences are listed in Supplementary Table. After purification of PCR products using QIAquick PCR purification kit (QIAGEN), PCR fragments both from untreated and treated allele were

hybridized to form hetero DNA duplex. Then, hybridized PCR hetero duplexes were enzymatically digested by mismatch-specific endonuclease, Cel-I at 42°C for 60 min (SURVEYOR Mutation Detection Kit) and electrophoresed.

Plasmid construction

gRNA sequences targeting the genomic region surrounding 1228 dupG mutation in *PKP2* were designed using CRISPR Design Tool(Hsu et al., 2013), and cloned into pX459 vector as previously described(Ran et al., 2013). DNA sequences for 5'-terminal and 3'-terminal homology arms surrounding 1228 dupG mutation in *PKP2* gene were amplified from WT genomic DNA, then cloned into pCR bluntII-TOPO vector (Thermo). Full length human *PKP2* coding sequence was subcloned from ORF clone (Dharmacon) into pENTR/D-TOPO vector (Thermo). For expression in cultured cells, *PKP2* sequence was recombined into pcDNA3.1/nV5-DEST (Thermo) using the Gateway system (Invitrogen). To generate N-terminal FLAG-tagged protein, the FLAG epitope (DYKDDDDK) was inserted before the coding sequence by PCR-based mutagenesis. For AAV generation N-terminally FLAG-tagged full length *PKP2* sequence was subcloned into pAAV vector (TaKaRa).

Generation of AAV and transduction in iPSC-CMs

To generate AAV2, HEK293T cells were transfected with pAAV vector encoding N-terminally FLAG-tagged PKP2 or pAAV vector encoding EGFP as control, pHelper vector and pRC2-mi342 Vector (AAVpro Helper

Free System, TaKaRa) using calcium phosphate transfection (CalPhos Mammalian Transfection Kit,TaKaRa). Seventy-two h after transfection, HEK293T cells were detached by addition of 1/80 volume of 0.5M EDTA (pH 8.0), then pelleted via low-speed centrifugation (2000 x g for 10 min). Cell pellet was lysed with AAV Extraction Solution A and centrifuged (9000 x g for 10 min). AAV Extraction Solution B was added to the collected supernatant and stored at -80 °C. Collected AAV generated from HEK293T cells was purified using AAVpro Purification Kit (TaKaRa), and viral titer was calculated using AAV Titration Kit (TaKaRa). For immunostaining or time-lapse imaging, iPSC-CMs around 10 days after differentiation were replated into 96-well plates (2 x 10^4 cells/well) or glass-based dishes (8 x 10^4 cells/well), then transduced with AAV2 at 1.0 – 2.0 x 10^4 vg/cell. For motion vector analysis or western blotting, iPSC-CMs in 12-well plates (2.0 x 10^6 – 1.0 x 10^7 cells/well) around 10 days after differentiation were transduced with AAV2 at approximately 1.0 x 10^4 vg/cell.

RNA extraction, quantitative real-time PCR

Total RNA was extracted using RNeasy mini kit (QIAGEN) and converted to cDNA using high capacity RNA-to cDNA RT kit (Thermo). Quantitative real-time PCR was performed using SYBR green or probe method (THUNDERBIRD SYBR, probe qPCR mix, TOYOBO). All of the samples were processed in duplicate. The level of each transcript was quantified by the threshold cycle (Ct) method using TBP or GAPDH as internal controls. PCR primers and probes used for quantitative PCR are listed in supplementary table.

Western Blotting

For western blotting, cells were washed with cold PBS and directly lysed with SDS buffer (10% SDS, 50mM Tris-HCl (pH7.4), 5mM EDTA). The protein concentration was determined by BCA Protein Assay Kit (Thermo). Lysate samples were mixed with 4 × Laemmli sample buffer (BIORAD) with mercaptoethanol (2.5%). Proteins were separated by SDS-PAGE and transferred to PVDF membrane. Antibodies were diluted by 3% nonfat milk. After blocking with 3% nonfat milk for 1 h, the transferred membrane was incubated with primary antibody at 4°C overnight and with secondary antibody at room temperature for 30 min. The membrane signals were detected by chemiluminescence using ECL or ECL prime reagent (GE). The protein expression level was quantified using ImageQuant TL (GE). The expression levels of each protein were normalized by those of GAPDH.

Statistical Analysis

Normally distributed data were analyzed by ANOVA followed by post hoc test (Tukey-Kramer test) for comparison in more than three groups and were represented as means ± S.D. Data that were not normally distributed were analyzed by Mann-Whitney test for comparison in two groups, or Kruskall-Wallis test followed by post hoc test (Dunn's test or Steel-Dwass test) for comparison in more than three groups and were represented as box plots. The box plot consists of a box ranging from the 25th quantile to the 75th quantile. The horizontal line in each box indicates the median value. The whiskers indicate 1.5 times the

interquartile range with outliers, or minimum and maximum values otherwise. Statistical analysis was conducted with JMP (SAS, Cary, NC) and we considered a p-value of less than 0.05 to be statistically significant.

Reference

Burridge, P.W., Matsa, E., Shukla, P., Lin, Z.C., Churko, J.M., Ebert, A.D., Lan, F., Diecke, S., Huber, B., Mordwinkin, N.M., *et al.* (2014). Chemically defined generation of human cardiomyocytes. Nat Methods *11*, 855-860.

Fu, W., O'Connor, T.D., Jun, G., Kang, H.M., Abecasis, G., Leal, S.M., Gabriel, S., Rieder, M.J., Altshuler, D., Shendure, J., *et al.* (2013). Analysis of 6,515 exomes reveals the recent origin of most human protein-coding variants. Nature *493*, 216-220.

Genomes Project, C., Auton, A., Brooks, L.D., Durbin, R.M., Garrison, E.P., Kang, H.M., Korbel, J.O., Marchini, J.L., McCarthy, S., McVean, G.A., *et al.* (2015). A global reference for human genetic variation. Nature *526*, 68-74.

Gu, M., Shao, N.Y., Sa, S., Li, D., Termglinchan, V., Ameen, M., Karakikes, I., Sosa, G., Grubert, F., Lee, J., *et al.* (2017). Patient-Specific iPSC-Derived Endothelial Cells Uncover Pathways that Protect against Pulmonary Hypertension in BMPR2 Mutation Carriers. Cell Stem Cell *20*, 490-504 e495.

Hayakawa, T., Kunihiro, T., Ando, T., Kobayashi, S., Matsui, E., Yada, H., Kanda, Y., Kurokawa, J., and Furukawa, T. (2014). Image-based evaluation of contraction-relaxation kinetics of human-induced pluripotent stem cell-derived cardiomyocytes: Correlation and complementarity with extracellular electrophysiology. J Mol Cell Cardiol *77*, 178-191.

Higasa, K., Miyake, N., Yoshimura, J., Okamura, K., Niihori, T., Saitsu, H., Doi, K., Shimizu, M., Nakabayashi, K., Aoki, Y., *et al.* (2016). Human genetic variation database, a reference database of genetic variations in the Japanese population. Journal of human genetics *61*, 547-553.

Higo, S., Hikoso, S., Miyagawa, S., and Sakata, Y. (2021). Genome Editing in Human Induced Pluripotent Stem Cells (hiPSCs). Methods Mol Biol *2320*, 235-245.

Hsu, P.D., Scott, D.A., Weinstein, J.A., Ran, F.A., Konermann, S., Agarwala, V., Li, Y., Fine, E.J., Wu, X., Shalem, O., *et al.* (2013). DNA targeting specificity of RNA-guided Cas9 nucleases. Nat Biotechnol *31*, 827-832.

Ito, M., Hara, H., Takeda, N., Naito, A.T., Nomura, S., Kondo, M., Hata, Y., Uchiyama, M., Morita, H., and Komuro, I. (2019). Characterization of a small molecule that promotes cell cycle activation of human induced pluripotent stem cell-derived cardiomyocytes. J Mol Cell Cardiol *128*, 90-95.

Lek, M., Karczewski, K.J., Minikel, E.V., Samocha, K.E., Banks, E., Fennell, T., O'Donnell-Luria, A.H., Ware, J.S., Hill, A.J., Cummings, B.B., *et al.* (2016). Analysis of protein-coding genetic variation in 60,706 humans. Nature *536*, 285-291.

Li, H.L., Fujimoto, N., Sasakawa, N., Shirai, S., Ohkame, T., Sakuma, T., Tanaka, M., Amano, N., Watanabe, A., Sakurai, H., *et al.* (2015). Precise correction of the dystrophin gene in duchenne muscular dystrophy patient induced pluripotent stem cells by TALEN and CRISPR-Cas9. Stem Cell Reports *4*, 143-154.

Mashiko, D., Fujihara, Y., Satouh, Y., Miyata, H., Isotani, A., and Ikawa, M. (2013). Generation of

mutant mice by pronuclear injection of circular plasmid expressing Cas9 and single guided RNA. Sci Rep *3*, 3355.

Nakagawa, M., Taniguchi, Y., Senda, S., Takizawa, N., Ichisaka, T., Asano, K., Morizane, A., Doi, D., Takahashi, J., Nishizawa, M., *et al.* (2014). A novel efficient feeder-free culture system for the derivation of human induced pluripotent stem cells. Sci Rep *4*, 3594.

Narahara, M., Higasa, K., Nakamura, S., Tabara, Y., Kawaguchi, T., Ishii, M., Matsubara, K., Matsuda, F., and Yamada, R. (2014). Large-scale East-Asian eQTL mapping reveals novel candidate genes for LD mapping and the genomic landscape of transcriptional effects of sequence variants. PLoS One *9*, e100924.

Ran, F.A., Hsu, P.D., Wright, J., Agarwala, V., Scott, D.A., and Zhang, F. (2013). Genome engineering using the CRISPR-Cas9 system. Nat Protoc *8*, 2281-2308.

Tennessen, J.A., Bigham, A.W., O'Connor, T.D., Fu, W., Kenny, E.E., Gravel, S., McGee, S., Do, R., Liu, X., Jun, G., *et al.* (2012). Evolution and functional impact of rare coding variation from deep sequencing of human exomes. Science *337*, 64-69.

van der Zwaag, P.A., Jongbloed, J.D., van den Berg, M.P., van der Smagt, J.J., Jongbloed, R., Bikker, H., Hofstra, R.M., and van Tintelen, J.P. (2009). A genetic variants database for arrhythmogenic right ventricular dysplasia/cardiomyopathy. Hum Mutat *30*, 1278-1283.
